# Heat-not-burn tobacco products: a systematic literature review

**DOI:** 10.1136/tobaccocontrol-2018-054419

**Published:** 2018-09-04

**Authors:** Erikas Simonavicius, Ann McNeill, Lion Shahab, Leonie S Brose

**Affiliations:** 1 Department of Addictions, Institute of Psychiatry, Psychology and Neuroscience, King’s College London, London, UK; 2 UK Centre for Tobacco and Alcohol Studies, UK; 3 Department of Behavioural Science and Health, University College London, London, UK

**Keywords:** electronic nicotine delivery devices, toxicology, secondhand smoke, harm reduction, tobacco industry

## Abstract

**Objective:**

To review peer-reviewed evidence on heat-not-burn tobacco products (HnB), their secondhand emissions and use by humans; to identify differences between independent and industry-funded studies.

**Data sources:**

Medline, Embase, PsycINFO, ProQuest, Scopus and Web of Science databases were searched up to 6 November 2017 for studies on HnB published after December 2009; reference lists were screened and other researchers contacted, yielding 637 records.

**Study selection:**

Thirty-one publications on HnB secondhand emissions (n=16) or use by humans (n=15) were selected by two reviewers with excellent agreement (k=0.75).

**Data extraction:**

Data on authors’ affiliations, HnB products, secondhand emissions and human exposure were extracted by one reviewer. Two reviewers assessed the quality of experimental HnB studies using the Effective Public Health Practice Project tool.

**Data synthesis:**

Twenty out of 31 studies were affiliated with tobacco industry. Studies on secondhand emissions varied by methodology, products and comparators. Compared with cigarettes, HnB delivered up to 83% of nicotine and reduced levels of harmful and potentially harmful toxicants by at least 62% and particulate matter by at least 75%. Experimental HnB use studies were limited to one product, reductions of human exposure to toxicants varied between 42% and 96%. HnB use suppressed urges to smoke, but participants rated HnB less satisfying than cigarettes. While limited by methodological heterogeneity, findings were largely similar for independent and industry-funded studies.

**Conclusions:**

Studies on HnB secondhand emissions and human use were heterogeneous and largely affiliated with the manufacturers. HnB exposed users and bystanders to toxicants, although at substantially lower levels than cigarettes.

## Introduction

‘Heat-not-burn’ tobacco products (HnB) are electronic devices that heat processed tobacco instead of combusting it to supposedly deliver an aerosol with fewer toxicants than in cigarette smoke. Commercially available HnB systems like glo (produced by British American Tobacco (BAT)) or IQOS (Philip Morris International (PMI)) include a charger, a holder and tobacco sticks, plugs or capsules. Inserted into the holder, tobacco sticks are heated with an electronically controlled heating element. Other products, like iFuse from BAT or Ploom Tech from Japan Tobacco (JT), produce vapour from a non-tobacco source and pass it through a tobacco plug to absorb flavour and nicotine.^1^ HnB products aim for a niche between combustible tobacco smoking and electronic cigarettes (e-cigarettes) that vaporise nicotine suspended in humectants.

‘Safer’ heated tobacco products that deliver nicotine but limit emissions of tar or carbon monoxide (CO) is a half-century old idea,^2^ which had been unsuccessfully market-tested since 1988, first as ‘Premier’ by the RJ Reynolds Tobacco Company (RJR) and later as ‘Eclipse’ (RJR) and ‘Accord’ (PMI).^3^ Of the current HnB products, IQOS was launched in several cities in Japan, Italy and Switzerland in 2014, iFuse was released in Romania in 2015 and glo and Ploom Tech were introduced to Japanese cities in 2016. Due to regulations restricting the sale of nicotine-containing e-cigarettes,^4^ Japan was a fertile market for HnB producers,^5^ suggesting that the products have potential ‘for explosive global growth’.^6^ By 2017, IQOS was available in 30 countries and was being considered by United States Food and Drug Administration for a reduced-risk product approval,^5^ and the UK was one of the first countries to assign a separate taxation category for HnB products.^1 7^

Committees advising the UK government carried out a systematic review of HnB studies but excluded research funded by HnB manufacturers, which comprise the majority of evidence published to date.^8 9^ Because of the paucity of evidence from independent sources, it is important to look at evidence from tobacco companies^10^ and to validate their findings.^11^ A recent Public Health England report reviewed evidence related to HnB products, including data from manufacturers.^12^ However, expeditiously published new findings call for an update. This review aimed to systematically identify and synthesise evidence from peer-reviewed studies on HnB tobacco products and to answer the following questions:

1. How do the currently researched and marketed HnB products compare with other tobacco and nicotine products:
  - in exposure to toxicants and health risks to humans through primary use and secondhand exposure?
  - in key performance characteristics (eg, nicotine delivery, use profile and user satisfaction)?
2. What is the population-level uptake of HnB products?
3. Are there any differences between independent and manufacturer-funded studies?

## Methods

### Search strategy and selection of studies

Medline, Embase, PsycINFO, ProQuest, Scopus and Web of Science databases were searched up to 13 July 2017 and the search was rerun on 6 November 2017. The full search strategy and search outcomes are reported in supplementary appendices table A1 in Supplementary file 1. The searches included terms relating to HnB in general (‘heat-not-burn’, ‘tobacco heating system’) and brand names (‘IQOS’, ‘Ploom’, ‘Heets’, ‘glo’), and were limited to studies published from 2010, to exclude papers on obsolete HnB devices. Additionally, reference lists were screened and other researchers contacted. Endnote X7 was used to record publications at all stages of the review. One reviewer (ES) screened titles and abstracts of initially included studies, and two reviewers (ES and LSB) independently screened full-text papers; Cohen’s *kappa* was calculated as a measure of agreement.

10.1136/tobaccocontrol-2018-054419.supp1 Supplementary file 1

### Inclusion criteria

The review included peer-reviewed studies that focused on HnB use by humans and the products’ health risks associated with use and secondhand exposure to HnB emissions.

### Exclusion criteria

- Publication was not peer-reviewed or was a conference abstract.
- Published before 2010 or focus was a HnB device that is no longer available (eg, Premier, Eclipse, Accord).
- Focus was not a HnB device (ie, a device did not use tobacco to produce or flavour vapour).
- Publication was not in English, French, German, Lithuanian or Russian (languages known to authors).
- Animal or in vitro study (not directly related to human use).
- Publication presented the same data as earlier publication.
- Study assessed research methodology.

### Data extraction and categorisation of included studies

The included studies were reviewed regardless of funding source. However, manufacturers that fund and report findings on their own products are inherently bound by conflict of interests. Throughout the review, funding sources of included studies were reported (table 1), and study outcomes were compared between independent and manufacturer-funded studies where the comparison was possible.

**Table 1:** Studies included in the review

	Authors, year of publication	Funder, country	Study design	Heat-not-burn and reference products	Main aim
**Studies on HnB mainstream emissions**					
1	Auer *et al*,^21^ 2017	Independent, Switzerland	Laboratory comparison study using smoking machines	IQOS Cigarette	To compare levels of HPHC in mainstream IQOS emissions with those in mainstream cigarette smoke.
2	Farsalinos *et al*,^22^2018	Independent, Greece	Laboratory comparison study using smoking machines	IQOS Cigarette E-cigarettes: (i) Ciga-like (ii) eGo-style, second generation (pen-style tank) (iii) Variable wattage (tank model)	To compare levels of nicotine in mainstream IQOS emissions from regular and menthol tobacco sticks with nicotine in different type of e-cigarettes aerosol and in mainstream cigarette smoke.
3	Bekki *et al*,^23^ 2017	Independent, Japan	Laboratory comparison study using smoking machines	IQOS Cigarette	To compare levels of nicotine and HPHC in mainstream IQOS emissions from regular and menthol tobacco sticks with those in mainstream cigarette smoke.
4	Schaller *et al*,^24^ 2016	PMI, Switzerland	Laboratory comparison study using smoking machines	THS 2.2/IQOS Cigarette	To compare levels of HPHC in mainstream IQOS emissions with those in mainstream cigarette smoke.
5	Schaller *et al*,^25^ 2016	PMI, Switzerland	Laboratory comparison study using smoking machines	THS 2.2/IQOS Cigarette	To compare levels of HPHC in mainstream IQOS emissions from regular and menthol tobacco sticks with those in mainstream cigarette smoke.
6	Jaccard *et al*,^26^ 2017	PMI, Switzerland	Laboratory comparison study using smoking machines	THS 2.2/IQOS Cigarette	To compare levels of HPHC in mainstream IQOS emissions with those in mainstream cigarette smoke.
7	Pratte *et al*,^27^ 2017	PMI, Switzerland	Laboratory comparison study using smoking machines	THS 2.2/IQOS Cigarette	To compare numbers of solid particles in mainstream IQOS emissions with those in mainstream cigarette smoke.
8	Eaton *et al*,^28^ 2018	BAT, UK	Laboratory comparison study using smoking machines	THP 1.0/glo Cigarette	To compare levels of HPHC in mainstream glo emissions with those in mainstream cigarette smoke.
9	Forster *et al*,^29^ 2018	BAT, UK	Laboratory comparison study using smoking machines	THP 1.0/glo IQOS Cigarette	To compare levels of HPHC in mainstream glo emissions with those in mainstream IQOS emissions and cigarette smoke.
10	Poynton *et al*,^30^ 2017	BAT, UK	Laboratory comparison study using smoking machines	iFuse Pen-style e-cigarette	To compare levels of HPHC in mainstream iFuse emissions with those in mainstream Vype ePen emissions and cigarette smoke.
**Studies on HnB secondhand emissions**					
11	Protano *et al*,^31^ 2016	Independent, Italy	Laboratory comparison study using smoking volunteers	THS 2.2/IQOS Cigarette Hand-rolled cigarette E-cigarette (pen-style tank)	To compare levels of secondhand emissions.
12	Protano *et al*,^32^ 2017	Independent, Italy	Laboratory comparison study using smoking volunteers	THS 2.2/IQOS Cigarette Hand-rolled cigarette Cigar Pipe E-cigarette (pen-style)	To compare levels of secondhand emissions.
13	Ruprecht *et al*,^33^ 2017	Independent, not reported	Laboratory comparison study using smoking volunteers	THS 2.2/IQOS Cigarette E-cigarette (cartridge)	To compare levels of secondhand emissions.
14	Mitova *et al*,^34^ 2016	PMI, Switzerland	Laboratory comparison study using smoking volunteers	THS 2.2/IQOS Cigarette	To compare levels of secondhand emissions.
15	O’Connell *et al*,^35^ 2015	IT, UK	Laboratory comparison study using smoking volunteers.	THS 2.2/IQOS Nicorette inhalator E-cigarette (cigalike)	To compare levels of sidestream emissions.
16	Forster *et al*,^36^ 2018	BAT, UK	Laboratory comparison study using smoking machines	THP 1.0/glo Cigarette	To compare levels of secondhand emissions.
**Studies on human use of HnB**					
17	Kamada *et al*,^39^ 2016	Independent, Japan	Case report	IQOS	To report a case of acute eosinophilic pneumonia following use.
18	Lopez *et al*,^40^ 2016	Independent, USA	Randomised crossover experimental trial	Pax LLTV Cigarette eGo e-cigarette (pen-style tank)	To compare nicotine delivery, expired air CO concentration and abstinence symptom suppression.
19	Brossard *et al*,^43^ 2017	PMI, Japan	Randomised crossover experimental trial	THS 2.2/IQOS Cigarette Nicotine gum	To compare nicotine delivery and effects on urge to smoke.
20	Haziza *et al*,^44^ 2016	PMI, Japan	RCT	THS 2.2/IQOS Cigarette	To compare exposure to HPHC during 5 days of use.
21	Haziza *et al*,^45^ 2016	PMI, Poland	RCT	THS 2.2/IQOS Cigarette	To compare exposure to HPHC during 5 days of use.
22	Lüdicke *et al*,^46^ 2017	PMI, Poland	RCT	THS 2.1 Cigarette	To compare exposure to HPHC during 5 days of use.
23	Lüdicke *et al*,^47^ 2016	PMI, Poland	RCT	CHTP Cigarettes	To compare exposure to HPHC during 5 days of use.
24	Lüdicke *et al*,^48^ 2018	PMI, Japan	RCT	THS 2.2/IQOS Cigarette	To compare exposure to HPHC during 5 days of use in confinement and further 85 days of use in an ambulatory setting.
25	Lüdicke *et al*,^49^ 2017	PMI, Japan	RCT	THS 2.2/IQOS Cigarette	To compare effect on biologically and clinically relevant risk markers during 90 days of use.
26	Picavet *et al*,^50^ 2016	PMI, UK	Randomised crossover experimental trial	THS 2.1 Cigarette	To compare nicotine delivery and effects on urge to smoke.
27	Gee *et al*,^51^ 2017	BAT, Japan	Randomised crossover experimental trial	THP 1.0/glo IQOS Cigarette	To compare the puffing topography, mouth level exposure and average daily consumption.
28	Yuki *et al*,^52^ 2017	JT, Japan	Randomised crossover experimental trial	PNTV/Ploom Tech Cigarette	To compare the pharmacokinetics of nicotine delivery.
**Studies on HnB epidemiology**					
29	Tabuchi *et al*,^41^ 2016	Independent, Japan	Epidemiological study	IQOS Ploom Tech Glo	To report awareness and use of HnB products in a nationally representative sample.
30	Tabuchi *et al*,^5^ 2017	Independent, Japan	Epidemiological study	IQOS Ploom Tech Glo	To assess population interest, rate of use, predictors of use and perceived effects of secondhand HnB aerosol.
31	Brose *et al*,^42^ 2017	Independent, UK	Epidemiological study	IQOS Ploom Tech	To assess awareness and use of HnB products in a nationally representative sample.

BAT, British American Tobacco; CHTP, carbon-heated tobacco product; HPHC, harmful and potentially harmful compounds; IT, Imperial Tobacco; JTI, Japan Tobacco International; LLTV, loose-leave tobacco vaporiser; PMI, Philip Morris International; PNTV, Prototype novel tobacco vapour product; RCT, randomised controlled trial; THP, tobacco heating product; THS, tobacco heating system.

Data on authors’ affiliations, tested HnB products, methodology, HnB sidestream, mainstream and secondhand emissions and HnB use effects on humans were extracted to a predefined table by one reviewer (ES) and checked by a second reviewer (LSB).

Tobacco products’ emissions are categorised into mainstream, sidestream and secondhand smoke. Mainstream smoke is the smoke that a user draws in,^13^ and is measured in laboratory using standardised machine smoking regimens to replicate human smoking. Sidestream smoke is emitted from the lit end of a burning tobacco product^13^ and is measured in standardised indoor and outdoor environments by recruiting smokers or using machine smoking to test the products. Secondhand smoke is the combination of exhaled mainstream and sidestream smoke.^13^ The same categorisation is used throughout the paper for emissions from e-cigarettes and HnB products. Studies were categorised to:

- Nicotine delivery and mainstream, sidestream and secondhand emissions of HnB products.
- HnB use by humans (experimental, epidemiological and case studies).

### Assessment of risk of bias

Quality of experimental studies on HnB use by humans was assessed independently by two authors (ES and LSB) using the Effective Public Health Practice Project tool.^14^ The tool evaluates quality of quantitative studies by rating study selection bias, design, confounders, blinding, data collection method and withdrawals and dropouts as weak, moderate or strong. A study is considered of strong quality if no aspect has been rated weak, moderate if one aspect has been rated weak and weak if two or more aspects have been rated weak.^14^

### Data synthesis

Findings were summarised in a narrative synthesis and quantitative results compared between studies where possible.

Studies on HnB emissions used either the International Organisation for Standardisation machine smoking regimen (ISO; 35 mL puff volume, 2 s puff duration, 30 s intervals between puffs, 14 puffs) or the Health Canada Intense regimen (HCI; 55 mL puff volume, 2 s puff duration, 30 s intervals between puffs, 14 puffs). The HCI regimen yields higher levels of harmful and potentially harmful compounds (HPHC),^15^ but no machine smoking regimen corresponds to human smoking and exposure,^16 17^ and their relevance to HnB use is not tested either. Reference products also differed between studies: the majority used 3R4F tobacco cigarettes (a reference product developed for research^18^), others used commercially available cigarettes (nicotine and CO yields provided where known) and e-cigarettes (cigalike, pen-style and tank-style).

Levels of nicotine and HPHC^19^ delivered per single HnB use to aerosol were calculated and presented as percentage of the levels in smoke from a single reference cigarette. Where data on HnB emissions were provided per puff, a single HnB use was calculated as 14 puffs with reference to ISO and HCI regimens. If independent and manufacturer-funded studies used the same puffing regimen and HnB device, nicotine and HPHC levels were compared post hoc using independent samples t-test. For studies on pharmacokinetic nicotine delivery of HnB, key pharmacokinetic characteristics were compared between HnB products where possible.

Studies on HnB use by humans were grouped by the HnB product (table 1), and findings on levels of exposure to biomarkers of HPHC (see table S1 in Supplementary file 1), nicotine delivery characteristics, human puffing topography, effect on urges to smoke and subjective satisfaction with the products were reported and compared between studies if possible.

Randomised controlled trials (RCT) used a confinement procedure: after randomisation, participants stayed at a trial site for 5 days and were restricted to only using cigarettes or HnB products, or abstained from smoking.

## Results

### Included studies

**Figure 1:**
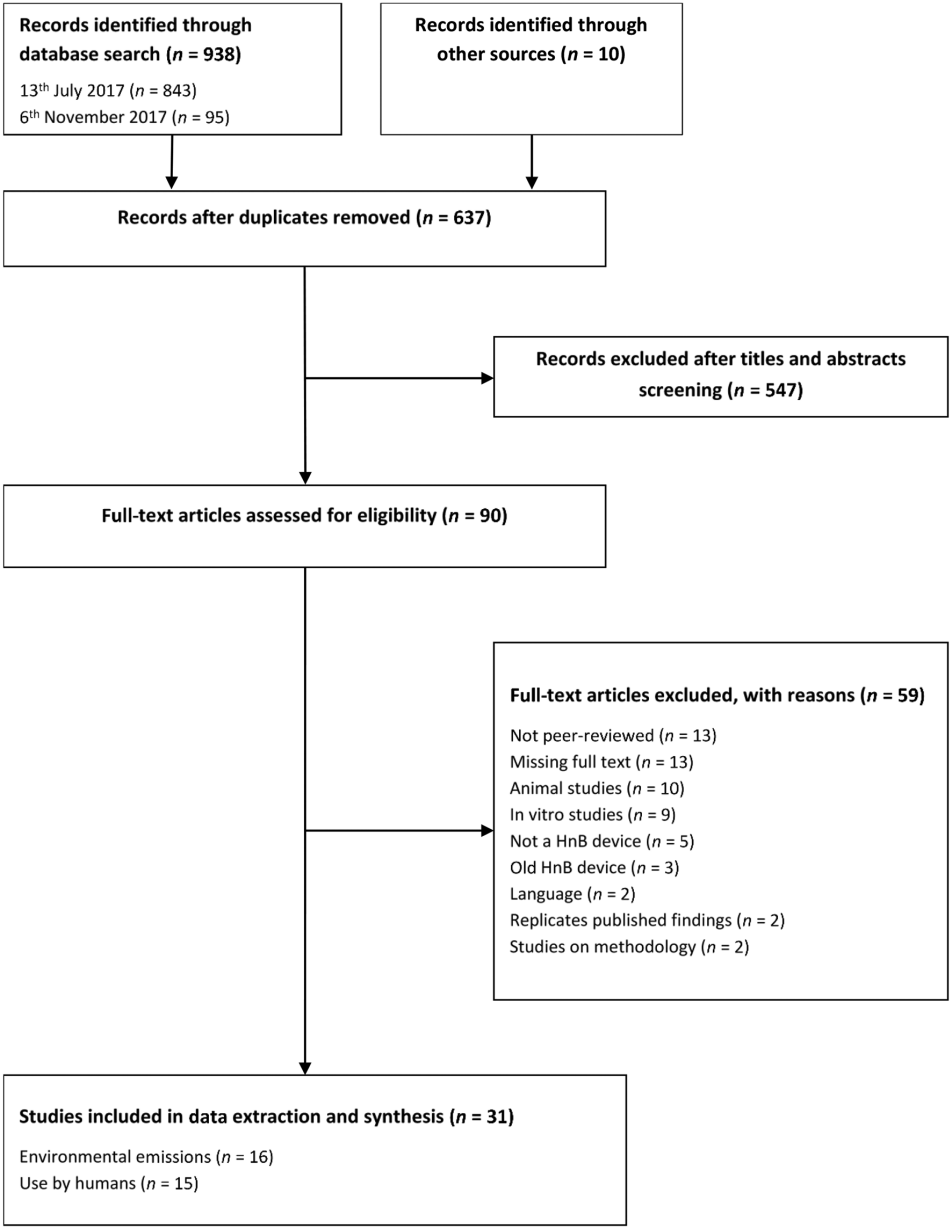
Systematic review PRISMA flow diagram.

Out of 948 initially identified records, 31 publications were included (figure 1, table 1). Reviewers’ agreement on study inclusion was excellent (k=0.75^20^). Sixteen included studies were on HnB emissions; 15 on human use of HnB products (n=21 965): of these, 11 were RCT and cross-over studies (n=1028), 3 were epidemiological studies (n=20 936) and 1 a case report (n=1). Studies reviewed seven HnB tobacco products (table 2).

**Table 2:** HnB products assessed in the included studies

HnB product and manufacturer	Release date, place	Description	Studies
Pax by Ploom (now PAX Labs)	2012, USA	Loose-leaf tobacco and cannabis vaporiser. Loose tobacco is placed into a chamber and heated by an electrically powered element.^40^ A predecessor of Ploom Tech by JTI.	40
IQOS/THS 2.2 by PMI	2014, Japan, Italy and Switzerland	IQOS includes a holder, a charger and tobacco sticks (Heets). A tobacco stick (about 320 mg) is inserted into the holder and the tobacco is heated with an electronically controlled heating blade which is inserted into tobacco plug. Operating heating temperature<350°C. A single use lasts 6 min or up to 14 puffs.^61^ Under ISO conditions, 12 puffs of the THS 2.2 yield 0.5 mg nicotine and 4.9 mg glycerol.^44^	5 21–27 29 31–35 39 41–45 48 49
iFuse by BAT	2015, Romania	iFuse includes an electronic vapour device with a rechargeable Li-ion battery and an integrated circuit power controller, onto which a cartomiser (Neopod) is attached. The disposable neopod comprises an atomiser, a liquid tank with 1.15 mL of non-flavoured nicotine liquid and a chamber containing a 130 mg tobacco plug. When the user presses a button, nicotine-containing vapour is produced, which is then drawn through the tobacco plug to absorb flavours. Before reaching the tobacco plug, the aerosol reaches an average maximum of <35°C.^30^	30
Glo/THP 1.0 by BAT	2016, Japan	Glo includes an electronic device with a rechargeable Li-ion battery and a heating chamber and tobacco sticks. A tobacco stick (about 260 mg) is heated in the heating chamber from the periphery. Operating heating temperature <250°C. Reaches operating temperature after 30–40 s and a single use lasts for another 3 min.^28^	5 28 29 36 41 51
Ploom Tech/PNTV by JTI	2016, Japan	PNTV includes a power supply unit, a cartridge with a heater and liquid and a capsule with tobacco blend. Generates a nicotine-free vapour by heating the unflavoured liquid; the vapour then passes through the tobacco capsule to absorb flavours and nicotine. Under HCI conditions, 50 puffs yield 1.10 mg nicotine.^52^	5 41 42 52
Carbon-heated tobacco product (CHTP) by PMI	Not released	A specifically designed electric lighter lights the carbon heating source which then heats a tobacco plug. Under ISO conditions, 12 puffs of the CHTP yield 0.4 mg nicotine and 2 mg glycerol, 3 mg tar and 1 mg CO.^47^ A predecessor of TEEPS by PMI.	47
IQOS/THS 2.1 by PMI	Not released	THS 2.1 includes a holder, a charger and tobacco sticks. A tobacco stick is inserted into the holder and the tobacco is heated with an electronically controlled heating blade. Operating heating temperature<400°C. A single use lasts 6 min or up to 14 puffs. Under ISO conditions, 12 puffs of the THS 2.1 yield 0.3 mg nicotine and 5 mg glycerol.^46^ A predecessor of commercially available IQOS/THS 2.2.	46 50

BAT, British American Tobacco; HnB, heat-not-burn tobacco product; ISO, International Organisation for Standardisation; PMI, Philip Morris International; PNTV, Prototype novel tobacco vapour product; JTI, Japan Tobacco International; LLTV, loose-leave tobacco vaporiser; PMI, Philip Morris International; THP, tobacco heating product; THS , tobacco heating system.

### Studies on HnB nicotine levels and emissions

The included studies^21–36^ compared HnB emissions with smoke from factory-made^21–34 36^ or hand-rolled^31 32^ cigarettes, emissions of e-cigarettes^22 30–33 35^ and nicotine inhalator^35^ (table 1). Six independent (not affiliated with manufacturers) studies^21–23 31–33^ were conducted in Switzerland,^21^ Greece,^22^ Italy^31–33^ and Japan.^23^ Ten studies funded by manufacturers of tobacco products were conducted in Switzerland^24–27 34^ and the UK.^28–30 35 36^

#### Nicotine levels in HnB tobacco sticks

Two independent studies^22 23^ reported the amount of nicotine per gram of tobacco in a regular IQOS tobacco stick (15.2±1.1 and 15.7±0.2 mg/g) and in a menthol tobacco stick (15.6±1.7 and 17.1±0.6 mg/g), respectively.

#### Nicotine levels in mainstream HnB emissions

Three independent^21–23^ and five manufacturer-funded studies^24–26 30 36^ reported on nicotine levels in mainstream HnB aerosol (table 3). One independent study^21^ used the ISO machine smoking regimen and seven used the HCI regimen.

**Table 3:** Relative nicotine delivery in mainstream HnB or e-cigarette aerosol in comparison to nicotine delivered to mainstream cigarette smoke

	Auer *et al* 21 2017*	Farsalinos *et al* 22 2018†	Bekki *et al* 23 2017	Schaller *et al* 24 2016	Schaller *et al* 25 2016	Jaccard *et al* 26 2017	Forster *et al* 36 2018	Poynton *et al* 30 2017‡
Affiliation	University of Bern, Switzerland	University of Patras, Greece	National Institute of Public Health, Japan	PMI	PMI	PMI	BAT	BAT
Reference cigarette used	Lucky Strike Blue Lights	Marlboro Regular	3R4F	3R4F	3R4F	3R4F	3R4F	3R4F
Product	Nicotine levels, % (mg)							
Reference cigarette (set as 100%)	0.361	1.99	1.70	1.89	1.88	1.86	2.02	1.84
IQOS	83% (0.30)	71% (1.41)	65% (1.10)	70% (1.32)	73% (1.38)	61% (1.14)	57% (1.16)	–
Glo	–	–	–	–	–	–	23% (0.462)	–
iFuse	–	–	–	–	–	–	–	19% (2.56/0.358)
Cigalike e-cigarette	–	43% (0.86)	–	–	–	–	–	–
Second-generation pen-style e-cigarette	–	87% (1.73)	–	–	–	–	–	27% (3.57/0.500)
Third-generation tank-style e-cigarette	–	92% (1.84)	–	–	–	–	–	–

*Provided nicotine values under ISO machine puffing regimen.

† Nicotine levels for HnB and e-cigarettes provided under 4 s puffing regimen.

**‡** Nicotine levels were provided for 100 puff blocks under 3 s puffing regimen; nicotine level for 14 puffs was calculated by multiplying the nicotine level for 100 puffs by 0.14.

–, Not measured; BAT, British American Tobacco; PMI, Philip Morris International.

Under the ISO regimen, the regular IQOS tobacco stick on average yielded 0.30 mg of nicotine,^21^ while under the HCI regimen^22–26 29 30^ nicotine levels in mainstream aerosol were 1.10–1.41 mg for IQOS, 0.46 mg for glo,^29^ and 2.56 mg per 100 puffs or 0.36 mg per single use/14 puffs for iFuse^30^ (table 3).

Compared with nicotine in smoke of reference cigarettes, nicotine in mainstream IQOS aerosol ranged from 57% to 83% across studies (table 3). One independent study^22^ reported that IQOS delivered more nicotine than a cigalike e-cigarette but less than a pen-style or a tank-style e-cigarette (table 3). A study from glo manufacturers^36^ reported that glo delivered 40% of nicotine compared with IQOS and 23% compared with reference cigarette, and a study from the manufacturer of iFuse^30^ reported that iFuse per 14 puffs delivered less nicotine than a pen-style e-cigarette (72%) and a reference cigarette (19%) (table 3).

Levels of nicotine in mainstream IQOS aerosol did not differ between independent^22 23^ and manufacturer-funded studies^24–26^ that used the HCI machine puffing regimen (1.30 vs 1.28 mg of nicotine per tobacco stick, t(17) = 0.34, p=0.74).

One independent study compared nicotine transfer rates (in this case defined as the ratio of nicotine in mainstream emissions to nicotine in a tobacco stick or a cigarette): nicotine transfer rates were higher for IQOS regular (23.4%) and menthol (23.5%) tobacco sticks than for the 3R4F reference cigarette (11.3%).^23^

#### HPHC in mainstream HnB emissions

Two independent^21 23^ and six manufacturer-funded studies^24–26 28–30^ reported levels of HPHC in mainstream HnB aerosol compared with cigarette smoke (table 4).

**Table 4:** Relative levels of HPHC in mainstream HnB aerosol compared with reference cigarette

	Schaller *et al* 25 2016	Schaller *et al* 24 2016		Jaccard *et al* 26 2017	Auer *et al* 21 2017	Bekki *et al* 23 2017		Eaton *et al* 28 2018	Forster *et al* 36 2018			Poynton *et al* 30 2017
Affiliation	PMI	PMI		PMI	University of Bern, Switzerland	National Institute of Public Health, Japan		BAT	BAT			BAT
Tobacco stick	R. IQOS	R. IQOS	M. IQOS	R. IQOS	R. IQOS	R. IQOS	M. IQOS	R. glo	R. IQOS	R. glo	M. glo	R. iFuse
Reference cigarette	3R4F	3R4F	3R4F	3R4F	Lucky Strike Blue	3R4F	3R4F	3R4F	3R4F	3R4F	3R4F	3R4F
Puffing regimen	HCI	HCI	HCI	HCI	ISO	HCI	HCI	HCI	HCI	HCI	HCI	HCI*
1,3-Butadiene	<1%	<1%	<1%	<1%	–	–	–	<1%	<1%	<1%	<1%	<1%
1-Aminonaphthalene	<1%	<1%	<1%	<1%	–	–	–	–	<1%	<1%	<1%	<1%
2-Aminonaphthalene	<1%	<1%	<1%	<1%	–	–	–	–	<1%	<1%	<1%	3%
4-Aminobiphenyl	<1%	<2%	<2%	<1%	–	–	–	–	<1%	<1%	<1%	3%
Acetaldehyde	12%	14%	13%	13%	22%	–	–	5%	15%	5%	5%	<1%
Acrolein	7%	7%	6%	6%	82%	–	–	1%	6%	1%	2%	5%
Acrylonitrile	1%	<1%	<1%	<1%	–	–	–	–	<1%	<1%	<1%	<1%
Ammonia	38%	36%	35%	36%	–	–	–	–	33%	12%	15%	<50%
Benzene	<1%	<1%	<1%	<1%	–	–	–	<1%	<1%	<1%	<1%	<1%
Benzo[a]pyrene	7%	9%	8%	6%	4%†/8%‡	–	–	<3%	5%	2%	3%	<7%
Carbon monoxide	1%	2%	2%	1%	–	1%	1%	<1%	1%	<1%	<1%	21%
Crotonaldehyde	<6%	6%	5%	<6%	4%	–	–		5%	1%	2%	<3%
Formaldehyde	11%	10%	8%	9%	74%	–	–	6%	11%	6%	7%	13%
Isoprene	<1%	<1%	<1%	<1%	–	–	–	–	<1%	<1%	<1%	<1%
NNN	5%	6%	4%	4%	–	6%	8%	9%	4%	9%	7%	<1%
NNK	3%	3%	2%	3%	–	5%	5%	2%	4%	2%	2%	<1%
Toluene	2%	1%	1%	1%	–	–	–	–	1%	<1%	<1%	2%
Nicotine	73%	70%	64%	61%	84%	65%	71%	–	57%	23%	18%	139%
Water	203%	231%	188%	–	–	328%	350%	–	168%	80%	71%	–
Glycerol	203%	191%	163%	–	–	–	–	–	182%	129%	101%	–
Total particulate matter	122%	98%	89%	–	–	119%	135%	–	104%	56%	54%	–
Tar/nicotine-free dry particulate matter	79%	33%	40%	–	–	39%	53%	–	75%	46%	48%	–

*Puffing duration increased to 3 s, levels of HPHC for HnB product measured for 100 puffs.

†Originally reported proportions of HnB relative to polycyclic aromatic hydrocarbons in mainstream smoke of 50 commercial US cigarettes.

‡Proportions recalculated using mean values of polycyclic aromatic hydrocarbons in mainstream smoke of 50 commercial US cigarettes measured by ISO smoking regimen.^15^

BAT, British American Tobacco; HnB, heat-not-burn tobacco product; HPHC, harmful and potentially harmful compounds; ISO, International Organisation for Standardisation; M, menthol; NNK, nicotine-derived nitrosamine ketone; NNN, N-nitrosonornicotine; PMI, Philip Morris International; R, regular; –, not measured.

One independent study^21^ used data of 50 US cigarette brands^15^ to compare levels of polycyclic aromatic hydrocarbons but, as a critique by PMI noted,^37^ the authors had inadvertently used reference values obtained under HCI instead of ISO regimen. We provide both the originally published and recalculated ratios (table 4).

The study assessing iFuse^30^ calculated HPHC yield per 100 3 s puffs on iFuse but followed an HCI regimen for the reference cigarette, creating discrepancies in comparison with other studies (table 4).

Compared with cigarettes, under HCI regimen machine-derived mainstream HnB emissions contained lower levels of nicotine (18%–73% of those in cigarette smoke), CO (reduction ≥98%), HPHC (reduction ≥62%) and tar (reduction ≥21%) (table 4).

One independent^23^ and three manufacturer-funded studies^24–26^ used the HCI machine-puffing regimen and reported findings on the same HPHC in mainstream IQOS emissions. Levels of CO (t(11)=1.28, p=0.23), water (t(8)=0.43, p=0.68) and total particulate matter (t(8)=1.77, p=0.11) did not differ statistically significantly between independent and manufacturer-funded studies. Compared with manufacturer-funded studies, the independent study reported less tar (9.8 vs 15.0 mg, t(8)=4.8, p=0.001) and more tobacco-specific nitrosamines (19.2 vs 14.2 ng of N-nitrosonornicotine (NNN), t(11)=7.7, p<0.001; 12.3 vs 6.8 ng of nicotine-derived nitrosamine ketone (NNK), t(11)=11.8, p<0.001; 4.5 vs 3.0 ng of N-nitrosoanabasine (NAB), t(4)=5.1, p=0.007; 34.0 vs 19.2 ng of N-nitrosoanatabine (NAT), t(8)=13.2, p<0.001) in mainstream IQOS aerosol from a single tobacco stick.

#### Particulate matter and HPHC in sidestream and secondhand HnB emissions

Seven studies, three independent^31–33^ and four funded by tobacco manufacturers,^27 34–36^ compared HnB sidestream or secondhand emissions with smoke of factory-made^27 31–34 36^ or hand-rolled^31 32^ cigarettes, pipes and cigars,^32^ aerosol from a nicotine inhalator^35^ or e-cigarettes.^31–33 35^

A single study^35^ funded by Imperial Tobacco company that does not manufacture HnB products^38^ explicitly focused on sidestream emissions of a competitor’s HnB product (IQOS). The study concluded that in contrast to a cigalike e-cigarette and a nicotine inhalator, IQOS produced sidestream emissions. Similarly, an independent study^32^ concluded that higher particulate matter emissions from IQOS than from a pen-style e-cigarette could be explained by sidestream emissions.

Six studies, three independent^31–33^ and three manufacturer-funded,^27 34 36^ reported on particulate matter in HnB secondhand emissions. One study^31^ reported that a pen-style e-cigarette and IQOS emitted 25% of the total particulate matter detected in smoke from a cigarette. Use of the e-cigarette produced higher peak concentration of particles in the air than use of IQOS, but the total amount and time for particles to disperse after use were longer for IQOS and composition of particles was not considered.^32^ Most particles emitted by IQOS were <1000 nm,^33^ and particles emitted by glo were in the same size range (150–250 nm diameter) as particles in cigarette smoke^36^ (table 5). Compared with reference cigarettes, particle mass in emissions from an e-cigarette and IQOS were <2%^33^ and from glo was <1%.^36^ Two studies by the manufacturer of IQOS^27 34^ did not detect particulate matter in IQOS mainstream and secondhand emissions which was at odds with findings from independent studies.^27 34^

**Table 5:** Relative levels of HPHC and particulate matter in secondhand emissions from HnB products (ratio HnB:reference cigarette)

	Ruprecht *et al* 33 2017	Mitova *et al* 34 2016	Forster *et al* 36 2018b
Affiliation	National Cancer Institute, Milan, Italy	PMI	BAT
HnB	IQOS	IQOS	Glo
Reference cigarette	Conventional cigarette	Marlboro Gold	Lucky Strike Regular
Setting	‘A sitting room’ (ACH=1.5)	‘Residential’ (ACH=1.2)	‘Home’ (ACH=1.2)
Secondhand emissions’ markers			
370 nm UV BC (µg/m^3^)	0.7%–0.8%	–	–
PM>0.3 (particles/cm^3^)	2.8%–7.3%	–	–
PM_nm_ (particles/cm^3^)	22.0%–24.0%	–	–
PM 1 (µg/m^3^)	0.9%–1.0%	–	HnB < background
PM 2.5 (µg/m^3^)	1.3%–1.5%	Non-detectable	HnB < background
PM 10 (µg/m^3^)	1.5%–1.7%	–	HnB < background
Ultraviolet particulate matter	–	Non-detectable	–
Fluorescent particulate matter	–	Non-detectable	–
Solanesol	–	Non-detectable	–
3-Ethenylpyridine	–	Non-detectable	Non-detectable
HPHC			
1,3-Butadiene	–	Non-detectable	Non-detectable
Acetaldehyde (µg/m^3^)	5.0%–5.9%	6.0%	2.2%
Acrolein (µg/m^3^)	1.8%–2.3%	Non-detectable	Non-detectable
Acrylonitrile	–	Non-detectable	Non-detectable
Benzene	–	1.7%	HnB = background
Carbon monoxide	–	3.8%	Non-detectable
Crotonaldehyde	–	Non-detectable	Non-detectable
Formaldehyde (µg/m^3^)	6.9%–7.1%	7.6%	10.2%
Isoprene	–	HnB < background	HnB < background
Toluene	–	HnB < background	3.7%
Nicotine	–	6.2%	HnB < background
Nitrogen oxides	–	HnB < background	HnB < background
Nitrogen oxide	–	HnB < background	HnB < background

ACH, air changes per hour (ventilation rate of an indoor space defined as air volume added/removed from the space in  1  hour   divided by the space volume); BAT, British American Tobacco; HnB,  heat-not-burn tobacco product;  HPHC,   harmful and potentially harmful compounds;   –, not measured; PM > 0.3, particulate matter larger than 0.3 µm; PM _nm_, particulate matter in size range of  10–1000   nm; PMI, Philip Morris International; UV BC, ultraviolet black carbon.

Three studies, one independent^33^ and two manufacturer-funded,^34 36^ also reported on HPHC in secondhand emissions (table 5). All studies detected HPHC in air after HnB use; HPHC levels in HnB secondhand emissions were lower than in cigarette smoke, but reported content of the emissions varied (table 5). Methods of the independent and manufacturer-funded studies were heterogeneous to make direct comparisons. However, the independent study^33^ detected particulate matter and acrolein in IQOS secondhand emissions when the manufacturer-funded study^34^ did not detect these.

### Studies on HnB use by human participants

The 15 studies on HnB use by humans (5 independent^5 39–42^ and 10 manufacturer-funded^43–52^ (table 1) included 5 RCTs (one published in two parts),^44–49^ 5 cross-over studies^38 39 47 48 50^, 1 case report^39^ and 3 epidemiological studies.^5 41 42^

#### Loose-leaf tobacco vaporiser (Pax)

An independent study^40^ compared Pax, a cigarette and a pen-style e-cigarette on nicotine delivery to blood plasma, expired air CO, suppression of nicotine abstinence symptoms and satisfaction. Based on the research quality rating tool, the study was rated weak (see table S3 in Supplementary file 1). Plasma nicotine levels were 24.4 ng/mL after cigarette use, 14.3 ng/mL after use of Pax and 9.5 ng/mL after use of the e-cigarette. Expired air CO increased up to 16.9 parts per million (ppm) after smoking a cigarette but decreased after HnB and e-cigarette use to 4.5 ppm. Nicotine abstinence symptoms were most effectively suppressed after smoking a cigarette, use of Pax was less effective and e-cigarette use was least effective; no differences were observed between conditions. Study participants found the HnB and the e-cigarette significantly less satisfying than cigarettes.^40^

#### Carbon-heated tobacco product

One manufacturer-funded 5-day confinement RCT of moderate quality^47^ compared levels of exposure with HPHC between smokers who were randomised to using carbon-heated tobacco product (CHTP) only, continued smoking or abstinence (see table S3 in Supplementary file 1; table 6).

**Table 6:** Product use and ratio of levels of exposure to HPHC in HnB users compared with cigarette smokers on the fifth day of confinement

	Lüdicke *et al* 47 2016	Lüdicke *et al* 46 2017	Haziza *et al* 44 2016	Haziza *et al* 45 2016	Lüdicke *et al* 48 49 2018
Affiliation	PMI	PMI	PMI	PMI	PMI
HnB product	CHTP	THS 2.1	Regular IQOS	Regular IQOS	Menthol IQOS
Reference product	Regular cigarette	Regular cigarette	Regular cigarette	Regular cigarette	Menthol cigarette
Mean (SD) HnB vs cigarettes use on day 5	19.7 (7.8) vs 18.8 (4.4)	27.2 (9.1) vs 20.1 (3.2)	9.9 (3.9) vs 12.5 (3.5)	20.7 (8.1) vs 16.6 (3.8)	13.9 (4.3) vs 13.6 (4.7)
Exposure to HPHC % (95% CI) HnB:cigarettes ratio					
1,3-Butadiene	10%	12% (9% to 16%)	23% (18% to 29%)	8% (7% to 10%)	13%
1-Aminonaphthalene	–	–	4% (4% to 5%)	4% (3% to 5%)	6%
2-Aminonaphthalene	19%	11% (8% to 14%)	18% (15% to 21%)	12% (10% to 13%)	14%
4-Aminobiphenyl	16%	41% (31% to *53%)	18% (15% to 22%)	15% (13% to 17%)	21%
Acetaldehyde*	–	–	–	–	–
Acrolein	26%	28% (23% to 33%)	53% (46% to 61%)	42% (38% to 46%)	52%
Acrylonitrile	–	15% (12% to 18%)	21% (18% to 25%)	13% (12% to 15%)	18%
Ammonia*	–	–	–	–	–
Benzene	16%	7% (5% to 10%)	16% (13% to 19%)	6% (5% to 7%)	11%
Benzo[a]pyrene	–	–	30% (25% to 36%)	28% (23% to 33%)	28%
Carbon monoxide	39%	23% (21% to 26%)	47% (44% to 50%)	24% (22% to 25%)	45%
Crotonaldehyde	–	–	38% (32% to 45%)	23% (20% to 25%)	43%
Formaldehyde*	–	–	–	–	–
Isoprene*	–	–	–	–	–
N-nitrosonornicotine		12% (9% to 16%)	30% (24% to 38%)	24% (18% to 33%)	29%
Nicotine-derived nitrosamine ketone	52%	33% (25% to 44%)	49% (42% to 57%)	44% (39% to 48%)	44%
Toluene*	–	–	–	–	–
Nicotine	–	85% (62% to 115%)	113% (91% to 140%)‡/89.6%§	113% (91% to 140%)	–
Nicotine equivalents	111%	87% (76% to 100%)	105% (92% to 120%)‡/98.6%§	105% (92% to 120%)	118%
Cotinine	110%	88% (75% to 103%)	96% (71% to 131%)	111% (91% to 136%)	–
Ethylene oxide	–	–	47% (40% to 55%)	32% (27% to 38%)	51%
Pyrene	57%	43% (36% to 51%)	46% (41% to 52%)	44% (40% to 49%)	38%
o-Toluidine	49%	58% (48% to 71%)	51% (42% to 60%)	42% (36% to 48%)	41%

*Exposure to acetaldehyde, ammonia, formaldehyde, isoprene and toluene was not measured due to absence of valid biomarkers.

†Originally reported proportions.

‡Proportions calculated based on raw study figures.

HnB,   heat-not-burn tobacco product;   HPHC,     harmful and potentially harmful compounds; –, not measured; PMI ,   Philip Morris International.

Compared with participants who continued smoking, on day 5, CHTP users demonstrated less exposure to HPHC, took more frequent and longer puffs that were of higher average and total volume. Differences in CHTP and cigarette use frequency (19.7 vs 18.8 on day 5, respectively, p=0.57), total nicotine equivalents (19.1 vs 17.2 ng/mL) and plasma cotinine for the past 24 hours (319.8 vs 289.8 mg) were not statistically significant.

#### Tobacco heating system 2.1

Two manufacturer-funded studies of moderate^46^ and weak quality^50^ assessed tobacco heating system 2.1 (THS 2.1) (table 6, table S3 in Supplementary file 1).

##### Nicotine delivery

One manufacturer-funded study^50^ compared the nicotine delivery of THS 2.1 use and cigarette smoking (table S2 in Supplementary file 1); after single use, they were similar in how fast plasma nicotine levels peaked (8 min median for both), reducing urges to smoke based on Questionnaire of Smoking Urges scores (THS 2.1 reduced by 19.4±22.4, cigarette by 19.5±23.1) and in the nicotine half-life length (2.6 vs 2.5 hours). Compared with cigarettes, THS 2.1 delivered lower peak levels of nicotine after single (8.4 vs 11.9 ng/mL) and ad libitum use (14.9 vs 24 ng/mL).

THS 2.1 users also consumed fewer tobacco sticks per day than smokers smoked cigarettes (10.9 vs 16.7, p<0.001) and perceived THS 2.1 less satisfying than cigarettes: rated it lower on four out of five modified cigarette evaluation scores (mCEQ)^53^ subscales (smoking satisfaction, psychological rewards, enjoyment of respiratory tract sensation and craving reduction).

##### Exposure to HPHC

A manufacturer-funded 5-day confinement RCT^46^ compared exposure levels to HPHC in smokers who were randomised to using only THS 2.1 or continued smoking. Exposure to HPHC was lower in the THS 2.1 group (table 6).

The THS 2.1 group used up to 35% more tobacco sticks than the smoking group smoked cigarettes (27.2 and 20.1, respectively, p=0.002) and demonstrated compensatory puffing (increased puff frequency, duration and volume). On day 5, the THS 2.1 group achieved 85% of nicotine and 88% of cotinine levels of the smoking group. Satisfaction with THS 2.1 was again significantly lower on the same four mCEQ subscales.

#### Tobacco heating system 2.2 (IQOS)

Five studies funded by the manufacturer of IQOS (PMI)^43–45 48 49^ assessed IQOS (table 1).

##### Nicotine delivery

A study^43^ of weak quality (see table S3 in Supplementary file 1) compared nicotine delivery between regular IQOS and regular cigarettes, menthol IQOS and menthol cigarettes and IQOS tobacco sticks and 2 mg nicotine gum. Peak plasma concentrations for both IQOS tobacco sticks and cigarettes were reached in 6 min, actual exposure to nicotine was comparable (IQOS:cigarettes ratio was 96.3% for regular and 98.1% for menthol), as was nicotine half-life (93.1% and 102.3%). Peak nicotine concentration ratio for regular IQOS versus cigarettes was 103.5% and for menthol IQOS versus menthol cigarettes, 88.5%. Compared with nicotine gum, the results were less clear: regular IQOS outperformed menthol IQOS for exposure to nicotine (127.2% and 55.9%) and peak nicotine concentration (240.2% and 101.6%). Relative to the gum, nicotine half-life was 87.3% for regular and 92.1% for menthol IQOS tobacco sticks.

##### Exposure to HPHC

Two 5-day confinement RCTs^44 45^ (one strong, one moderate quality; table S3 in Supplementary file 1) assessed exposure to HPHC in smokers randomised to using IQOS, continuing smoking or abstaining from smoking for study period (table 6). Two publications^48 49^ (moderate quality, table S3 in Supplementary file 1) reported on the same 5-day confinement RCT followed by 85 days in an unconfined setting. Exposure to HPHC and change in health risk markers were compared between smokers randomised to using menthol IQOS, continuing smoking or abstaining from smoking for study period (table 6).

Across the three studies, exposure to biomarkers of HPHC in the IQOS groups was lower than for smoking groups and approached exposure levels observed in abstinent groups.

Daily product use differed across studies: on day 5 the IQOS group in one study^44^ used 20% fewer tobacco sticks than the smokers’ group smoked cigarettes (p<0.001); in another study,^45^ they used 25% more tobacco sticks than the smoking group smoked cigarettes (p<0.001) and in the third study,^48^ consumption did not differ between groups (p=0.63). During an unconfined study period, participants in the IQOS group demonstrated high compliance (89.7%), and dual use of cigarettes and IQOS was low (<0.1 cigarette on average in the IQOS group).^48^

Throughout all three studies, IQOS users increased their puffing frequency, duration and number of puffs. IQOS use suppressed urges to smoke similarly to smoking cigarettes but was consistently rated lower on sensory and psychological satisfaction than cigarettes: in two studies, IQOS scored lower on four out of five mCEQ subscales^45 48^ and in one study IQOS scored lower on the smoking satisfaction subscale.^44^

Over the 85 days following the 5-day confinement period, participants randomised to IQOS use demonstrated reductions in risk markers^54^ associated with endothelial dysfunction, oxidative stress, inflammation and high-density lipoprotein cholesterol counts compared with participants randomised to continued smoking.^49^

#### Tobacco heating product 1.0 (glo)

A manufacturer-funded randomised cross-over trial^51^ (weak quality, table S3 in Supplementary file 1) compared puffing topography, mouth level exposure and daily consumption of glo among cigarette smokers and smokers who dually used IQOS but were naïve to glo. In comparison to cigarette smoking, glo and IQOS users demonstrated significantly higher mean puff volumes (66.7 and 63.5 mL, respectively vs 48.9 mL for a cigarette), shorter puffing intervals (7.4 s and 8.3 s vs 9.7 s), and used glo and IQOS less frequently than cigarettes (12.1 and 13.7 tobacco sticks, respectively vs 16.3 cigarettes). Users of glo and IQOS differed in mean puff volume and average daily consumption: new glo users demonstrated higher mean puff volume (60.9±24.8 vs 55.1±23.9 mL) and used fewer tobacco sticks than the IQOS users (11.2±6.2 vs 13.4±7.8).

#### Prototype novel tobacco vapour product (Ploom Tech)

A manufacturer-funded randomised cross-over trial^52^ (weak quality, table S3 in Supplementary file 1) compared the pharmacokinetic profiles of Ploom (table S2 in Supplementary file 1) and a reference cigarette. They did not differ in the time to reach peak plasma nicotine concentration (median for both 3.8 min) or in nicotine half-life after single use (1.66 for Ploom vs 1.86 hour for a cigarette). However, Ploom delivered significantly lower peak plasma nicotine concentration (45.7% of cigarette) and total exposure to nicotine after single use (68.3%, p=0.002).

### Epidemiological studies on HnB use

The literature search identified three independently funded surveys on awareness and use of HnB products: two from Japan^5 41^ and one from the UK.^42^

The studies from Japan reported findings from a nationally representative sample of 8240 respondents aged 15–69 years first surveyed in 2015^41^ and followed up in 2016 (follow-up rate 65.6%) and 2017 (52.2%).^5^ The data suggest growth in IQOS use: in 2015 0.3% reported using IQOS in the last 30 days, in 2016 this rose to 0.6%, and in 2017 to 3.6%.^5^ The use of other HnB products (not mutually exclusive) in 2017 was lower: 1.2% had used Ploom in the last 30 days (a rise from 0.3% in 2015) and 0.8% had used glo in the last 30 days (no data for previous years).^5^ The same study reported that among 11.9% of the sample that had been exposed to HnB secondhand emissions, more than a third (37%) experienced at least one symptom (eg, sore throat, eye pain, feeling ill, etc) related to this exposure.

Data from a nationally representative sample of 12 696 adults on awareness and use of HnB products in Great Britain (GB) were collected in March–April 2017^42^; 9.3% of the adult GB population were aware of HnB and 1.7% had tried or were using the products. Among those who had ever tried HnB, 39% had tried it once or twice and 13% had been using it daily. It should be noted, however, that participants were asked about HnB products prior to answering about e-cigarettes, which could have led to overestimating awareness and use of HnB products.

### Case report on HnB use

A single case report^39^ of acute eosinophilic pneumonia (AEP) following the use of HnB was identified. The study summarised the case of a Japanese man aged 20 years who used 20 HnB tobacco sticks per day for 6 months and increased to using 40 tobacco sticks 2 weeks before hospitalisation. Based on the relationship between cigarette smoking and AEP, the authors presumed that in this case the rapid increase in the daily use of tobacco sticks caused the onset of AEP. The case report concludes that despite HnB users being exposed to lower levels of HPHC compared with cigarette smokers, they are still susceptible to health risks in general and to acute eosinophilic pneumonia in particular.

## Discussion

The systematic search identified 31 peer-reviewed studies on seven HnB products. Eleven independent studies focused on awareness and use and secondhand emissions of HnB products, while 20 manufacturer-funded studies explored nicotine delivery and mainstream emissions and conducted RCTs assessing exposure to HPHC in HnB users.

By late 2017, awareness and use of HnB products were rising in Japan while in GB HnB use was rare.

Five RCTs demonstrated that switching from smoking cigarettes to using HnB significantly reduces but does not eliminate exposure to HPHC. The evidence, however, was limited to one currently available HnB product and came from a single tobacco manufacturer.

Single use of HnB delivered nicotine as quickly as smoking a cigarette but with lower peak concentration and total exposure to nicotine. When used ad libitum, HnB delivered comparable levels of nicotine and weakened urges to smoke similarly to cigarettes, nevertheless, HnB users reported compensatory puffing and consistently rated HnB less rewarding and satisfying than cigarettes.

Studies on machine-generated mainstream HnB emissions generally reported higher proportional reductions in exposure to HPHC than were observed in the RCTs on HnB use by humans. This suggests that machine smoking does not reliably replicate human use; this has been demonstrated for cigarettes^16^ and it is likely even less reliable for HnB products.

The tested HnB products were heterogeneous in the way they worked and in the levels of nicotine and HPHC they delivered to mainstream aerosol. Compared with cigarettes, aerosol of IQOS (heating tobacco up to 350°C) contained the highest proportional levels of nicotine and HPHC, followed by glo (250°C) and iFuse (35°C), which produced the least toxicants and delivered the lowest levels of nicotine of the three. Compared with e-cigarettes, IQOS delivered less nicotine than a tank-style but more than a cigalike e-cigarette.

Evidence on HnB secondhand emissions suggested that HnB exposes users and bystanders to substantially lower but measurable levels of particulate matter and HPHC.

Comparisons between findings of independent and manufacturer-funded studies were limited due to heterogeneity in methods measuring mainstream and sidestream emissions or lack of independent evidence on HnB use by humans. Where the comparisons were possible, sample sizes were low and assumptions for t-tests could not be verified. Independent and manufacturer-funded studies reported similar levels of nicotine, CO, water and total particulate matter in mainstream IQOS aerosol, but diverged when reporting on tar (an independent study reported less) and tobacco-specific nitrosamines (manufacturer-funded studies reported less). Inferences from these comparisons are limited, as only two independent studies provided data on a limited number of HPHC. Conclusions on secondhand emissions from HnB devices were at odds between independent and manufacturer-funded studies, with PMI-funded studies reporting no particulate matter in IQOS secondhand emissions.

### Limitations of the present evidence

Out of 11 trials on HnB use by humans, only 1 was not affiliated with a tobacco manufacturer^40^; the lack of independent evidence that could validate manufacturer data remains a major limitation.

Other limitations include that none of the trials on HnB use by humans registered a protocol before the enrolment of the first participant as recommended by the International Committee of Medical Journal Editors.^55^ Other concerns that were raised in relation to studies funded by the manufacturer of IQOS pertained to the quality of their trials,^56^ possible tobacco industry ties of the journal that published most studies on IQOS characteristics^57 58^ and non-reporting levels of all 93 HPHC and other potentially harmful constituents in mainstream and sidestream emissions.^59^

Some manufacturer-funded publications appeared to overstate conclusions. For instance, studies concluded that IQOS was comparable in satisfaction to smoking^44 48^ when it was repeatedly rated lower on four out of five mCEQ subscales.^53^ Studies that reported similar findings on HnB secondhand emissions differed in their conclusions: manufacturer-funded studies concluded that HnB use impact on indoor air quality was negligible^34^ or that HnB emissions were less harmful than cigarette smoke,^36^ while an independent study concluded that despite lower emissions, HnB still pose evident risks through secondhand emissions.^33^ Only one trial assessed health effects of long-term HnB (IQOS) use relative to abstinence and continued smoking.^49^ However, the validity of the study follow-up results and conclusions are reduced by a lack of validation of self-reported abstinence and adherence to study condition.

### Limitations of the systematic review

The review included data from manufacturer-funded studies but excluded reports or papers that were not peer-reviewed. As HnB manufacturers not always publish in peer-reviewed journals, this might have limited scope of our study. Nevertheless, this review provides the first comprehensive summary of up-to-date evidence on HnB tobacco products.

### Future research

Machine smoking regimens were tailored for testing and comparing emissions from different tobacco cigarettes, and the validity of this method to measure emissions from HnB products is unclear. Future research should clarify whether the existing regimens reliably estimate HnB emissions or need adjusting. Until then, to promote reproducibility and comparisons between studies, research on HnB emissions would benefit from employing standardised protocols: by using the same machine smoking regimen (eg, HCI or other, adjusted for HnB), same reference products (eg, 3R4F tobacco cigarette) and screen for the same list of HPHC.

Current evidence on HnB sidestream emissions comes from a single manufacturer-funded study that is subject to conflict of interests; independent research could disentangle the preliminary disagreement.

Although research on mainstream and sidestream HnB emissions provides valuable preliminary data on HnB characteristics, it is unclear how well these findings represent the actual health risks of HnB use. The discrepancies in exposure to HPHC between machine puffing and human use studies suggest that findings on HnB mainstream emissions underestimate the actual exposure to toxicants. Instead of measuring HPHC levels in mainstream HnB emissions, independent research should prioritise validating manufacturers’ findings on exposure to toxicants in HnB use by humans and comparing actual long-term health effects of HnB use with health outcomes of smoking, vaping or using nicotine replacement therapy.

Recently, HnB tobacco products have been introduced to multiple tobacco markets around the world, but only three independent studies from Japan and GB reported on awareness and use. There is a need for future surveillance on the uptake of HnB products and comparisons between countries with different regulatory frameworks for tobacco and nicotine products.

As more HnB products appear on the market, more manufacturer-funded studies are expected. This challenges independent researchers to critically evaluate and validate industry findings.^11^ All researchers, whether affiliated with tobacco manufacturers or not, should aim for professional and transparent ways to preregister, conduct and report their findings.^60^

## Conclusion

Peer-reviewed evidence on heated tobacco products indicates that HnB are effective nicotine delivery devices that expose users and bystanders to substantially fewer harmful and potentially harmful compounds than smoking cigarettes. The evidence is primarily drawn from tobacco industry data and lacks research on long-term HnB use effects on health. The HnB harm profile needs to be confirmed by independent research and compared with other alternative nicotine products that have reduced health risk exposure profiles.
