# Supplementary material for: Heat-not-burn tobacco products: a systematic literature review
Source: Tob Control. 2018 Sep 4;28(5):582–94. doi: 10.1136/tobaccocontrol-2018-054419 (PMC6824610; doi:10.1136/tobaccocontrol-2018-054419)
Supplement: Supplementary file 1 [file tobaccocontrol-2018-054419supp001.pdf]

Supplementary material and appendices for
Heat-not-burn tobacco products: a systematic literature review

Erikas Simonavicius[1], Ann McNeill[1, 2], Lion Shahab[3], Leonie S Brose[1, 2]

[1] King's College London, Department of Addictions, Institute of Psychiatry, Psychology and Neuroscience, 4 Windsor Walk, London, SE5 8BB, United Kingdom

[2] UK Centre for Tobacco and Alcohol Studies, United Kingdom

[3] University College London, Behavioural Science and Health Institute of Epidemiology & Health, London, United Kingdom

Correspondence to:
Erikas Simonavicius, Department of Addictions, Institute of Psychiatry, Psychology and Neuroscience, King's College London, 4 Windsor Walk, London SE5 8BB, United Kingdom. E-mail: erikas.simonavicius@kcl.ac.uk

Supplementary tables

Table S1 Toxicants, carcinogens and other compounds, and their related biomarkers of exposure measured in human exposure studies 2

Table S2 Nicotine delivery after use of a regular tobacco stick 2

Table S3 Quality rating of randomised controlled trials and crossover studies 3

Table S1 Toxicants, carcinogens and other compounds, and their related biomarkers of exposure measured in human exposure studies [1]

Harmful and potentially harmful compounds (HPHC)	Risk	Measured biomarker of exposure
1,3-butadiene	Carcinogen, respiratory & reproductive/developmental toxicant	Monohydroxybutenyl mercapturic acid (MHBMA)
1-aminonaphthalene	Carcinogen	1-aminonaphthalene (1-NA)
2-aminonaphthalene	Carcinogen	2-aminonaphthalene (2-NA)
4-aminobiphenyl	Carcinogen	4-aminobiphenyl (4-ABP)
Acetaldehyde	Carcinogen, respiratory toxicant & addictive	No valid biomarker
Acrolein	Respiratory & cardiovascular toxicant	3-hydroxypropylmercapturic acid (3-HPMA)
Acrylonitrile	Carcinogen, respiratory toxicant	2-cyanoethylmercapturic acid (CEMA)
Ammonia	Respiratory toxicant	No valid biomarker
Benzene	Carcinogen, cardiovascular & reproductive/developmental toxicant	S-phenylmercapturic acid (S-PMA)
Benzo(a)pyrene	Carcinogen	3-hydroxy-benzo(a)pyrene (Total-3-OH-B[a]P)
Carbon monoxide	Reproductive/developmental toxicant	Carboxyhemoglobin (COHb)
Crotonaldehyde	Carcinogen	3-hydroxy-1-methylpropylmercapturic acid (3-HMPMA)
Formaldehyde	Carcinogen & respiratory toxicant	No valid biomarker
Isoprene	Carcinogen	No valid biomarker
N-nitrosonornicotine (NNN)	Carcinogen	Total N-nitrosonornicotine (NNN)
4-(methylnitrosamino)-1-(3-pyridyl)-1-butanone (NNK)	Carcinogen	Total 4-(methylnitrosamino)-1-(3-pyridyl)-1-butanol (NNAL)
Toluene	Respiratory & reproductive/developmental toxicant	S-benzylmercapturic acid (S-BMA)
Nicotine	Reproductive/developmental toxicant & addictive	Total nicotine equivalents in urine (free nicotine, nicotine-glucuronide, free cotinine, cotinine-glucuronide, free trans-3’-hydroxycotinine, trans-3’-hydroxycotinine-glucuronide)

Table S2 Nicotine delivery after use of a regular tobacco stick

Pharmacokinetic parameters	THS 2.1 [2]	THS 2.2 [3]*	PNTV product [4]**
C_{max} (ng/mL)	8.4 (6.8–10.3)	14.3; 11.53	5.39 (4.34, 6.69)
$AUC_{0\text{-last}}$ (ng*h/mL)	17.7 (15.0–20.8)	23.75; 18.92	4.12 (3.43, 4.95)
$t_{1/2}$ (h)	2.6 (2.3–3.0)	3.81; 4.16	1.66 (1.41, 1.95)
t_{max} (min)	8 (4–61)	6; 6	3.86 (2.83–7.83)

* Two reported least square means are from THS 2.2 comparison with cigarette and with nicotine gum, respectively
** As the product does not contain nicotine sticks, it was used for 3 minutes, 10 puffs at approximately 20 sec intervals
C_{max}: maximum observed plasma concentration; $AUC_{0\text{–last}}$: area under the plasma concentration-time curve from time 0 to the last quantifiable concentration; $t_{½}$: terminal elimination half-life; t_{max}: time to C_{max}.

Table S3 Quality rating of randomised controlled trials and crossover studies

Study authors, year	Funding	EPHPP							Study period	Protocol registration date
		Selection bias	Study design	Confounders	Blinding	Data collection	Drop outs	Overall		
Ludicke et al., 2017a [5]**	Tob	2	1	1	2	1	1	Strong	06–07/2012	01/2013**
Ludicke et al., 2016 [6]*	Tob	2	1	1	3	1	1	Moderate	11/2008–02/2009	12/2008*
Picavet et al., 2016 [2]**	Tob	2	2	3	3	1	1	Weak	05–06/2012	01/2013**
Lopez et al., 2016 [7]	Indep	2	2	3	3	1	3	Weak	Not reported	Not registered
Haziza et al., 2016a [8]**	Tob	2	1	1	2	1	1	Strong	07/2013	10/2013**
Haziza et al., 2016b [9]**	Tob	3	1	1	2	1	1	Moderate	07–09/2013	10/2013**
Ludicke et al., 2017b [10]**	Tob	3	1	1	1	1	1	Moderate	07/2013	10/2013**
Ludicke et al., 2017c [11]**	Tob	3	1	1	1	1	1	Moderate	07/2013	10/2013**
Brossard et al., 2017 [3]*	Tob	3	2	1	3	1	1	Weak	07–11/2013	10/2013*
Gee et al., 2017 [12]	Tob	2	2	3	3	1	3	Weak	Not reported	Not reported
Yuki et al., 2017 [4]	Tob	2	2	3	3	1	1	Weak	Not reported	Not reported

Note: 1 - strong, 2 - moderate, 3 - weak. Tob – tobacco industry-funded research, indep – independently-funded research.
* Study protocol has been registered while the study was ongoing
** Study protocol has been registered when the study had been finished

Appendices

Table A1 Search strategies and outcomes for all databases 5

Table A2 Studies and findings on heat not burn sidestream, mainstream and secondhand emissions 6

Table A3 Findings of the studies on heat not burn use by human participants 34

Table A4 Findings of epidemiology studies on heat not burn use 54

Table A1 Search strategies and outcomes for all databases

Database	Search strategy	Outcome on 13th July 2017
**Medline**<br>Ovid MEDLINE(R) In-Process & Other Non-Indexed Citations and Ovid MEDLINE(R) 1946 to Present	1. "heat not burn".mp.<br>2. "tobacco heating system".mp.<br>3. (heat* adj3 tobacco).mp.<br>4. IQOS.mp.<br>5. Ploom.mp.<br>6. Heets.mp. OR glo.mp.<br>7. 1 or 2 or 3 or 4 or 5 or 6<br>8. limit 7 to yr="2010 -Current"	77 references exported
**Embase**<br>Embase 1974 to 2017 Week 28	1. "heat not burn".mp.<br>2. "tobacco heating system".mp.<br>3. (heat* adj3 tobacco).mp.<br>4. IQOS.mp.<br>5. Ploom.mp.<br>6. Heets.mp. OR glo.mp.<br>7. 1 or 2 or 3 or 4 or 5 or 6<br>8. limit 7 to yr="2010 -Current"	104 references exported
**PsycINFO**<br>PsycINFO 1806 to July Week 1 2017	1. "heat not burn".mp.<br>2. "tobacco heating system".mp.<br>3. (heat* adj3 tobacco).mp.<br>4. IQOS.mp.<br>5. Ploom.mp.<br>6. Heets.mp. OR glo.mp.<br>7. 1 or 2 or 3 or 4 or 5 or 6<br>8. limit 7 to yr="2010 -Current"	12 references exported
**ProQuest Social Sciences Premium Collection**	“Heat not burn” OR<br>"Tobacco heating system"OR<br>(heat* hadj3 tobacco) OR<br>IQOS OR<br>Ploom OR<br>Heets OR<br>glo<br>Limited to: after 01/01/2010 AND Peer reviewed	20 references exported
**Scopus**	(ALL ("Heat not burn") OR	492 references exported

	ALL ("Tobacco heating system") OR<br>ALL (heat??? W/3 tobacco) OR<br>ALL ("IQOS" OR "Ploom " OR "Heets" OR "glo")) AND PUBYEAR > 2009	
**Web of Science**<br>Web of Science Core Collection	TOPIC: ("Heat not burn") OR<br>TOPIC: ("Tobacco heating system") OR<br>TOPIC: (heat* Near/3 tobacco) OR<br>TOPIC: ("IQOS" OR "Ploom" OR "Heets" OR "Heatsticks" OR "glo")<br>Timespan: 2010-2017.<br>Indexes: SCI-EXPANDED, SSCI, A&HCI, CPCI-S, CPCI-SSH, ESCI.	138 references exported

Table A2 Studies and findings on heat not burn sidestream, mainstream and secondhand emissions

**Mainstream emissions produced using machine smoking**	
**Authors, study year**	**Auer et al., 2017 [13]**
**Funder/Affiliations**	**Affiliations:**<br>• Institute of Primary Health Care (BIHAM), University of Bern, Bern, Switzerland<br>• Department of Ambulatory Care and Community Medicine, University of Lausanne, Lausanne, Switzerland<br>• Institute for Work and Health, University of Lausanne and Geneva, Lausanne, Switzerland
**Primary aim**	To compare levels of HPHC in mainstream IQOS emissions with those in mainstream cigarette smoke
**Products used**	• IQOS with regular tobacco sticks<br>• Cigarette (Lucky Strike Blue Lights)
**Methods**	**Design:** Laboratory comparison study using smoking machines<br>**Study time and setting:** not reported<br>**Method description:** For tested products mainstream smoke and aerosol was produced following the International Organization for Standardization (ISO) machine smoking regime (35 ml puff volume at 2 puffs per minute, for 5–6 minutes or a mean of 14 puffs). Polycyclic aromatic hydrocarbons generated by reference cigarettes were not analysed and for comparison the mean values in the 35 best-selling cigarettes brands in the United States are used [14].
**Participants**	Not reported
**Interventions/Exposure**	‘We trapped polycyclic aromatic hydrocarbons from IQOS cigarette smoke in a glass filter (Whatman 37 mmØGF/B) mounted in line with an XAD2 cartridge. For each sampling, 10 IQOS cigarettes were smoked. Each sampling support was desorbed in 10mLof acetonitrile and sonicated for 1 hour. The eluate was evaporated in a vacuum concentrator (Speed Vac SC-200, ThermoFisher

	Scientific) set with 30 millibars and 27g until the residue was almost dry to prevent evaporation of the most volatile polycyclic aromatic hydrocarbons. The residue was filtered with polytetrafluoroethylene membrane (Acrodisc CR 13 mm, 0.45 µm, Pall Life Sciences) before it was analysed with a high-performance liquid chromatography device (Ultimate 3000, ThermoFisher Scientific) equipped with a fluorescence detector (FLD- 3000RS), UV detector (VWD-3000), and a separation column Nucleodur EC 150 × 3mm C18 3 µm (Macherey-Nagel) under isocratic conditions (1.2mL min^{-1}).We injected 2 µL into the high-performance liquid chromatography chain; methanol/ water (70/30) with acetonitrile was the eluent solvent at an initial ratio of 100% to 0% (4 minutes) and a linear gradient up to100% acetonitrile (12 minutes)' [13]
**Outcome/Key findings**	HPHC in mainstream IQOS aerosol compared with reference cigarette smoke (table below)

HPHC in mainstream IQOS aerosol compared with reference cigarette smoke

HPHC	Mean (SD)	% ratio HnB aerosol : cigarette smoke
**Volatile organic compounds**		
Acetaldehyde (µg/stick)	133 (35)	22%
Acetone (µg/stick)	12 (12.9)	13%
Acroleine (µg/stick)	0.9 (0.6)	82%
Benzaldehyde (µg/stick)	1.2 (1.4)	50%
Crotonaldehyde (µg/stick)	0.7 (0.9)	4%
Formaldehyde (µg/stick)	3.2 (2.7)	74%
Isovaleraldehyde (µg/stick)	3.5 (3.1)	41%
Propionaldehyde (µg/stick)	7.8 (4.3)	26%
**Polycyclic aromatic hydrocarbons**		
Naphthalene (ng/stick)	1.6 (0.5)	0.1%
Acenaphthylene (ng/stick)	1.9 (0.6)	0.8%
Acenaphthene (ng/stick)	145 (54)	295%
Fluorene (ng/stick)	1.5 (0.6)	0.4%
Anthracene (ng/stick)	0.3 (0.1)	0.2%
Phenanthrene (ng/stick)	2.0 (0.2)	0.7%
Fluoranthene (ng/stick)	7.3 (1.1)	6%
Pyrene (ng/stick)	6.4 (1.1)	7%
Benz[a]anthracene (ng/stick)	1.8 (0.4)	6%
Chrysene (ng/stick)	1.5 (0.3)	3%
Benzo[b]fluoranthene (ng/stick)	0.5 (0.2)	2%
Benzo[k]fluoranthene (ng/stick)	0.4 (0.2)	9%
Benzo[a]pyrene (ng/stick)	0.8 (0.1)	4%
**Other inorganics**		
Carbon dioxide (ppm)	3057 (532)	Not analysed
Carbon monoxide (ppm)	328 (76)	Not analysed
Nitric oxide (ppm)	5.5 (1.5)	6%

	Nicotine (mg/stick)	0.301 (0.213)	84%

**Conclusions**	• 'The emissions released by IQOS contain elements from pyrolysis and thermogenic degradation that are the same harmful constituents of conventional tobacco cigarette smoke' [13].
**Authors, study year**	**Farsalinos et al., 2017 [15]**
**Funder/Affiliations**	**Affiliations:**<br>• Department of Cardiology, Onassis Cardiac Surgery Center, Kallithea, Greece<br>• Department of Pharmacy, University of Patras, Rio-Patras, Greece<br>• Skylab-Med Laboratories of Applied Industrial Research and Analysis S.A., Marousi, Greece<br>• Declaration of interests: Two studies by Farsalinos were funded by the non-profit association American E-liquid Manufacturing Standards Association in 2013 and one study was funded by the non-profit Tennessee Smoke-Free Association in 2015.
**Primary aim**	To compare levels of nicotine in mainstream IQOS emissions from regular and menthol tobacco sticks with nicotine in different type of e-cigarettes aerosol and in mainstream cigarette smoke
**Products used**	• IQOS (purchased in Milan, Italy) and tobacco sticks (regular and menthol, purchased in Milan)<br>• Marlboro Regular cigarette (purchased in Athens, Greece)<br>• First generation cigalike e-cigarette (Vapour 2 cigs, Prague, Czech Republic)<br>• Second generation pen-style e-cigarette (Epsilon, Nobacco, Athens, Greece): an eGo battery (1100 mAh) and a tank-type bottom coil atomizer<br>• Third generation tank style e-cigarette: battery device (EVIC VTC Mini, Joyetech, Shenzhen, China), tank-type atomizer (Nautilus Mini, Aspire, Shenzhen, China)<br>• For all e-cigarettes the same custom made liquid was used: 45% propylene glycol, 45% glycerol, 8% water and 2% nicotine
**Methods**	**Design:** Laboratory comparison study using smoking machines<br>**Study time and setting:** 2015, Athens, Greece<br>**Method description:** For tested products mainstream smoke and aerosol was produced using Health Canada Intense (HCI) machine-smoking regime (55 ml puff volume, 27.5 ml/s puff flow rate, 2 s puff duration, 30 s inter-puff interval). For e-cigarettes and HnB, an additional puffing regime (55 ml puff volume, 13.75 ml/s puff flow rate, 4 s puff, and 30 s inter-puff interval) was used for comparison.
**Participants**	Not reported
**Interventions/Exposure**	'The method for quantification of nicotine was based on the WHO official method SOP 04.<br>Unused tobacco sticks from HnB were examined for the levels of nicotine per weight of tobacco. After careful removal of paper and filter from the tobacco stick, the tobacco was weighted. Subsequently, 200 mg of tobacco was mixed with 1 mL of 2% quinoline solution in n-hexane (used as internal standard), 4 mL distilled water, and 2 mL NaOH. The solution was allowed to rest for 15 minutes. Then, it was introduced to a round bottom flask and 200 mL n-hexane was added. The solution was stirred strongly using a magnetic stirrer for 1 hour and then it was transferred in a separator funnel for the separation of two layers. From the supernatant layer, 200 µL was further diluted with n-hexane to a final volume of 10 mL. The lower, aqueous layer was re-extracted with 200 mL

n-hexane. From the supernatant layer of this second extraction, 200 µL were further diluted with n-hexane to a final volume of 10 mL. Finally, both extracts were analyzed with GC-NPD for the nicotine content, and the nicotine concentration was calculated as mg/g tobacco' [15]

Outcome/Key findings

Levels of nicotine in mainstream aerosol of IQOS and tested e-cigarettes compared with reference cigarette smoke

Tested product	Nicotine in tobacco (mg/g)	Nicotine in aerosol, mg (% ratio vs ref cigarette)	
		2 seconds HCI regime	4 seconds HCI regime
Reference cigarette		1.99 ± 0.20 (reference)	
Regular IQOS	15.2 ± 1.1	1.40 ± 0.16 (70.4%)	1.41 ± 0.08 (70.9%)
Menthol IQOS	15.6 ± 1.7	1.38 ± 0.11 (69.3%)	1.43 ± 0.13 (71.9%)
Cigalike e-cigarette		0.46 ± 0.06 (23.1%)	0.86 ± 0.08 (43.2%)
Pen-style e-cigarette		0.51 ± 0.05 (25.6%)	1.73 ± 0.09 (86.9%)
Tank style e-cigarette		0.82 ± 0.06 (41.2%)	1.84 ± 0.11 (92.5%)

Conclusions

- IQOS delivered less nicotine to mainstream aerosol than tobacco cigarette did. Under 2 seconds HCI regime, IQOS nicotine delivery was higher compared with all tested e-cigarettes, but lower than pen-style and tank style e-cigarettes' at 4 seconds HCI regime

Authors, study year

Bekki et al., 2017 [16]

Funder/Affiliations

Funded by:

- The Health and Labour Science Research Grants from Ministry of Health
- Labour and Welfare of the Japanese Government
- The practical research project for life-style related diseases including cardiovascular diseases and diabetes mellitus from Japan Agency for Medical Research and Development, AMED.

Affiliations:

- Department of Environmental Health, National Institute of Public Health. Minami, Wako-shi, Saitama 351-0197, Japan

Primary aim

To compare levels of nicotine and HPHC in mainstream IQOS emissions from regular and menthol tobacco sticks with those in mainstream cigarette smoke

Products used

- Reference cigarettes 3R4F (high yield) and 1R5F (low yield)
- IQOS with regular and menthol tobacco sticks

Methods

Design: Laboratory comparison study using smoking machines

Study time and setting: Japan, time not reported

	**Method description:** For tested products mainstream smoke and aerosol was produced using Health Canada Intense (HCI) machine smoking regime (55 ml puff volume, 2 s puff duration, 30 s puff interval, and 100% blocking of the filter ventilation holes).
**Participants**	Not reported
**Interventions/Exposure**	Each sampling was performed by 3 cigarettes and tobacco sticks. A cigarette was puffed 9 times, and one tobacco stick was puffed 11 times.
**Outcome/Key findings**	• IQOS regular tobacco sticks had 15.7 mg of nicotine and menthol tobacco sticks had 17.1 mg of nicotine per gram of tobacco. These estimates were similar to nicotine in reference cigarettes: 19.7 mg/g in 3R4F and 15.9 mg/g in 1R5F.<br>• IQOS showed more effective transfer rate of nicotine from tobacco sticks to mainstream aerosol than reference cigarettes: 23.4% (regular) and 23.5% (menthol) compared with 11.3% (3R4F) and 11.5% (1R5F).<br><br>HPHC in mainstream IQOS aerosol of regular and menthol tobacco sticks and in smoke of reference cigarettes (see table below)

HPHC in mainstream IQOS aerosol of regular and menthol tobacco sticks and in smoke of reference cigarettes

	IQOS regular	IQOS menthol	3R4F cigarette	1R5F cigarette
**HPHC**	**Mean ± SD**	**Mean ± SD**	**Mean ± SD**	**Mean ± SD**
Total particulate matter (mg/stick)	44.0 ± 11.4	49.9 ± 8.6	36.9 ± 1.9	28.9 ± 2.3
Water (mg/stick)	33.1 ±10.2	35.3 ± 8.3	10.1 ± 0.9	8.8 ± 1.1
Tar (mg/stick)	9.8 ± 3.0	13.4 ± 2.2	25.2 ± 1.5	19.2 ± 1.3
Nicotine (mg/stick)	1.1 ± 0.1	1.2 ± 0.1	1.7 ± 0.1	1.0 ± 0.1
Carbon monoxide (mg/stick)	0.44 ± 0.04	0.43 ± 0.04	33.0 ± 1.8	29.7 ± 1.7
**Tobacco-specific nitrosamines**				
NAB (ng/stick)	4.5 ± 0.5	5.5 ± 0.6	30.4 ± 2.0	26.2 ± 0.5
NAT (ng/stick)	34.0 ± 3.1	37.2 ± 3.9	246.4 ± 16.9	183.1 ± 6.0
NNN (ng/stick)	19.2 ± 2.1	24.9 ± 3.5	311.1 ± 24.3	240.7 ± 6.6
NNK (ng/stick)	12.3 ± 1.5	13.8 ± 2.6	250.4 ± 13.7	107.0 ± 5.0
Total (ng/stick)	70.0 ± 7.2	81.4 ± 10.4	838.2 ± 53.7	557.1 ± 15.7

**Authors' conclusions**	• Although at lower levels than in mainstream smoke of reference cigarettes, IQOS mainstream emissions include detectable levels of HPHC and particulate matter
**Authors, study year**	**Schaller et al., 2016** [17]
**Funder/Affiliations**	**Affiliations:** Philip Morris International R&D, Philip Morris Products S.A., Quai Jeanrenaud 5, 2000 Neuchatel, Switzerland
**Primary aim**	To compare levels of HPHC in mainstream IQOS emissions with those in mainstream cigarette smoke

**Products used**	• Reference cigarette 3R4F<br>• THS 2.2 (IQOS) used with two regular and two menthol tobacco sticks variants
**Methods**	**Design:** Laboratory comparison study using smoking machines<br>**Study time and setting:** Switzerland, time not reported<br>**Measures:** For tested products mainstream smoke and aerosol was produced using Health Canada Intense (HCI) machine smoking regime
**Participants**	Not reported
**Interventions/Exposure**	'The reference cigarette 3R4F was smoked to a butt length of 35 mm using a bell-shaped puff profile and 100% blocking of ventilation holes. THS2.2 Regular and Menthol tobacco sticks were 'smoked' using a bell-shaped puff profile to a defined puff count of 12 puffs' [17]
**Outcome/Key findings**	Yields of HPHC in the aerosol of THS regular and menthol tobacco sticks and in comparison to reference cigarette

	THS 2.2 regular*	THS 2.2 menthol*	3R4F reference cigarette
**HPHC**	**Mean ± 95% CI**	**Mean ± 95% CI**	**Mean ± 95% CI**
**Gases**			
Ammonia (µg/stick)	14.2 ± 1.1	13.8 ± 0.7	39.3 ± 3.2
Nitric oxide (µg/stick)	16.8 ± 2.3	12.3 ± 1.7	491 ± 38
Nitrogen oxides (µg/stick)	17.3 ± 2.6	12.6 ± 1.7	537 ± 43
Hydrogen cyanide (µg/stick)	4.81 ± 0.35	5.14 ± 0.70	493 ± 78
**Carbonyls**			
Butyraldehyde (µg/stick)	26.1 ± 2.3	26.7 ± 2	88.4 ± 10.7
Acetaldehyde (µg/stick)	219 ± 31	205 ± 12	1555 ± 184
Propionaldehyde (µg/stick)	14.5 ± 2.4	13.9 ± 0.7	125 ± 16
Formaldehyde (µg/stick)	5.53 ± 0.69	4.55 ± 0.25	56.5 ± 12.1
Acrolein (µg/stick)	11.30 ± 2.36	9.15 ± 0.43	154 ± 20
Crotonaldehyde (µg/stick)	4.14 ± 0.23	3.24 ± 0.21	68.8 ± 14.4
Acetone (µg/stick)	40.7 ± 6.2	39.4 ± 2.3	736 ± 129
Methyl ethyl ketone (µg/stick)	7.18 ± 1.19	6.93 ± 0.64	187 ± 30
**Volatile and semi-volatile organic compounds**			
Pyridine (µg/stick)	7.54 ± 0.26	7.21 ± 0.25	36.1 ± 2.2
Styrene (µg/stick)	0.608 ± 0.058	0.561 ± 0.033	24.5 ± 1.2
Toluene(µg/stick)	2.59 ± 0.43	2.39 ± 0.16	188 ± 11
Acrylonitrile (µg/stick)	0.258 ± 0.041	0.220 ± 0.014	31.9 ± 1.8
1,3-Butadiene (µg/stick)	0.294 ± 0.042	0.265 ± 0.024	63.8 ± 3.5
Benzene (µg/stick)	0.649 ± 0.074	0.640 ± 0.040	97.6 ± 4.7

	Isoprene (µg/stick)	2.35 ± 0.39	2.11 ± 0.18	798 ± 49
	Quinoline (µg/stick)	<0.012	<0.012	0.513 ± 0.023
	**Polycyclic aromatic hydrocarbons**			
	Benzo[a]pyrene (ng/stick)	<1.00	1.29 ± 0.10	14.2 ± 0.3
	Benz [a]anthracene (ng/stick)	1.45 ± 0.14	2.49 ± 0.17	28.0 ± 0.6
	Pyrene (ng/stick)	<5.00	9.06 ± 0.68	87.3 ± 2.5
	Dibenz [a,h]anthracene (ng/stick)	<0.100	<0.100	1.70 ± 0.11
	**Phenols and acid derivatives**			
	Acrylamide (µg/stick)	1.73 ± 0.12	1.91 ± 0.16	4.8 ± 0.3
	Acetamide (µg/stick)	4.02 ± 0.18	4.30 ± 0.24	13.9 ± 0.5
	Catechol (µg/stick)	16.3 ± 1.5	17.1 ± 1.1	91.4 ± 5.6
	Phenol (µg/stick)	1.16 ± 0.12	1.60 ± 0.4	13.6 ± 0.9
	Hydroquinone (µg/stick)	8.10 ± 0.48	8.98 ± 1.02	83.1 ± 5.5
	o-Cresol (µg/stick)	0.069 ± 0.008	0.095 ± 0.025	4.47 ± 0.16
	m-Cresol (µg/stick)	0.029 ± 0.004	0.033 ± 0.006	3.03 ± 0.08
	p-Cresol (µg/stick)	0.072 ± 0.008	0.083 ± 0.010	9.17 ± 0.44
	**Aromatic amines**			
	o-Toluidine (ng/stick)	1.260 ± 0.187	0.777 ± 0.287	85.5 ± 2.7
	1-Aminonaphthalene (ng/stick)	0.077	0.086	20.8 ± 1.3
	2-Aminonaphthalene (ng/stick)	0.046 ± 0.008	<0.035	11.0 ± 0.6
	3-Aminobiphenyl (ng/stick)	<0.032	0.032	3.77 ± 0.47
	4-Aminobiphenylx (ng/stick)	<0.051	<0.051	3.26 ± 0.12
	**Tobacco-specific nitrosamines**			
	NAB (ng/stick)	<3.15	<3.15	33.7 ± 8.5
	NAT (ng/stick)	20.5 ± 0.5	19.7 ± 3.6	318 ± 74
	NNN (ng/stick)	17.2 ± 1.25	13.7 ± 1.21	309 ± 41
	NNK (ng/stick)	6.7 ± 0.6	5.9 ± 0.4	266 ± 15
	**Elements**			
	Selenium (ng/stick)	<0.550	0.780	1.62 ± 0.32
	Mercury (ng/stick)	1.17 ± 0.05	1.34 ± 0.18	4.80 ± 0.13
	Arsenic (ng/stick)	<1.13	<1.13	8.51 ± 0.34
	Lead (ng/stick)	<3.35	<3.35	37.0 ± 0.7
	Cadmium (ng/stick)	<0.350	<0.350	161 ±4
	Nickel (ng/stick)	<0.55	<0.55	<0.55
	Chromium	<0.55	<0.55	<0.55
	**Epoxides and vinyl chloride**			

	<table><tr><td>Propylene oxide (µg/stick)</td><td>0.148 ± 0.018</td><td>0.149 ± 0.017</td><td>1.32 ± 0.12</td></tr><tr><td>Vinyl chloride (ng/stick)</td><td><3.54</td><td><3.54</td><td>96.7 ± 2.0</td></tr><tr><td>Ethylene oxide (µg/stick)</td><td>0.201 ± 0.014</td><td>0.202 ± 0.013</td><td>29.4 ± 2.0</td></tr><tr><td>**Other compounds**</td><td></td><td></td><td></td></tr><tr><td>Water (mg/stick)</td><td>36.5 ± 3.1</td><td>29.7 ± 3.6</td><td>15.8 ± 2.9</td></tr><tr><td>Glycerin (mg/stick)</td><td>4.63 ± 0.83</td><td>3.94 ± 0.87</td><td>2.42 ± 0.14</td></tr><tr><td>Total particulate matter (mg/stick)</td><td>48.2 ± 2.4</td><td>43.5 ± 1.5</td><td>49.0 ± 4.8</td></tr><tr><td>Nicotine (mg/stick)</td><td>1.32 ± 0.16</td><td>1.21 ± 0.09</td><td>1.89 ± 0.16</td></tr><tr><td>Nicotine-free dry particulate matter (mg/stick)</td><td>10.3 ± 0.9</td><td>12.6 ± 2.2</td><td>31.2 ± 1.8</td></tr><tr><td>Nitrobenzene (ng/stick)</td><td><0.188</td><td>0.335 ± 0.164</td><td>8.62 ± 1.10</td></tr><tr><td>Carbon monoxide (mg/stick)</td><td>0.531 ± 0.068</td><td>0.594 ± 0.110</td><td>32.8 ± 2.4</td></tr><tr><td>Menthol (mg/stick)</td><td>N/A</td><td>2.62 ± 0.1</td><td>N/A</td></tr></table>* only results of FR1 and FR1 M versions of tobacco sticks provided
**Findings overview**	• There are significantly lower concentrations of HPHCs in the mainstream aerosol of THS2.2 compared with the mainstream smoke of the 3R4F reference cigarette<br>• The reductions in the concentrations of most HPHCs in the THS2.2 aerosol were greater than 90% when compared with 3R4F<br>• Tobacco combustion of tobacco did not appear when using the THS2.2 with more intense puffing regimens than the HCI conditions
**Authors, study year**	**Schaller et al., 2016** [18]
**Funder/Affiliations**	**Affiliations:** Philip Morris International R&D, Philip Morris Products S.A., Quai Jeanrenaud 5, 2000 Neuchatel, Switzerland
**Primary aim**	To compare levels of HPHC in mainstream IQOS emissions from regular and menthol tobacco sticks with those in mainstream cigarette smoke
**Products used**	• Reference cigarette 3R4F<br>• THS 2.2 (IQOS) used with 43 experimental tobacco blends
**Methods**	**Design:** Laboratory comparison study using smoking machines<br>**Study time and setting:** Switzerland, time not reported<br>**Measures:** For tested products mainstream smoke and aerosol was produced using Health Canada Intense (HCI) machine smoking regime
**Participants**	Not reported
**Interventions/Exposure**	Each tobacco stick and 3R4F reference cigarettes were conditioned following the ISO 3402 protocol, then mainstream aerosol was produced from the stick using the Health Canada intense machine-smoking regimen.
**Outcome/Key findings**	Yields of HPHC in the aerosol of THS control tobacco blend and in comparison to reference cigarette

HPHC	THS2.2 control tobacco blend	3R4F reference cigarette
	**Mean ± 95% CI**	**Mean**
**Gases**		
Ammonia (µg/stick)	12.0 ± 5.2	31.2
Nitric oxide (µg/stick)	13.0 ± 2.4	510
Nitrogen oxides (µg/stick)	13.8 ± 2.4	571
Carbon monoxide (mg/stick)	0.446±0.246	30.6
**Carbonyls**		
Butyraldehyde (µg/stick)	24.0 ± 8.1	83.5
Acetaldehyde (µg/stick)	211±60	1694
Propionaldehyde (µg/stick)	14.6 ± 10.5	122
Formaldehyde (µg/stick)	10.16 ± 10.08	88.9
Acrolein (µg/stick)	10.96 ± 5.16	161
Crotonaldehyde (µg/stick)	<3.29	51.7
Acetone (µg/stick)	35±11.3	685
Methyl ethyl ketone (µg/stick)	7.95 ± 6.65	183
**Volatile and semi-volatile organic compounds**		
Pyridine (µg/stick)	8.27 ± 3.06	31.5
Styrene (µg/stick)	1.067 ± 2.528	16.5
Quinoline (µg/stick)	<0.011	0.44
Resorcinol (µg/stick)	<0.055	1.75
Toluene (µg/stick)	2.49 ± 1.69	137
Acrylonitrile (µg/stick)	0.177 ± 0.173	24.0
1,3-Butadiene (µg/stick)	0.272 ± 0.101	97.0
Benzene (µg/stick)	0.700 ± 0.540	81.1
Isoprene (µg/stick)	2.14 ± 0.44	884
**Polycyclic aromatic hydrocarbons**		
Dibenz [a,h]anthracene (ng/stick)	<0.413	0.79
Benz [a]anthracene (ng/stick)	2.64 ± 2.46	27.2
Pyrene (ng/stick)	8.01 ± 4.80	79.3
Benzo[a]pyrene (ng/stick)	1.02±0.69	15.0
**Phenols and acid derivatives**		
Acrylamide (µg/stick)	1.85 ± 1.33	4.5
Acetamide (µg/stick)	3.31 ± 1.69	13.0
Catechol (µg/stick)	13.2 ± 5.6	89.6

	Phenol (µg/stick)	1.12 ± 0.52	13.9
	Hydroquinone (µg/stick)	6.23 ± 2.46	88.3
	o-Cresol (µg/stick)	0.052 ± 0.036	4.11
	m-Cresol (µg/stick)	0.031 ± 0.036	3.61
	p-Cresol (µg/stick)	0.068 ± 0.097	8.86
	**Aromatic amines**		
	o-Toluidine (ng/stick)	1.616 ± 0.883	103.9
	1-Aminonaphthalene (ng/stick)	0.069±0.077	21.2
	2-Aminonaphthalene (ng/stick)	0.045±0.06	16.2
	3-Aminobiphenyl (ng/stick)	0.012±0.012	4.09
	4-Aminobiphenyl (ng/stick)	0.012±0.012	2.77
	**Tobacco-specific nitrosamines**		
	NAB (ng/stick)	3.01 ± 1.13	30.3
	NAT (ng/stick)	17.5 ± 9.3	269
	NNN (ng/stick)	14.2 ± 5.9	284
	NNK (ng/stick)	7.1 ± 2.8	261
	**Elements**		
	Selenium (ng/stick)	<0.83	1.49
	Mercury (ng/stick)	1.25 ± 0.48	4.67
	Arsenic (ng/stick)	<1.20	7.99
	Lead (ng/stick)	<1.62	31.9
	Cadmium (ng/stick)	<0.280	94
	Nickel (ng/stick)	<53	<53
	Chromium (ng/stick)	<11	<11
	**Epoxides and vinyl chloride**		
	Propylene oxide (µg/stick)	0.078 ± 0.021	1.11
	Vinyl chloride (ng/stick)	<2.19	100.8
	Ethylene oxide (µg/stick)	0.199 ± 0.141	24.1
	**Other compounds**		
	Glycerin (mg/stick)	4.63±1.01	2.28
	Water (mg/stick)	32.1±6.5	15.8
	Total particulate matter (mg/stick)	54.7±3.2	44.7
	Nicotine-free dry particulate matter (mg/stick)	21.2±8.5	26.8
	Nicotine (mg/stick)	1.38±0.2	1.88
	Hydrogen cyanide (µg/stick)	<4.37	364

	Nitrobenzene (ng/stick) <37.84 <37.84
**Findings overview**	• The mainstream aerosols produced by 43 different experimental tobacco plug blends in the THS2.2 contained significantly lower concentrations of HPHCs than found in the mainstream smoke of reference cigarette 3R4F. The tobacco blend composition in tobacco sticks had only a minimal impact on the HPHC levels in mainstream aerosols.<br>• Ammonia, tobacco specific nitrosamines, nitrogen oxides, poly-aromatic hydrocarbons, acrylamide and acetamide concentrations in the THS2.2 mainstream aerosols showed significant variability across the 43 experimental tobacco sticks blends
**Authors, study year**	**Protano et al., 2016 [19]**
**Funder/Affiliations**	**Affiliations:**<br>• Department of Public Health and Infectious Diseases, Sapienza University of Rome, Italy<br>• Department of Technological Innovations, INAIL, Rome, Italy<br>• Centre of Occupational Medicine, Sapienza University of Rome, Italy
**Primary aim**	To compare levels of secondhand smoke and emissions between tested tobacco and nicotine products
**Products used**	• IQOS tobacco stick<br>• Pall Mall San Francisco cigarette<br>• Hand-rolled cigarette (Golden Virginia® tobacco hand- rolled with a Rizla® Blue Regular Rolling Paper)<br>• Pen-style e-cigarette (Smooke® E-SMART (L)) filled with Smooke® Light e-liquid (9 mg/ml nicotine)
**Methods**	**Design:** Laboratory comparison study using smoking volunteers<br>**Study time and setting:** 2015<br>**Method description:** Submicron particles were measured using a Fast Mobility Particle Sizer spectrometer (FMPS 3091, TSI Inc.) in a room of 52.7 m^3 with a door and a window (0.67 air changes/h in the room). The FMPS 3091 measures particle size distribution in the range 5.6-560 nm using the electrical mobility technique, with a 1-s time resolution.
**Participants**	Two researchers of Sapienza University in Rome: 53-year old male and 37-year old female, both smokers at the time of the study
**Interventions/Exposure**	To simulate passive exposure of the subjects, the air sampler was placed 2 meters away from the smoker and at 1.5 meters above the floor. The door and the window were opened before each experiment to reach a steady submicron particles concentration; then, the door and the window were kept closed until the end of each experiment.<br>For each experiment, lasting one hour from the cigarette or device ignition, the submicron particles deposition dose was modelled in the human respiratory tree with the Multiple-Path Particle Dosimetry model (MPPD v2.1, ARA 2009). Each experiment was run in triplicate; arithmetic mean values were calculated for each 1-s time measurement and used for data comparison.
**Outcome/Key findings**	**Number (%) of total deposited submicron particles in different respiratory regions for a normal adult male breathing through nose in rest condition**

Respiratory region	Product			
	Cigarette	Hand-rolled cigarette	IQOS	E-cigarette
Head	2.87 x 10^9 (18%)	2.24 x 10^9 (17%)	0.665 x 10^9 (17%)	0.834 x 10^9 (20%)

	Tracheo-bronchial: 4.85×10^9 (30%); 3.91×10^9 (30%); 1.18×10^9 (30%); 1.33×10^9 (32%)<br>Alveolar: 8.39×10^9 (52%); 6.89×10^9 (53%); 2.15×10^9 (53%); 1.91×10^9 (48%)<br>Total: 16.11×10^9; 13.04×10^9; 4.00×10^9; 4.07×10^9<br>• During smoking, submicronic particles released by traditional and hand-rolled cigarettes and deposited in the respiratory tract of a passively exposed subject are four-times higher than those released by e-cigarettes and HnB devices<br>• After smoking, submicronic particles generated by traditional and hand-rolled cigarettes remain high until the end of the experiment (about six-times higher than background) while, for e-cigarettes and HnB devices, submicronic particles' values return immediately to similar to background levels
**Conclusions**	• Exposure to submicronic particles generated by e-cigarette and HnB devices occurs during use period and becomes negligible when the devices are turned off<br>• Exposure to submicronic particles when e-cigarette and HnB are used still occur and can be inhaled and reach alveolar region of a passive smoker
**Authors, study year**	**Ruprecht et al., 2017 [20]**
**Funder/Affiliations**	**Funded by:** Fondazione IRCCS, Istituto Nazionale dei Tumori, Milan, Italy and by the University of Southern California<br>**Affiliations:**<br>• Fondazione IRCCS Istituto Tumori, Milan, Italy;<br>• Department of Civil and Environmental Engineering, University of Southern California, California, USA;<br>• Cardiopulmonary Rehabilitation Unit, Azienda Sociosanitaria Territoriale Lariana, Sant'Antonio Abate Hospital, Cantu, Italy;<br>• Aerosol and Air Pollution Research Group, School of Energy and Environment, City University of Hong Kong, Hong Kong;<br>• Environmental Chemistry and Technology Program, University of Wisconsin-Madison, Madison, Wisconsin, USA;<br>• Repace Associates Inc., Bowie, Maryland, USA
**Primary aim**	To compare levels of secondhand emissions by IQOS, e-cigarette, and cigarette in an indoor environment
**Products used**	• IQOS<br>• Tank-style e-cigarettes: "Elips Serie C," Tank System (Ovale Europe Srl), refilled with 16 mg/ml nicotine cartridges<br>• Cigarettes
**Methods**	**Design:** Laboratory comparison study using smoking volunteers<br>**Study time and setting:** not reported<br>**Measures:** Black Carbon (BC) was measured at two wavelengths (880 nm and 370 nm), using an Aethalometer (model AE31, Magee Scientific) and reported in ng/m^3. Particulate matter number concentration was monitored using a Met One Instruments particle counter operating with two channels (dp > 1.0 mm and dp > 0.3 mm). Additionally, submicron particles (10–1000 nm in size) were measured using a condensation particle counter (CPC model 3007, TSI Inc., Shoreview, MN, USA). Particle mass (PM) concentration (in mg/m^3) was measured at three size ranges, namely PM_1, $PM_{2.5}$, and PM_{10}, using a Met One PM mass monitor (model Aerocet

	531). The instrument was gravimetrically pre-calibrated by parallel comparison with the model BAM-1020 (Met One Instruments Inc.) with US Environmental Protection Agency (EPA) equivalence certificate designation N.° EQPM-0798-122.<br>Trace elements, metals, and particle-phase organic compounds were measured by time-integrated collections of PM samples, followed by offline extraction and chemical analysis. Total Suspended Particles were collected on quartz and Teflon filters (2 mm pore size, Whatman International Ltd., Midlestone, UK) loaded on four Sioutas Personal Cascade Impactor Samplers (Sioutas™ PCIS, SKC Inc., Eighty Four, PA, USA) each operating at 10 l/min, outdoors and indoors. To measure the metals and trace element concentrations, Teflon filters were digested in an acid mixture (comprised of nitric acid, hydrochloric acid, and hydrogen peroxide), inside of a microwave-assisted Teflon-made digestion bomb (Milestone ETHOSC+), and subsequently analysed using a high-resolution magnetic sector inductively coupled plasma mass spectrometry (ICP-MS; Thermo-Finnigan Element 2). To measure the concentration of individual organic species (including but not limited to alkanes, polycyclic aromatic hydrocarbons, and organic acids), quartz filters were extracted in a 1: 1 solution of dichloromethane and acetone, using Soxhlets, followed by volume reduction using rotary evaporation under high purity nitrogen and derivatization of carboxylic acids with diazomethane. The substrates were then analysed by gas chromatography mass spectrometry (GC-MS) method (GC-6980, quadruple MS-5973, Agilent Technologies). In addition to the particle phase measurements, gas-phase aldehydes were also collected on silica vials activated with 2,4-Dinitrophenylhydrazine (DNPH) and analysed according to the EPA method TO-11A -1999 (method TO-11A; US-EPA 1999).
**Participants**	Not reported
**Interventions/Exposure**	Air samples were collected at the sitting room of a flat owned by habitual smokers (volume: 48 m^3. 1.5 air changes/h), furnished with typical home appliances. During the experiments, the room was normally occupied by two to three people and equipped with real time analysers (placed 2 meters away from the smokers), samplers and three fans were always in operation during the smoking sessions and blowing in three different directions, two horizontally and one vertically, to assure homogeneity in the sampling environment and maximal mixing.<br>For IQOS, a total of 10 menthol and 14 regular tobacco sticks were tested; each session lasted for about 3 hours, during which tobacco sticks were consumed in cycles of 7 minutes, followed by 3 minutes pauses.<br>For e-cigarettes, 13 vaping sessions were performed and results reported as the average; e-cigarette vaping session lasted for 2–3 hours, with one puff every minute for 7 minutes, followed by 3 minutes pauses.<br>For cigarettes, nine were smoked in sequence, each for about 7 minutes with 3 minute pauses in between.
**Outcome/Key findings**	**Air pollution after the use of e-cigarette and HnB compared to cigarette secondhand tobacco smoke**

Pollutant	E-cigarette pollution as % of cigarette pollution (min%–max%)	HnB pollution as % of cigarette pollution (min%–max%)
370 nm UV BC (µg/m^3)	non-detectable levels	0.73–0.79
880 nm Standard BC (µg/m^3)	non-detectable levels	non-detectable levels
PM > 0.3 (particles/cm^3)	non-detectable levels	2.8–7.3
PM > 1.0 (particles/cm^3)	non-detectable levels	non-detectable levels
PM_{nm} (particles/cm^3)	5.7–7.0	22–24
PM 1 (µg/m^3)	non-detectable levels	0.92–1.0
PM 2.5 (µg/m^3)	non-detectable levels	1.3–1.5

	PM 10 (µg/m^3)	non-detectable levels	1.5–1.7
	Acrolein (µg/m^3)	non-detectable levels	1.8–2.3
	Acetaldehyde (µg/m^3)	0.23–0.29	5.0–5.9
	Formaldehyde (µg/m^3)	3.1–3.7	6.9–7.1
**Conclusions**	• Particulate matter emissions from IQOS were substantially higher compared to e-cigarette emissions but well below emissions associated with cigarette smoke<br>• Compared to cigarette smoke, IQOS and e-cigarettes emitted much lower levels of widely recognised carcinogens aldehydes, but these still pose risk for other people around.		
**Authors, study year**	**Mitova et al., 2016 [21]**		
**Funder/Affiliations**	**Affiliations:** Philip Morris International R&D, Philip Morris Products S.A., Quai Jeanrenaud 5, 2000 Neuchatel, Switzerland		
**Primary aim**	To compare levels of secondhand emissions by IQOS and cigarette in an indoor environment		
**Products used**	• IQOS: under Health Canada Intense (HCI) conditions yields 1.32 mg of nicotine and 0.53 mg of CO.<br>• Cigarette (Marlboro Gold from Swiss market): under HCI yields 1.7 mg nicotine and 22.9 mg CO.		
**Methods**	**Design:** Laboratory comparison study using smoking volunteers.<br>**Study time and setting:** Switzerland, time not reported<br>**Measures:** Particulate phase markers for environmental tobacco smoker (ETS) were determined according to ISO methods: ISO 15593 for gravimetric respirable suspended particulate matter (RSP), ultraviolet particulate matter (UVPM) and fluorescent particulate matter (FPM), and ISO 18144 for solanesol. Briefly, RSP was determined by weighing a polytetrafluoroethylene filter (37 mm diameter, 1 µm pore size) in triplicate on a microbalance (XP2U, Mettler Toledo, Greifensee, Switzerland) after overnight conditioning at 50 ±5% humidity. The average of the triplicate determinations was taken as the filter weight. After air sampling, the procedure was repeated, and the mass increase was reported as RSP. UVPM, FPM and solanesol were determined after extraction of the filter with 3mL methanol for background and IQOS assessments and 6 mL for assessments using Marlboro Gold. UVPM and FPM were determined simultaneously using ultra performance liquid chromatography (UPLC) with ultraviolet (UV) and fluorescence detection (Acquity, Waters Corporation, Milford, Massachusetts, USA). UVPM was determined at a wave length of 325 nm, and FPM at 300 nm excitation and 420 nm emission wavelengths. 2,2′,4,4′-Tetrahydroxybenzophenone and scopoletin were used as surrogate standards for UVPM and FPM, respectively. The determination of solanesol was performed using UPLC with UV detection at a wavelength of 205 nm (Acquity, Waters Corporation, Milford, Massachusetts, USA). 3-Ethenylpyridine and nicotine were determined using an adaption of the standard method ISO 18145 for use with gas chromatography-mass spectrometry (GC-MS; QP 2010 Ultra, Shimadzu Corporation, Kyoto, Japan). The analysis of VOCs (1,3-butadiene, acrylonitrile, benzene, isoprene, toluene) was performed using a method based on the National Institute for Occupational Safety and Health (NIOSH) standards 1024 and 1501 adapted for the inclusion of acrylonitrile and isoprene (which were not previously determined in the standard methods) in a single method. The air was sampled through a charcoal sorbent tube (Anasorb CSC, SKC, Blandford, UK), which was extracted with dichloromethane (1.5 mL) containing stable isotope-labelled internal standards (acrylonitrile-d3, benzene-d6, 1,3- butadiene-d6, toluene-d8), prior to analysis by GC-MS (QP-2010 Ultra; Shimadzu Corporation, Kyoto, Japan) operated in electron impact ionization (EI) mode. Low molecular weight carbonyl compounds (acetaldehyde, acrolein,		

	crotonaldehyde, formaldehyde) were trapped on a 2,4-dinitrophenylhydrazine (DNPH)-coated silica cartridge (Waters Corporation, Nilford, MA, USA) using a method based on ISO standard 16000-3 (International Organization for Standardization, 2011). The cartridge was eluted with acetonitrile (2 mL) and the DNPH-derivatives analysed by liquid chromatography-tandem mass spectrometry using atmospheric pressure chemical ionization (Triple Quad 5500; ABSciex, Framingham, MA, USA). CO was measured continuously using a nondispersive infrared detector (X-Stream™ Process Gas Analyzer, Emerson, Baar, Switzerland) calibrated using a certified gas standard (Carbagas AG, Guemlingen, Switzerland). NO and NOx were measured continuously using a chemiluminescence detector (APNA 370 Ambient NOx Monitor; Horiba, Baden, Switzerland) calibrated with certified gas standards (Messer Schweitz AG, Lenzburg, Switzerland).
**Participants**	Adult cigarette smokers (age: 21–60 years) with a regular daily cigarette consumption of at least 10 cigarettes with a 6mg ISO tar yield were recruited for participation in the study by a consumer panel recruiting agency.<br>A PMI representative was present during all assessments to ensure the panelists use the test products according to the established schedule. The PMI representatives for the background and IQOS assessments were non-smokers, while those for the Marlboro Gold assessments were adult smokers of cigarettes. The PMI representatives did not smoke or use any test products during the assessments.
**Interventions/Exposure**	All assessments (per simulation) lasted for 5 h during which time the smoking panelists used the test products according to a pre-defined time schedule. For instance, for the "Office" simulation, panelist 1 started to use the test product immediately at the beginning of the assessment period (t=0 min) and used a new test product at intervals of 30 min; smoking panelist 2 started to use the test product at t=15 min and used a new test product at intervals of 30 min (total of 4 test products per hour).<br>"Background" measurements of indoor air quality (IAQ) were performed for 4 h using the same ventilation conditions, but no test products were used. After each "background" session, a tracer gas method was used according to the International Organization for Standardization standard method ISO 16000-8 to confirm the ventilation rate in the environmentally controlled room. The room was flooded with carbon dioxide (CO_2) up to a concentration of 1% and the decay rate of CO_2 was measured over 8 h using a non-dispersive infrared instrument (X-Stream™ Process Gas Analyzer, Emerson Electric Co., St. Louis, MO, USA).
**Outcome/Key findings**	**Air constituents after using IQOS in 'Residential' (1.5 air changes per hour)* condition**

Constituent in the air	Median	% compared to cigarette smoke
**Secondhand tobacco smoke markers**		
Respirable suspended particles	< 14.7 μg/m^3	<6%
Nicotine	2.66 μg/m^3	9%
Solanesol	< 0.466 μg/m^3	<5%
3-Ethenylpyridine	< 0.243 μg/m^3	<3%
Ultra-violet particulate matter	< 0.789 μg/m^3	<2%
Fluorescent particulate matter	< 0.064 μg/m^3	<1%
**Carbonyls**		
Formaldehyde	22.4 μg/m^3	41%

	Acetaldehyde	12.5 μg/m^3	14%
	Crotonaldehyde	<0.182 μg/m^3	<9%
	Acrolein	< 0.146 μg/m^3	<3%
	**Volatile organic compounds**		
	Toluene	2.61 μg/m^3	9%
	Acrylonitrile	< 0.27 μg/m^3	<7%
	1,3-Butadiene	< 1.14 μg/m^3	<7%
	Isoprene	6.7 μg/m^3	6%
	Benzene	0.567 μg/m^3	6%
	**Gases**		
	Carbon monoxide	0.454 ppm	21%
	Nitrogen oxides	5.21 ppb	11%
	Nitrogen oxide	2.58 ppb	7%
	* Results for simulated 'hospitality' and 'office' conditions were similar		
**Findings overview**	The concentrations of all measured indoor air constituents were higher in the cigarette, compared to the background and IQOS sessions.		
**Authors, study year**	**O'Connell et al., 2016 [22]**		
**Funder/Affiliations**	**Affiliations:**<br>• Imperial Tobacco Ltd, 121 Winterstoke Road, Bristol, BS3 2LL, UK<br>• Reemtsma Cigarettenfabriken GmbH - Imperial Tobacco Group, Albert-Einstein-Ring 7, D-22761, Hamburg, Germany		
**Primary aim**	To compare levels of sidestream emissions by IQOS, e-cigarette, and nicotine inhalator		
**Products used**	• IQOS with regular tobacco sticks<br>• Nicorette® inhalator (15 mg nicotine replacement aid; McNeil Consumer Healthcare Ltd)<br>• Blu™ closed system e-cigarette (18 mg nicotine; Fontem Ventures B.V.)		
**Methods**	**Design:** Laboratory comparison study using smoking volunteers<br>**Study time and setting:** Switzerland, time not reported<br>**Method description:** Proton Transfer Reaction-Mass Spectrometry (PTR-MS) instrument ionizes volatile organic compounds (VOC) in the gas phase through their reaction with H_3O^+ to form protonated VOCs (VOCH+) which can then be detected by a mass spectrometer. Airspace analysis was conducted by connecting the PTR-MS inlet to the test chamber and sampling directly. PTR-MS operating conditions were as follows: drift tube voltage, 500 V; drift tube pressure, 2.3 mbar; drift tube temperature, 120°C; drift tube length, 9.3 cm; E/N ratio, 130 Td (Townsends; where E is electric field and N is the number density of the gas in the drift tube; 1 Td=10^{-17} cm^2 V $molecule^{-1}$); inlet temperature, 120°C.		
**Participants**	Not reported		

**Interventions/Exposure**	All products were used in accordance with manufacturer's instructions and consumed ad libitum i.e., there was no pre-defined consumption requirement. For each of the different products, a number of replicate puffs were made and representative data from a single puff were shown.
**Outcome/Key findings**	**Authors' described results**<br>• When IQOS was activated but not puffed, a large number of different volatile organic compounds species across a range of masses were released into the airspace<br>• Volatile organic compounds in the airspace around the nicotine inhalator and the e-cigarette during product use were virtually indistinguishable
**Authors' conclusions**	• IQOS produce sidestream emissions both while activated and used by a user, which raises a concern of second-hand exposure
**Authors, study year**	**Bekki et al., 2017** [16]
**Funder/Affiliations**	**Funded by:**<br>• The Health and Labour Science Research Grants from Ministry of Health<br>• Labour and Welfare of the Japanese Government<br>• The practical research project for life-style related diseases including cardiovascular diseases and diabetes mellitus from Japan Agency for Medical Research and Development, AMED.<br><br>**Affiliations:** Department of Environmental Health, National Institute of Public Health. Minami, Wako-shi, Saitama 351-0197, Japan
**Primary aim**	To compare levels of nicotine and HPHC in mainstream IQOS emissions from regular and menthol tobacco sticks with those in mainstream cigarette smoke
**Products used**	• Reference cigarettes 3R4F (high yield) and 1R5F (low yield)<br>• Regular and menthol IQOS
**Methods**	**Design:** Laboratory comparison study using smoking machines<br>**Study time and setting:** Japan, time not reported<br>**Measures:** yields of HPHC in mainstream aerosol from the regular and menthol IQOS tobacco sticks are compared to HPHC in mainstream smoke from the reference cigarettes
**Participants**	Not reported
**Interventions/Exposure**	Both IQOS and the reference cigarettes were smoked under HCI conditions. Each sampling was performed by 3 cigarettes and tobacco sticks, one cigarette was puffed 9 times, and one tobacco stick was puffed 11 times
**Outcome/Key findings**	• The average concentration of nicotine in IQOS regular tobacco sticks was 15.7 mg/g and in menthol tobacco sticks 17.1 mg/g.

	These estimates were similar to nicotine in smoke from reference cigarettes: 19.7 mg/g in 3R4F and 15.9 mg/g in 1R5F.<br>• IQOS showed higher nicotine transfer rate from tobacco sticks to aerosol than reference cigarettes: 23.4% (regular) and 23.5% (menthol) compared with 11.3% (3R4F) and 11.5% (1R5F).<br>• The concentration of tobacco specific nitrosamines were almost at the same ratio in IQOS tobacco sticks and in reference cigarettes.

Yields of HPHC in the mainstream aerosol of regular and menthol IQOS tobacco sticks and in mainstream smoke of reference cigarettes

	IQOS regular	IQOS menthol	3R4F cigarette	1R5F cigarette
HPHC	Mean ± SD	Mean ± SD	Mean ± SD	Mean ± SD
Total particulate matter (mg/stick)	44.0 ± 11.4	49.9 ± 8.6	36.9 ± 1.9	28.9 ± 2.3
Water (mg/stick)	33.1 ±10.2	35.3 ± 8.3	10.1 ± 0.9	8.8 ± 1.1
Tar (mg/stick)	9.8 ± 3.0	13.4 ± 2.2	25.2 ± 1.5	19.2 ± 1.3
Nicotine (mg/stick)	1.1 ± 0.1	1.2 ± 0.1	1.7 ± 0.1	1.0 ± 0.1
Carbon monoxide (mg/stick)	0.44 ± 0.04	0.43 ± 0.04	33.0 ± 1.8	29.7 ± 1.7
**Tobacco-specific nitrosamines**				
NAB (ng/stick)	4.5 ± 0.5	5.5 ± 0.6	30.4 ± 2.0	26.2 ± 0.5
NAT (ng/stick)	34.0 ± 3.1	37.2 ± 3.9	246.4 ± 16.9	183.1 ± 6.0
NNN (ng/stick)	19.2 ± 2.1	24.9 ± 3.5	311.1 ± 24.3	240.7 ± 6.6
NNK (ng/stick)	12.3 ± 1.5	13.8 ± 2.6	250.4 ± 13.7	107.0 ± 5.0
Total (ng/stick)	70.0 ± 7.2	81.4 ± 10.4	838.2 ± 53.7	557.1 ± 15.7

**Authors' conclusions**	• Although at lower concentrations than in the mainstream smoke of reference cigarettes, IQOS mainstream emissions definitely include toxic compounds which may have adverse effects on a user and bystanders.
**Authors, study year**	**Eaton et al., 2017** [23]
**Funder/Affiliations**	**Funded by:** British American Tobacco Investments Ltd<br>**Affiliations:** Research and Development, British American Tobacco Investments Ltd, Regents Park Road, Southampton, Hampshire SO15 8TL, UK
**Primary aim**	To compare levels of HPHC in mainstream glo emissions with those in mainstream cigarette smoke
**Products used**	• Reference cigarette 3R4F<br>• HnB device THP 1.0/glo with tobacco plug (blended Virginia tobacco processed by a paper-style reconstitution process)

**Methods**	**Design:** Laboratory comparison study using smoking machines<br>Study time and setting: UK, time not reported<br>**Measures:** yields of HPHC in mainstream aerosol from the glo tobacco sticks are compared to yields of HPHC in mainstream smoke from the reference cigarettes
**Participants**	Not reported
**Interventions/Exposure**	Both glo and the reference cigarette were smoked under HCI regimen. Each sampling was performed by 5 cigarettes and tobacco sticks, one cigarette was puffed until the butt mark was reached (10.3 puffs per cigarette), and a tobacco stick was puffed 8 times
**Outcome/Key findings**	• The maximum proximal heater temperature of glo was 240 ± 3°C. (see table below)

Outcome/Key findings

- The maximum proximal heater temperature of glo was 240 ± 3°C.

Yields of HPHC in the mainstream aerosol of glo tobacco sticks and in mainstream smoke of 3R4F reference cigarette

	THP 1.0/glo	3R4F cigarette
**HPHC**	**Mean ± SD**	**Mean ± SD**
Carbon monoxide (CO, mg/stick)	Not quantifiable (<0.233)	32 ± 0.9
Carbon dioxide (CO_2mg/stick)	2.35 ± 0.14	85.1 ± 4.0
Nitrogen oxide (NO, µg/stick)	10.1 ± 0.4	496 ± 16
Oxides of nitrogen (NO_X, µg/stick)	12.0 ± 0.4	553 ± 16
Acetaldehyde (µg/stick)	111 ± 8	2200 ± 103
Acrolein (µg/stick)	2.22 ± 0.52	157 ± 9
Benzo(a)pyrene (ng/stick)	Not quantifiable (<0.354)	12.9 ± 1.3
Benzene (µg/stick)	Not quantifiable (<0.056)	78.6 ± 4.6
1.3-Butadiene (µg/stick)	Below detection limit (<0.029)	108 ± 4
Formaldehyde (µg/stick)	3.29 ± 0.3	54.1 ± 6.0
NNN (ng/stick)	24.7 ± 2.5	263 ± 12
NNK (ng/stick)	6.6 ± 0.86	281 ± 16

**Authors' conclusions**	• The temperature of tobacco in the proximal and distal zones of glo/THP1.0 did not exceed 250°C<br>• Levels of HPHC in aerosol from glo/THP1.0 indicated very low thermal decomposition of the tobacco
**Authors, study year**	**Forster et al., 2017** [24]
**Funder/Affiliations**	**Funded by:** British American Tobacco Investments Ltd<br>**Affiliations:** Research and Development, British American Tobacco Investments Ltd, Regents Park Road, Southampton, Hampshire

	SO15 8TL, UK
**Primary aim**	To compare levels of HPHC in mainstream glo emissions with those in mainstream IQOS emissions and cigarette smoke
**Products used**	• Reference cigarette 3R4F (ISO tar yield 9.4 mg/cigarette in 9 puffs)<br>• Reference cigarette 1R6F (ISO tar yield 8.6 mg/cigarette in 7.5 puffs)<br>• Electronic heating device THP 1.0 (glo) with regular and menthol tobacco plugs (blended Virginia tobacco, Bright Tobacco Kent Neostiks and Intensely Fresh Kent Neostiks). Tobacco sticks were sourced from Japan<br>• IQOS with Essence tobacco stick sourced from Japan
**Methods**	**Design:** Laboratory comparison study using smoking machines<br>Study time and setting: UK, time not reported<br>**Measures:** yields of HPHC in the mainstream aerosol from the glo tobacco sticks compared to yields of HPHC in the mainstream emissions from the reference cigarettes and IQOS
**Participants**	Not reported
**Interventions/Exposure**	Glo/THP 1.0, the reference cigarettes and IQOS were smoked under HCI regimen. Five replicates were performed for HnB products with each sampling consisting of 5 tobacco sticks. For the reference cigarettes, five replicates were conducted for each with usually three cigarettes per replicate. One cigarette was puffed until the butt mark was reached (10.9 puffs per cigarette on average), and a tobacco stick was puffed 8 times
**Outcome/Key findings**	**Yields of HPHC in the mainstream aerosol of THP1.0 (glo) regular and menthol tobacco sticks, in mainstream smoke of 3R4F reference cigarette and in IQOS aerosol from regular tobacco stick**

	glo regular	glo menthol	3R4F cigarette	IQOS regular
**HPHC**	**Mean ± SD**	**Mean ± SD**	**Mean ± SD**	**Mean ± SD**
1,3-Butadiene (µg/stick)	BDL (0.029)	BDL (0.029)	108 ± 4	0.224 ± 0.016
1-Aminonaphthalene (ng/stick)	NQ (0.027)	NQ (0.027)	17.6 ± 0.6	0.030 ± 0.013
2-Aminonaphthalene (ng/stick)	NQ (0.012)	BDL (0.004)	13.2 ± 0.8	0.016 ± 0.008
4-Aminobiphenyl (ng/stick)	NQ (0.005)	NQ (0.005)	2.29 ± 0.12	NQ (0.005)
Acetaldehyde (µg/stick)	111 ± 8	115 ± 11	2200 ± 103	327 ± 20
Acrolein (µg/stick)	2.22 ± 0.52	2.50 ± 0.11	157 ± 9	9.98 ± 1.13
Acrylonitrile (µg/stick)	BDL (0.032)	BDL (0.032)	19.5 ± 1.6	NQ (0.107)
Ammonia (µg/stick)	4.01 ± 0.99	5.02 ± 0.49	32.5 ± 3.5	10.6 ± 0.7
Benzene (µg/stick)	NQ (0.056)	NQ (0.056)	78.6 ± 4.6	0.457 ± 0.029
Benzo[a]pyrene (ng/stick)	NQ (0.354)	0.356 ± 0.079	12.9 ± 1.3	0.582 ± 0.024

	Carbon monoxide (mg/stick)	NQ (0.223)	NQ (0.223)	32.0 ± 1.0	0.305 ± 0.017
	Crotonaldehyde (µg/stick)	0.567 ± 0.232	0.768 ± 0.321	42.0 ± 6.2	2.00 ± 0.40
	Formaldehyde (µg/stick)	3.29 ± 0.30	3.51 ± 0.54	54.1 ± 6.0	5.93 ± 0.87
	Isoprene (µg/stick)	NQ (0.135)	NQ (0.135)	887 ± 49	1.55 ± 0.20
	NNN (ng/stick)	24.7 ± 2.5	19.1 ± 2.2	263 ± 12	11.5 ± 0.8
	NNK (ng/stick)	6.61 ± 0.86	5.32 ± 0.89	281 ± 16	10.6 ± 0.2
	Toluene(µg/stick)	NQ (0.204)	NQ (0.204)	131 ± 5	1.33 ± 0.11
	Nicotine (mg/stick)	0.462 ± 0.037	0.365 ± 0.021	2.02 ± 0.08	1.16 ± 0.03
	Water (mg/stick)	12.1 ± 1.1	10.7 ± 0.9	15.1 ± 1.4	25.4 ± 2.0
	Glycerol (mg/stick)	3.02 ± 0.26	2.38 ± 0.21	2.35 ± 0.05	4.28 ± 0.08
	Total particulate matter (mg/stick)	26.1 ± 1.1	25.3 ± 1.4	46.9 ± 2.8	48.9 ± 0.7
	Nicotine-free dry particulate matter (mg/stick)	13.6 ± 1.2	14.2 ± 1.3	29.8 ± 1.4	22.3 ± 2.2
**Authors' conclusions**	• The levels of HPHC in the mainstream aerosol from glo were significantly reduced in comparison to the HPHC levels in smoke of a reference cigarette<br>• The HPHC levels of the glo aerosol were similar to IQOS aerosol composition				
**Authors, study year**	**Forster et al., 2017** [25]				
**Funder/Affiliations**	**Affiliations:** Research and Development, British American Tobacco Investments Ltd, Regents Park Road, Southampton, Hampshire SO15 8TL, UK				
**Primary aim**	To compare levels of secondhand smoke/emissions				
**Products used**	• Glo/THP 1.0 with regular tobacco sticks (Bright tobacco Kent Neostick)<br>• Cigarette: Lucky Strike regular cigarette (ISO tar yield 7 mg) and Du Maurier Silver cigarette (ISO tar yield 9 mg)				
**Methods**	**Design:** Laboratory comparison study using smoking machines<br>**Study time and setting:** United Kingdom, time not reported<br>**Measures:** During the tests, the parameters of CO_2,CO, NO_x, ozone (O_3) and particulate matter by size (diameter PM1 = ≤ 1 µm, PM2.5 = 2.5 µm, and PM10 = 10 µm) in the test room were measured continuously every 60 seconds. In addition, PM1, PM2.5, PM10, NOx and O_3 were monitored outside the test room building. The following air constituents were sampled continuously over the total 4 hours of the test inside the test room: individual and total volatile organic compounds; low-molecular-weight carbonyl compounds (formaldehyde, acetaldehyde, acrolein and crotonaldehyde); polycyclic aromatic hydrocarbons (PAHs); nicotine; glycerol; 3-ethenyl pyridine (3-EP); and tobacco specific nitrosamines (TSNAs). Particle size, mass and number concentration were also measured continuously every 10 seconds with an electrical-mobility spectrometer.				
**Participants**	Adult cigarette smokers (minimum age 22 years; minimum daily cigarette consumption six cigarettes) who had smoked for at least				

	18 months were recruited by a specialist agency. Four participants were present in the test room at any time, along with an independent non-smoking moderator.
**Interventions/Exposure**	Prior to the tests, no smoking or vaping had previously taken place in the test room, which had been maintained under natural ventilation conditions (i.e., no air conditioning or openable windows).<br>Glo and the Lucky Strike Regular reference cigarette were tested in duplicate at all three ventilation conditions; the Du Maurier Silver cigarette was tested only at the lowest 1.2 air changes per hour ventilation (i.e., the highest-concentration condition). Five test situations, each with a 4 hours sampling period, were conducted per week in three stages, corresponding to the three ventilation conditions. Cigarettes were always smoked last in the week to minimise carryover contamination, and the room ventilation continued to operate overnight and at weekends to flush out potential residual contaminants. Smokers were asked to take a puff once every 30 s for 8 puffs. All product use was completed while volunteers sat in the chairs provided. At other times, the volunteers were free to leave their chairs, but they were asked to not stand in the direct vicinity of the monitoring equipment in order to keep environmental interference to a minimum.
**Outcome/Key findings**	**HPHC levels in secondhand emissions of glo, Lucky Strike Regular & Du Maurier Silver cigarettes in 'Home'* condition (1.2 air changes per hour)** (table below)

Constituent in the air	glo	Lucky Strike Regular	Du Maurier Silver
1,3-Butadiene (µg•m^{-3})	Below detection limit	Below detection limit	Below detection limit
Isoprene (µg•m^{-3})	16	191	255
Acrylonitrile (µg•m^{-3})	Below detection limit	Below detection limit	Below detection limit
Benzene (µg•m^{-3})	1	16	21
Toluene (µg•m^{-3})	3	29	32
Propylene glycol (µg•m^{-3})	Below detection limit	Below detection limit	Below detection limit
Acrylamide (µg•m^{-3})	Below detection limit	Below detection limit	Below detection limit
Total volatile organic compounds (µg•m^{-3})	49	373	362
Formaldehyde (µg•m^{-3})	17.5	33.3	43.0
Acetaldehyde (µg•m^{-3})	10	100	118
Acrolein (µg•m^{-3})	Below detection limit	Below detection limit	Below detection limit
Crotonaldehyde (µg•m^{-3})	Below detection limit	Below detection limit	Below detection limit
Nicotine (µg•m^{-3})	0.3	47.0	33.0
3-ethenyl pyridine (µg•m^{-3})	Below detection limit	9.1	7.8
Glycerol (µg•m^{-3})	Below detection limit	Below detection limit	Below detection limit

Carbon monoxide (ppm)	Below detection limit	Below detection limit	1.3
NO (ppb)	4	30	22
NO_2 (ppb)	8	12	11
NO_x (ppb)	12	42	33
PM_1	7.1	1392	1529
$PM_{2.5}$	7.4	1392	1536
PM_{10}	12.8	1398	1541

* Results for simulated ‘hospitality’ and ‘office’ conditions were similar

**Findings overview**	• Glo emissions have a significantly lower impact on indoor air quality than conventional combustible cigarettes, driven by a significantly lower emissions profile of both aerosol particles and chemical emissions in respect of the HPHC measured
**Authors, study year**	**Protano et al., 2017** [26]
**Funder/Affiliations**	**Affiliations**:<br>• Department of Public Health and Infectious Diseases, Sapienza University of Rome, Italy<br>• Department of Technological Innovations, INAIL, Rome, Italy<br>• Department of Agricultural, Environmental and Food Sciences, University of Molise, Italy
**Primary aim**	To compare levels of secondhand smoke/emissions from real use of regular and hand-rolled cigarettes, pipes, cigars, e-cigarettes, and IQOS
**Products used**	• Cigarette: Pall Mall San Francisco (0.7 mg nicotine, 8 mg tar, and 9 mg carbon monoxide)<br>• Hand-rolled cigarette: Golden Virginia tobacco rolled with a Rizla Blue Regular rolling paper<br>• Cigar: Italian Toscanello<br>• Pipe: Amphora Original Blend tobacco<br>• IQOS: Marlboro Balance stick<br>• E-cigarette: pen-style Smooke E-Smart with Smooke Light e-liquid (9 mg ml of nicotine)
**Methods**	**Design:** Laboratory comparison study using smoking volunteers<br>**Study time and setting:** Italy, time not reported<br>**Measures:** Aerosol number-size distributions were measured by using a TSI Fast Mobility Particle Sizer (model 3091, FMPS, Shoreview, MN, USA). The instrument counts and classifies particles according to their electrical mobility in 32 size channels in the range of 5.6 to 560 nm with a temporal resolution of 1 s. FMPS operates at high flow rate (10 L min−1) to minimize diffusion losses and at ambient pressure to prevent the evaporation of volatile and semi-volatile particles.

**Participants**	Four volunteer smokers (three male, age range 37–60), employees of the Sapienza University Rome.
**Interventions/Exposure**	Six sets of experiments (one for each smoking device) were carried out in triplicate; each experiment was based on one or more smoking sessions, which were performed by volunteers who were currently smokers in a 52.7m^3 test room with a door and window that were both closed.<br>Three smoking sessions at 1-h time intervals for each smoking device (conventional cigarette, hand-rolled cigarette, e-cig and IQOS) were performed. During each session, a single cigarette or IQOS stick was smoked. For the e-cigarette, 12 puffs per session were taken. Since cigars and tobacco pipes are typically smoked differently than cigarettes, they were smoked in a single smoking session until the cigar or pipe tobacco was finished, which resulted in longer time intervals than for the other devices (approximately 30 and 45 min, respectively).<br>For each type of smoking device, aerosol measurement started 5 min before the first smoking session and lasted 200 min in order to follow the aerosol concentration decay. Before changing the smoking device, the door and window were opened to allow the atmosphere of the room to rebalance.
**Outcome/Key findings**	• A one hour period after each smoking session of conventional and hand-rolled cigarettes was not enough for the particle concentration to decrease to the background level<br>• Particle emissions from the e-cigarettes were lower than from IQOS but e-cigarettes produces higher peak values for particle emissions compared to IQOS
**Findings overview**	• The tested e-cigarette and IQOS devices emitted submicronic particles during their use, which supports the ban of 'electronic' nicotine delivery devices indoors
**Authors, study year**	**Jaccard et al., 2017** [27]
**Funder/Affiliations**	**Affiliations:** Philip Morris International R&D, Philip Morris Products SA, Rue des Usines 56, CH-2000 Neuchatel, Switzerland
**Primary aim**	To compare levels of HPHC in mainstream IQOS emissions with those in mainstream cigarette smoke
**Products used**	• Reference cigarette 3R4F (ISO tar yield 9.4 mg/cigarette in 9 puffs)<br>• IQOS/THS 2.2 with regular tobacco sticks<br>• Commercial cigarettes samples obtained from South Korea, Germany, Russia, Japan, Australia and EU countries
**Methods**	**Design:** Laboratory comparison study using smoking machines<br>**Study time and setting:** not reported

	**Measures:** yields of HPHC in the mainstream aerosol from the IQOS tobacco sticks are compared to yields of HPHC in the mainstream smoke from the standardised reference cigarette 3R4F and commercially available cigarettes
**Participants**	Not reported
**Interventions/Exposure**	For IQOS, reference cigarettes, and commercial cigarettes all analyses were performed according to the official Health Canada methods
**Outcome/Key findings**	**Yields of HPHC in the mainstream emissions of IQOS regular tobacco sticks and 3R4F reference cigarette**

	**IQOS**	**3R4F cigarette**
**HPHC**	**Mean ± SD**	**Mean ± SD**
1,3-Butadiene (µg/stick)	0.342 ± 0.0347	98.5 ± 9.8
1-Aminonaphthalene (ng/stick)	0.0407 ± 0.0103	21.6 ± 2.28
2-Aminonaphthalene (ng/stick)	0.0277 ± 0.00909	16.2 ± 2.54
4-Aminobiphenyl (ng/stick)	0.00958 ± 0.0014	2.83 ± 0.434
Acetaldehyde (µg/stick)	217 ± 7.85	1641 ± 258
Acrolein (µg/stick)	9.63 ± 0.703	156 ± 25.4
Acrylonitrile (µg/stick)	0.158 ± 0.0122	24.5 ± 3.52
Ammonia (µg/stick)	10.5 ± 1.63	29.3 ± 2.88
Benzene (µg/stick)	0.544 ± 0.0312	81.1 ± 8.78
Benzo[a]pyrene (ng/stick)	0.939 ± 0.0796	15 ± 1.3
Carbon monoxide (mg/stick)	0.436 ± 0.0811	30.2 ± 2.76
Crotonaldehyde (µg/stick)	<3.29	50.5 ± 9.42
Formaldehyde (µg/stick)	7.98 ± 0.504	85.2 ± 16.7
Isoprene (µg/stick)	2.15 ± 0.202	894 ± 76.7
NNN (ng/stick)	10.2 ± 0.486	283 ± 27.8
NNK (ng/stick)	6.75 ± 0.493	264 ± 26.4
Toluene(µg/stick)	1.82 ± 0.163	137 ± 16.9
Nicotine (mg/stick)	1.14 ± 0.0332	1.86 ± 0.175
Water (mg/stick)	Not measured	Not measured
Glycerol (mg/stick)	Not measured	Not measured
Total particulate matter (mg/stick)	Not measured	Not measured
Nicotine-free dry particulate matter (mg/stick)	Not measured	Not measured

**Authors' conclusions**	• In comparison with HPHC levels in the mainstream smoke of 3R4F cigarettes, the average reduction over all analysed HPHC in IQOS is found to be 92% on a per tobacco stick basis and 87% on a nicotine-adjusted basis.<br>• In comparison with the HPHC in the mainstream smoke of commercial cigarettes, the mean reduction observed for the IQOS

	aerosol HPHC is close to the reduction for 3R4F: 90–92% reduction for the per tobacco stick basis using HCI puffing regimen and 83–88% reduction for the nicotine-adjusted results.
**Authors, study year**	**Poynton et al., 2017** [28]
**Funder/Affiliations**	**Affiliations:** Research and Development, British American Tobacco Investments Ltd, Regents Park Road, Southampton, Hampshire SO15 8TL, UK
**Primary aim**	To compare levels of HPHC in mainstream iFuse emissions with those in mainstream pen-style e-cigarette emissions and cigarette smoke
**Products used**	• Reference cigarette 3R4F (ISO tar yield 9.4 mg/cigarette in 9 puffs)<br>• E-cigarette: pen-style Vype ePen I (Nicoventures trading Ltd, Blackburn, UK)<br>• Hybrid tobacco product (commercially available as iFuse in Romania): a button operated electronic vapour device consisting of USB-rechargeable battery and a closed-system, disposable neopod cartomizer The cartomizer comprises of an atomizer, a liquid tank (1.15 ml of non-flavoured liquid composed of propylene glycol, vegetable glycerine, water, and nicotine), and a chamber containing a 130 mg blended tobacco plug. When activated, the user's drawn warm aerosol goes up through the plug of tobacco and takes volatile tobacco flavour compounds giving sensory characteristics of the tobacco used. The battery life of the device allows at least 300 puffs from a single charge, which is sufficient for a single neopod.
**Methods**	**Design:** Laboratory comparison study using smoking machines<br>**Study time and setting:** not reported<br>**Measures:** yields of HPHC in the mainstream aerosol from the iFuse are compared to yields of HPHC in the mainstream smoke from the standardized reference cigarette 3R4F and the e-cigarette
**Participants**	Not reported
**Interventions/Exposure**	• The 3R4F reference cigarette was machine-smoked using the HCI regimen. Emissions data collected on a per-cigarette basis with puff number collected.<br>• Both iFuse and the e-cigarette were machine-smoked using the following puffing regime: 55 ml puff volume, 3 seconds puffing duration, 30 seconds inter-puff interval, with devices' voltage set at 3.6 V. The analyses for each two products were conducted in two blocks of 100 puffs, and the levels of emissions were averaged on a per-puff basis.
**Outcome/Key findings**	**Yields of HPHC in the mainstream aerosol of iFuse and pen-style e-cigarette, and in the mainstream smoke of 3R4F reference cigarette**

	iFuse	E-cigarette	3R4F cigarette
**HPHC**	**Mean ± SD[a] (per 100**	**Mean ± SD[a] (per 100**	**Mean ± SD**

	puffs)	puffs)	
1,3-Butadiene (µg/stick)	<0.29	<0.29	91.8 ± 5.6
1-Aminonaphthalene (ng/stick)	<0.27[b]	0.49 ± 0.32	19.3 ± 3.2
2-Aminonaphthalene (ng/stick)	0.40 ± 0.19	0.82 ± 0.38	12.5 ± 0.5
4-Aminobiphenyl (ng/stick)	0.06 ± 0.04	0.17 ± 0.10	2.14 ± 0.50
Acetaldehyde (µg/stick)	8.53 ± 1.67	10.7 ± 2.9	1732 ± 43
Acrolein (µg/stick)	8.38 ± 9.45	7.90 ± 5.56	172 ± 3
Acrylonitrile (µg/stick)	<0.32	<0.32	21.4 ± 1.8
Ammonia (µg/stick)	<14.63	<4.39	29.5 ± 2.0
Benzene (µg/stick)	<0.17	<0.17	72.9 ± 7.4
Benzo[a]pyrene (ng/stick)	<1.06	<1.06	14.3 ± 1.6
Carbon monoxide (mg/stick)	6.31 ± 0.71	6.75 ± 0.28	29.6 ± 1.5
Crotonaldehyde (µg/stick)	<1.98	<1.98	57.0 ± 1.7
Formaldehyde (µg/stick)	12.2 ± 5.2	12.3 ± 4.9	94.9 ± 6.2
Isoprene (µg/stick)	<0.41	<0.41	847 ± 59
NNN (ng/stick)	2.20 ± 0.59	<1.97	265 ± 22
NNK (ng/stick)	<3.01	<3.01	283 ± 24
Toluene(µg/stick)	2.53 ± 0.14	2.64 ± 0.22	116 ± 9
Nicotine (mg/stick)	2.56 ± 1.33	3.57 ± 1.10	1.84 ± 0.08

Note: [a] out of the two puffing blocks (1–100 and 101–200) the highest measured mean of toxicants was used

[b] If measured levels were below quantification or detection limits, the lowest value was then used

**Authors' conclusions**	• The temperature of the aerosol of iFuse had an average maximum of 35 °C before reaching the tobacco and decreased to an average maximum of 32 °C after the tobacco plug.<br>• The nicotine measured in the aerosol originated almost exclusively from the liquid rather than from the tobacco.<br>• The nicotine levels measured in the two puff blocks of the iFuse were slightly lower than those of the tested e-cigarette<br>• The emission levels from iFuse HnB product were comparable to those measured from the e-cigarette, and were 92–99% lower on a per-puff basis than those from the 3R4F cigarette
**Authors, study year**	**Pratte et al., 2017** [29]
**Funder/Affiliations**	**Affiliations:** Philip Morris International R&D, Philip Morris Products S.A., Neuchatel, Switzerland
**Primary aim**	To compare numbers of solid particles in mainstream IQOS emissions with those in mainstream cigarette smoke
**Products used**	• 3R4F reference cigarette<br>• IQOS with regular tobacco sticks

**Methods**	**Design:** Laboratory study of mainstream smoke and aerosol compositions from two different tobacco products<br>Study time and setting: not reported<br>**Measures:** the collection of solid particles from reference cigarette smoke and IQOS aerosol
**Participants**	Not reported
**Interventions/Exposure**	The test products were machine smoked using HCI puffing regimens
**Outcome/Key findings**	• In the mainstream smoke from a 3R4F cigarette approximately 10^{12} solid particles of the median diameter of approximately 75 nm (ultrafine particles) were identified<br>• No solid particles were accumulated from the mainstream aerosol of IQOS in comparison to the blank test
**Authors' conclusions**	• Heated tobacco products neither generate nor transfer solid particles in the mainstream aerosol when considering applied experimental conditions

Table A3 Findings of the studies on heat not burn use by human participants

**Authors, study year**	**Lopez et al., 2016** [7]
**Funder/Affiliations**	**Funded by:** National Institute on Drug Abuse of the National Institutes of Health under Award Number P50DA036105 and the Center for Tobacco Products of the U.S. Food and Drug Administration<br>**Affiliations:** Virginia Commonwealth University, Department of Psychology and Center for the Study of Tobacco Products
**Primary aim**	To compare nicotine delivery, expired air CO concentration and abstinence symptom suppression
**Products used**	• Pax loose-leaf tobacco vaporiser (LLTV): pre-filled with 1 g of Zig Zag brand loose-leaf tobacco (produced by National Tobacco Company, Louisville, Kentucky). Tobacco or menthol flavour was matched to the participants' preferred own brand cigarettes' flavour<br>• Own brand cigarettes<br>• E-cigarette: pen-style 'eGo', 3.3 V, 1000 mAh e-cigarette battery attached to a 1.5 ohm, dual coil, 510-style cartomizer (Smok-Tech; Shenzhen, China). The cartomizer was pre-loaded with approximately 1 ml of 18 mg/ml nicotine liquid (70% propylene glycol and 30% vegetable glycerine) (AVAIL Vapor, Richmond, Virginia). Tobacco or menthol liquid flavour was matched to the participants' preferred brand cigarettes' flavour
**Methods**	**Design:** Randomised crossover experimental trial<br>**Recruitment:** smokers recruited by advertisements and word of mouth<br>**Study date and setting:** time not reported; Virginia Commonwealth University's (VCU) Clinical Behavioral Pharmacology Laboratory, Virginia USA<br>**Protocol registered:** not registered<br>**Inclusion criteria:** healthy; 18–55 years old; smoked ≥10 cigarettes per day (CPD); had used an e-cigarette ≤ 20 times and a LLTV < 5 times in their lifetime<br>**Exclusion criteria:** history of chronic disease or psychiatric condition; regular prescription medication use (aside from birth control); marijuana use >10 days and alcohol use >25 days in the past 30 days; use of a vaporiser for marijuana >5 times in their lifetime; any illicit drug use (e.g. cocaine, opioids, benzodiazepines, and methamphetamine) in the past 30 days; tested positive for pregnancy
**Participants**	Forty provided informed consent, 16/40 (40%) did not meet the eligibility criteria; 9/24(38%) discontinued the study<br>N=15; 80% male; mean age 33.6; 47% white, 40% Black/African American; mean CPD=16.1; mean Fagerström Test for Nicotine Dependence (FTND)=5.1
**Interventions/Exposure**	Three 2.5-hour sessions where participants used different products. Sessions were separated by a minimum 48 hours with washout period (abstinence from nicotine/tobacco) of at least 12 hours. In each session participants completed two 10-puff product use bouts (30 s inter-puff intervals) separated by 60 minutes.
**Outcome/Key findings**	

	Pax	Cigarette	E-cigarette
**Plasma nicotine concentration,** ng/ml (SD), Cohen's d			
Bout 1	14.3 (8.1), d=1.2	24.4 (12.6), d=2.5	9.5 (8.5), d=2.0
Bout 2	16.4 (11.3), d=1.7	23.7 (14.5), d=2.1	9.5 (7.5), d=1.4
**Expired air CO concentration,**			

	ppm (SD), Cohen's d			
	Bout 1	ppm (SD) not provided, d=-0.2	12.1 (3.4), d=2.1	Not provided
	Bout 2	4.5 (2.1), d=-0.5	16.9 (5.8), d=2.5	4.5 (1.7), d=-0.7
	**Abstinence symptom suppression,** initial score (SD) – score after use (SD), Cohen's d			
	Bout 1	23.8 (8.7) – 16.1 (9.9), d=0.8	25.2 (6.4) – 10.8 (8.6), d=2.0	Non-significant difference
	Bout 2	Non-significant difference	Not reported – 7.7 (8.3), d=2.4	Non-significant difference

**Findings overview**	• Pax use significantly increased plasma nicotine concentration, did not increase expired air CO concentration, and significantly reduced abstinence symptom severity in smokers<br>• Pax and the e-cigarette use were significantly less satisfying than cigarettes
**Authors, study year**	**Brossard et al., 2017** [3]
**Funder/Affiliations**	**Affiliations:** Philip Morris International R&D, Philip Morris Products S.A., Neuchatel, Switzerland
**Primary aim**	To compare nicotine delivery and effects on urge to smoke
**Products used**	• IQOS with regular (under ISO: 4 mg tar, 0.5 mg nicotine, 1 mg CO per stick) and menthol (5 mg tar, 0.5 mg nicotine, 1 mg CO) tobacco sticks<br>• Regular or menthol cigarettes preferred by participants (nicotine ISO yields ≤1 mg)<br>• Non-menthol 2 mg Nicorette® chewing gum (1.06 mg nicotine per chewed gum)
**Methods**	**Design:** Randomised crossover experimental trial<br>**Recruitment:** via the database of the two participating clinics<br>**Study date and setting:** July–November 2013 at Koganeibashi Sakura Clinic, Tokyo, and August–November 2013 at Ageo Medical Clinic, Saitama, Japan<br>**Protocol registered:** 8 October, 2013 (NCT01959607 at clinicaltrials.gov) & 18 October, 2013 (NCT01967706 at clinicaltrials.gov)<br>**Inclusion criteria:** healthy; 23–65 years old; smoked ≥10 CPD (max yield of 1 mg nicotine/cig) for the last 4 weeks; had smoked for ≥3 years prior recruitment; not willing to quit smoking in the forthcoming 3 months; ready to accept interruptions of smoking for up to four consecutive days, and willing to use THS and nicotine gum instead of smoking, the body mass index range of 18.5–32 kg/m^2, urinary cotinine ≥ 200 ng/mL<br>**Exclusion criteria:** participants with clinically relevant medical conditions, with a history of alcohol and/or drug abuse, pregnant or breast feeding females.
**Participants**	110 participants were screened for regular tobacco sticks study & 147 for menthol tobacco sticks study (Tokyo and Saitama clinics, respectively); 45/110 (41%) & 74/147 (50%) did not meet the eligibility criteria, 3/65 (5%) & 11/73 (15%) were not randomised, 2/62 (3%) & 1/62 (2%) dropped out<br>N(regular)=60 & N(menthol)=61, 52.5% & 55.0% male, mean age 34 ± 9.18 & 32.6 ± 9.44, 56.7% & 59% smoked ≤20 CPD.
**Interventions/Exposure**	Regular and menthol groups were randomised to four sequences: IQOS → cigarette (n=22), cigarette → IQOS (n=22), IQOS→ gum (n=9), gum → IQOS (n=9)<br>• Sequence 1: 24 hour wash-out period, single use of IQOS, 24 hour wash-out period, single use of cigarette

<table>
<tr><td></td><td>

- Sequence 2: 24 hour wash-out period, single use of cigarette, 24 hour wash-out period, single use of IQOS
- Sequence 3: 24 hour wash-out period, single use of IQOS, 24 hour wash-out period, single use of gum
- Sequence 4: 24 hour wash-out period, single use of gum, 24 hour wash-out period, single use of IQOS

</td></tr>
<tr><td>Outcome/Key findings</td><td>

Nicotine concentration pharmacokinetics of tobacco sticks in comparison with cigarettes and nicotine gum

Pharmacokinetic parameter	Ratio IQOS : Cigarette*		Ratio IQOS: Gum	
	Regular	Menthol	Regular	Menthol
C_{max}	103.5% (84.9–126.1)	88.5% (68.6–114.0)	240.2% (130.6–441.9)	101.6% (62.2–166.0)
$t_{1/2}$	93.1% (84.6–102.4)	102.3% (85.3–122.7)	87.3% (65.6–116.3)	92.1% (73.6–115.2)
$AUC_{0\text{-last}}$	96.3% (85.1–109.1)	98.1% (80.6-119.5)	127.2% (77.3–209.2)	55.9% (38.4–81.4)
t_{max} (minutes)	6 min : 6 min	6 min: 6 min	6 min : 35.4 min	8 min : 45 min

* Regular tobacco sticks were compared with regular cigarettes and menthol tobacco sticks were compared with menthol cigarettes
Note: C_{max}: maximum nicotine concentration; $t_{1/2}$: terminal half-life; $AUC_{0\text{-last}}$: area under plasma concentration-time curve from start of product use extrapolated to the last measurable concentration; t_{max}: time to maximum plasma concentration

Nicotine concentration pharmacokinetics of regular and menthol tobacco sticks (geometric least squares means)

Pharmacokinetic parameter	Trial 1			Trial 2		
	Regular	Menthol	Regular : Menthol ratio	Regular	Menthol	Regular : Menthol ratio
C_{max} (ng/mL)	14.30	10.70	133.6%	11.53	7.64	150.9%
$t_{1/2}$ (h)	3.81	4.11	92.7%	4.16	3.20	130.0%
$AUC_{0\text{-last}}$ (ng*h/mL)	23.75	23.99	99.0%	18.92	15.61	121.2%
t_{max} (minutes)	Median=6	Median=6	no difference	Median=6	Median=8	-2 minutes

Note: C_{max}: maximum nicotine concentration; $t_{1/2}$: terminal half-life; $AUC_{0\text{-last}}$: area under plasma concentration-time curve from start of product use extrapolated to the last measurable concentration; t_{max}: time to maximum plasma concentration

Urge to smoke scores after use of IQOS and gum

- Maximum suppression after start of product use: IQOS=15–30 min, cigarette=15–30 min, gum=45–60 min
- Least square mean differences (95% CI) over all time points:
- IQOS (regular) - cigarette (regular) = 0.04 (-0.70–0.79)
- IQOS (menthol) - cigarette (menthol) = -0.28 (-0.79–0.22)
- IQOS (regular) - Gum = -0.20 (-0.87–0.48)
- IQOS (menthol) - Gum = -0.34 (-0.87–0.19)

</td></tr>
<tr><td>Findings overview</td><td>

- Use of regular and menthol IQOS provided similar peak and total exposure to nicotine concentrations when compared with smoking regular

</td></tr>
</table>

	and menthol cigarettes<br>• When compared with nicotine gum, regular IQOS provided twice as high peak nicotine concentration than gum, while menthol IQOS and gum peak nicotine concentrations were similar. Regular IQOS use provide slightly longer exposure to nicotine concentrations than chewing nicotine gum, while after using menthol IQOS total exposure to nicotine is almost twice as shorter than using nicotine gum.<br>• Time to maximum plasma nicotine concentration was comparable between IQOS and cigarettes (around 6 minutes) but six times longer for nicotine gum (35–45 minutes)<br>• Nicotine half-life was comparable between using both types of IQOS tobacco sticks, cigarettes and nicotine gum<br>• Gum, cigarettes and regular and menthol IQOS reduced urges to smoke similarly
**Authors, study year**	**Haziza et al., 2016** [8]
**Funder/Affiliations**	**Affiliations**: Philip Morris International R&D, Philip Morris Products S.A., Neuchatel, Switzerland
**Primary aim**	To compare exposure to HPHC during 5 days of use
**Products used**	• IQOS<br>• Cigarette: participants' preferred brand
**Methods**	**Design:** Randomised controlled trial<br>**Recruitment:** via the clinical site's database and through advertisements<br>**Study date and setting:** July 2013, Higashi Shinjuku Clinic, Tokyo, Japan<br>**Protocol registered:** 18 October, 2013 (NCT01970982 at clinicaltrials.gov)<br>**Inclusion criteria:** healthy; 23–65 years old; smoked ≥10 non-mentholated CPD (max yield of 1 mg nicotine/cig) for the last 4 weeks; had smoked for ≥3 years prior recruitment; not willing to quit smoking in the forthcoming 3 months; ready to accept a 5-day smoking interruption<br>**Exclusion criteria:** participants with clinically relevant medical conditions, those who required medical interventions (start of treatment, surgery, or hospitalization), a history of alcohol and/or drug abuse, used nicotine containing products other than cigarettes, pregnant or breast feeding, and females not using effective contraception
**Participants**	267 screened; 101/267 (38%) did not meet eligibility criteria; 6/166 (4%) dropped out before randomisation<br>N=160; 50% male; mean age 37.1; mean FTND=4.4<br>Dropped out: 2/40 in abstinence group
**Interventions/Exposure**	Randomised 2:1:1 to IQOS (n=80), cigarette (n=40), and abstinence (n=40) conditions for 5 days in confinement.<br>• IQOS group participants were asked to *ad libitum* use exclusively IQOS tobacco product<br>• Cigarette group participants were asked to *ad libitum* use exclusively their own brand of cigarettes<br>• Abstinence group participants were asked to completely abstain from smoking for five days. The use of nicotine replacement therapy was not allowed
**Outcome/Key findings**	**Levels of biomarkers of exposure to HPHC at day 5 in smoking, abstinence and IQOS groups**

**Parent harmful and potentially harmful compound**	**Geometric mean (95% CI) of exposure levels to HPHC biomarkers**		
	**Smoking group**	**Abstinence group**	**IQOS**
Carbon monoxide (%)	5.14 (4.66; 5.66)	2.37 (2.28; 2.47)	2.39 (2.32; 2.46)

Acrolein (ng/mg creat)	599.67 (511.70; 702.76)	199.04 (173.02; 228.97)	311.08 (279.59; 346.12)
1,3-butadiene (pg/mg creat)	450.19 (300.07; 675.42)	92.18 (80.18; 105.98)	107.39 (97.24; 118.60)
Benzene (pg/mg creat)	850.02 (620.40; 1164.63)	126.34 (105.51; 151.28)	143.77 (126.08; 163.93)
Nicotine-derived nitrosamine ketone (NNK) (pg/mg creat)	76.55 (59.76; 98.04)	28.63 (21.02; 39.00)	37.77 (31.43; 45.38)
Pyrene (pg/mg creat)	149.62 (132.68; 168.72)	62.99 (53.07; 74.75)	73.02 (65.19; 81.79)
N-nitrosonornicotine (NNN) (pg/mg creat)	4.64 (3.51; 6.12)	0.18 (0.15; 0.22)	1.31 (1.06; 1.61)
4-Aminobiphenyl (pg/mg creat)	8.57 (7.11; 10.34)	1.49 (1.29; 1.72)	1.53 (1.37; 1.70)
1-aminonaphthalene (pg/mg creat)	57.08 (48.55; 67.11)	2.45 (2.12; 2.82)	2.47 (2.23; 2.72)
2-aminonaphthalene (pg/mg creat)	13.38 (10.93; 16.37)	2.27 (1.96; 2.63)	2.33 (2.10; 2.59)
o-toluidine (pg/mg creat)	98.18 (82.69; 116.57)	48.91 (40.56; 58.97)	50.4 (44.64; 56.91)
Acrylonitrile (ng/mg creat)	54.19 (43.47; 67.55)	9.04 (7.05; 11.60)	10.61 (9.17; 12.29)
Ethylene oxide (pg/mg creat)	2099.41(1614.33;2730.24)	806.29 (666.35; 975.61)	997.76 (866.57; 1148.82)
Crotonaldehyde (ng/mg creat)	157.83 (128.07; 194.51)	47.84 (40.62; 56.34)	59.51 (53.40; 66.30)
Benzo(a)pyrene (fg/mg creat)	96.42 (80.55; 115.41)	24.47 (20.70; 28.91)	29.52 (26.01; 33.50)
Nicotine equivalents (mg/g creat)	5.52 (4.58; 6.66)	0.15 (0.12; 0.19)	5.44 (4.61; 6.41)
Nicotine (ng/ml)	21.34 (18.56; 24.55)	0.10 (0.09;0.11)	19.13 (15.60;23.46)
Cotinine (ng/ml)	164.30 (130.93; 206.17)	2.96 (1.96; 4.46)	161.00(131.19; 197.57)

Note: creat: creatinine

Daily use of tobacco sticks and cigarettes

Time	Mean (SD) IQOS tobacco sticks	Mean (SD) cigarettes	% IQOS:Cigarettes
Day 1	8.3 (3.0)	10.6 (3.1)	78.3%
Day 5	9.9 (3.9)	12.5 (3.5)	79.2%

Human puffing topography:

- At day 1, IQOS group compared with cigarette group:
  - Average puff volume 25% lower
  - Total puff volume 18% lower
  - Number of puffs 11% higher
  - Puff frequency 18% higher
  - Puff duration 11% longer
- At day 4: IQOS group compared with cigarette group:
  - Number of puffs 19% higher

	○ Puff frequency 27% higher<br>○ Puff duration 23% longer<br><br>**Differences in modified cigarette evaluation subscales' scores (IQOS - cigarette):**<br>• Smoking satisfaction: **-0.69 (-1.04, -0.34)**<br>• Aversion: 0.01 (-0.19, 0.21)<br>• Craving reduction: -0.17 (-0.59, 0.25)<br>• Enjoyment of respiratory tract sensation: -0.34 (-0.74, 0.06)<br>• Psychological reward: -0.18 (-0.42, 0.07)<br><br>• Mean urges to smoke scores in IQOS, cigarette, and abstinence groups: 4.13, 4.13, and 3.98, respectively
**Findings overview**	• Switching for five days from cigarette smoking to using IQOS reduced exposure to HPHC<br>• Nicotine uptake was similar between IQOS and cigarette group participants<br>• Participants scored IQOS lower on four out of five subjective experience subscales than cigarettes and IQOS was significantly less satisfying than cigarettes
**Authors, study year**	**Haziza et al., 2016** [9]
**Funder/Affiliations**	**Affiliations**: Philip Morris International R&D, Philip Morris Products S.A., Neuchatel, Switzerland
**Primary aim**	To compare exposure to HPHC during 5 days of use
**Products used**	• IQOS<br>• Participants' preferred brand of non-menthol cigarettes
**Methods**	**Design:** Randomised controlled trial<br>**Recruitment:** via the clinical site's database and through advertisements<br>**Study date and Setting:** June–September, 2013, BioVirtus Research Site, Kajetany, Poland<br>**Protocol registered:** 8 October, 2013 (NCT01959932 at clinicaltrials.gov)<br>**Inclusion criteria:** healthy Caucasian smokers; 21–65 years old; smoked ≥10 non-mentholated CPD (max yield of 1 mg nicotine/cig) for the last 4 weeks; had smoked for ≥3 years prior recruitment; not willing to quit smoking in the forthcoming 3 months; ready to accept a 5-day smoking interruption<br>**Exclusion criteria:** participants with clinically relevant medical conditions, those who required medical interventions (start of treatment, surgery or hospitalization), a history of alcohol and/or drug abuse, used nicotine containing products other than their own brand of cigarettes, pregnant or breast feeding, and females not using effective contraception
**Participants**	329 screened; 160/329 (49%) did not meet eligibility criteria; 9/169 (5%) dropped out before randomisation<br>N=160; 50% male; mean age 34.2; mean FTND=5.1<br>Dropped out: 1/80 in IQOS group
**Interventions/Exposure**	Randomised 2:1:1 to IQOS (n=80), cigarette (n=41), and abstinence (n=39) conditions for 5 days in confinement.

- IQOS group participants were asked to *ad libitum* use exclusively IQOS tobacco product
- Cigarette group participants were asked to *ad libitum* use exclusively their own brand of cigarettes
- Abstinence group participants were asked to completely abstain from smoking for five days. The use of nicotine replacement therapy was not allowed

Outcome/Key findings

Levels of biomarkers of exposure to HPHC at day 5 in smoking, abstinence and IQOS groups*

Parent harmful and potentially harmful compound	Geometric mean (95% CI) of exposure levels to HPHC biomarkers		
	**Smoking group**	**Abstinence group**	**IQOS group**
Carbon monoxide (%)	4.51 (4.05; 5.01)	0.99 (0.95; 1.03)	1.06 (1.03; 1.08)
Acrolein (ng/mg creat)	931.01 (825.73; 1049.72)	245.69 (226.15; 266.91)	402.26 (366.55; 441.45)
1,3-butadiene (pg/mg creat)	2399.40 (1884.60; 3054.83)	163.17 (138.41; 192.36)	192.93 (174.90; 212.83)
Benzene (pg/mg creat)	2922.81 (2362.80; 3615.54)	143.70 (122.15; 169.04)	164.45 (144.45; 187.22)
Nicotine-derived nitrosamine ketone (NNK) (pg/mg creat)	107.04 (85.90; 133.37)	41.51 (31.76; 54.26)	49.65 (42.47; 58.05)
Pyrene (pg/mg creat)	182.85 (161.24; 207.37)	85.13 (75.37; 96.15)	81.22 (74.82; 88.16)
N-nitrosonornicotine (NNN) (pg/mg creat)	5.99 (4.94; 7.26)	0.16 (0.14; 0.19)	1.55 (1.17; 2.05)
4-Aminobiphenyl (pg/mg creat)	12.58 (11.03; 14.34)	1.60 (1.40; 1.83)	1.9 (1.70; 2.12)
1-aminonaphthalene (pg/mg creat)	89.37 (77.81; 102.64)	2.56 (2.25; 2.90)	3.30 (2.89; 3.78)
2-aminonaphthalene (pg/mg creat)	25.32 (22.27; 28.79)	2.52 (2.23; 2.84)	2.96 (2.67; 3.28)
o-toluidine (pg/mg creat)	121.16 (105.07; 139.71)	41.64 (36.74; 47.18)	51.15 (46.10; 56.75)
Acrylonitrile (ng/mg creat)	99.48 (85.79; 115.35)	12.6 (10.12; 15.70)	13.18 (11.37; 15.27)
Ethylene oxide (pg/mg creat)	4504.00 (3506.73; 5784.88)	1248.27 (980.62; 1588.98)	1342.40 (1140.44; 1580.12)
Crotonaldehyde (ng/mg creat)	376.78 (329.54; 430.80)	63.25 (57.79; 69.22)	86.65 (80.31; 93.49)
Benzo(a)pyrene (fg/mg creat)	130.29 (110.17; 154.07)	33.64 (28.84; 39.24)	37.07 (33.25; 41.32)
Nicotine equivalents (mg/g creat)	9.76 (8.54; 11.15)	0.14 (0.12; 0.17)	10.60 (9.34; 12.04)
Nicotine (ng/ml)	19.01 (16.52; 21.87)	0.10 (0.09; 0.12)	20.74 (17.46; 24.62)
Cotinine (ng/ml)	219.73 (190.21; 253.83)	2.05 (1.56; 2.67)	239.99 (211.30; 272.58)

* Data from duplicate publication [30]; Note: creat: creatinine

Daily use of IQOS tobacco sticks vs cigarettes

Time	Mean (SD) IQOS tobacco sticks	Mean (SD) cigarettes	% IQOS:Cigarettes
Day 1	14.9 (6.1)	14.5 (3.6)	102.8%
Day 5	20.7 (8.1)	16.6 (3.8)	124.7%

Human puffing topography

- At day 1, in IQOS group compared with cigarette group:

	◦ Average puff duration 26% longer<br>◦ Puff frequency 31% higher<br>• At day 4, in IQOS group compared with cigarette group:<br>◦ Average puff duration 32% longer<br>◦ Puff frequency 32% higher<br><br>**Differences in modified cigarette evaluation subscales' scores (IQOS - cigarette):**<br>• Smoking satisfaction: **-1.26 (-1.68, -0.85)**<br>• Aversion: **0.25 (0.04, 0.46)**<br>• Craving reduction: **-1.12 (-1.58, -0.66)**<br>• Enjoyment of respiratory tract sensation: **-1.0 (-1.36, -0.64)**<br>• Psychological reward: **-0.72 (-1.06, -0.39)**<br><br>• Difference in urges to smoke scores between IQOS and cigarette groups' all time points': -0.3 (-0.75, 0.12)
**Findings overview**	• IQOS provide the same amount of nicotine and suppress urges to smoke similarly to cigarettes<br>• Switching for five days from cigarette smoking to using IQOS reduces exposure to HPHC<br>• Smokers that switched to IQOS used more tobacco sticks than smokers who continued smoking cigarettes, IQOS users show prolonged puff duration and higher puffing frequency<br>• Participants scored IQOS significantly lower on four out of five subjective experience subscales than cigarettes: IQOS was significantly less satisfying, less reducing cravings, less enjoyable in relation to respiratory tract sensation, and less psychologically rewarding than cigarettes
**Authors, study year**	**Ludicke et al., 2016** [6]
**Funder/Affiliations**	**Affiliation:** Department of Research and Development, Philip Morris Products S.A., Neuchâtel, Switzerland
**Primary aim**	To compare exposure to HPHC during 5 days of use
**Products used**	• Carbon heated tobacco product (CHTP)<br>• Participants' own preferred brand of non-menthol cigarettes
**Methods**	**Design:** Randomised controlled trial<br>**Recruitment:** not described<br>**Study date and Setting:** November 2008 – February 2009; MTZ Clinical Research Ltd, Warsaw, Poland<br>**Protocol registered:** 19 December, 2008 (NCT00812279 at clinicaltrials.gov)<br>**Inclusion criteria:** healthy Caucasian smokers; body mass index between 18.5−27.5 kg/m^2; 23−55 years old; 10−30 CPD (tar yield of ≤10 mg/cig); smoking for at least 5 consecutive years<br>**Exclusion criteria:** pregnant or breast feeding females and females not using effective contraception
**Participants**	130 screened; 18/130 (14%) did not meet eligibility criteria<br>N=112; 50% male; mean age 36.3; mean FTND=5.6

	No one dropped out
**Interventions/Exposure**	Randomised 2:1:1 to CHTP (n=56), cigarette (n=28), and abstinence (n=28) conditions for 5 days in confinement<br>• CHTP group participants were asked to *ad libitum* use exclusively carbon heated tobacco product<br>• Cigarette group participants were asked to *ad libitum* use exclusively their own brand of cigarettes<br>• Abstinence group participants were asked to completely abstain from smoking for five days. The use of nicotine replacement therapy was not allowed but they underwent counselling on smoking cessation
**Outcome/Key findings**	See below

Levels of biomarkers of exposure to HPHC at day 5 in smoking, abstinence ad CHTP groups

Parent harmful and potentially harmful compound	Geometric mean (95% CI) of exposure levels to HPHC biomarkers		
	Smoking group	Abstinence group	CHTP group
Carbon monoxide (%)	5.9 (1.3)	1.1 (0.1)	2.3 (0.6)
Acrolein (mg/24 h)	1.9 (0.8)	0.3 (0.1)	0.5 (0.2)
1,3-butadiene (µg/24 h))	7.9 (4.4)	0.8 (0.3)	0.8 (0.3)
Benzene (pg/mg creat)	6.4 (3.1)	0.8 (0.3)	1.0 (0.4)
Nicotine-derived nitrosamine ketone (NNK) (ng/24 h)	279.0 (148.0)	124.5 (94.1)	145.3 (166.1)
Pyrene (ng/24 h)	434.8 (162.1)	237.3 (85.8)	247.5 (113.0)
4-Aminobiphenyl (ng/ 24 h)	21.4 (11.5)	3.7 (2.8)	3.4 (1.6)
2-aminonaphthalene (ng/24 h)	34.0 (16.3)	11. (19.5)	6.4 (8.9)
o-toluidine (ng/24 h)	232.1 (77.6)	110.3 (51.1)	114.0 (144.9)
Nicotine equivalents (ng/ml)	17.2 (5.0)	0.6 (0.3)	19.1 (7.5)
Cotinine (mg/24 h)	289.8 (76.4)	9.4 (5.3)	319.8 (109.7)

Note. creat: creatinine

Daily use of CHTP vs cigarettes

Time	Mean (SD) CHTP	Mean (SD) cigarettes	% CHTP:Cigarettes
Day 1	17.8 (3.2)	17.4 (3.4)	102.3%
Day 5	19.7 (7.8)	18.8 (4.4)	104.8%

Human puffing topography

- At day 1, CHTP % changes compared with baseline (measured while using own brand of cigarettes):
    - 20% longer puff duration
    - 45% higher average puff volume
    - 59% more frequent puffs
    - 125% higher total puff volume
- At day 5, CHTP % changes compared with baseline (measured while using own brand of cigarettes):
    - 23% longer puff duration

	○ 51% higher average puff volume<br>○ 70% more frequent puffs<br>○ 148% higher total puff volume
**Findings overview**	• Switching for five days from cigarette smoking to using carbon heated tobacco product use reduces exposure to HPHC<br>• Smokers who switched to using CHTP employed compensatory puffing behaviour and used slightly more of the product than had used cigarettes at baseline<br>• Yielded nicotine levels were comparable between CHTP and cigarette groups
**Authors, study year**	**Ludicke et al., 2017** [5]
**Funder/Affiliations**	**Affiliations:** Philip Morris Products S.A., Research & Development, Neuchâtel, Switzerland
**Primary aim**	To compare exposure to HPHC during 5 days of use
**Products used**	• Tobacco heating system 2.1 (THS 2.1)<br>• Participants' preferred brand of non-menthol cigarettes
**Methods**	**Design:** Randomised controlled trial<br>**Recruitment:** via the clinical site's database and through advertisements<br>**Study date and Setting:** June–July 2012; Poland<br>**Protocol registered:** 15 January, 2013 (NCT01780714 at clinicaltrials.gov)<br>**Inclusion criteria:** 23−65 years old; smokers of ≥10 CPD (nicotine ≤1 mg/cig) for 4 weeks prior start of the study<br>**Exclusion criteria:** smoking menthol cigarettes
**Participants**	42 screened; 2/42 (5%) were not randomised and were treated as back-up participants<br>N=40; 53% female; mean age 37,7; mean FTND=6.3<br>No one dropped out
**Interventions/Exposure**	Randomised 1:1 to THS 2.1 (n=20) and cigarette (n=20) conditions for 5 days in confinement<br>• THS 2.1 group participants were asked to *ad libitum* use exclusively THS 2.1 tobacco product<br>• Cigarette group participants were asked to *ad libitum* use exclusively own brand of cigarettes
**Outcome/Key findings**	**Levels of biomarkers of exposure to HPHC at day 5 in THS 2.1 and smoking groups** (see table below)

Levels of biomarkers of exposure to HPHC at day 5 in THS 2.1 and smoking groups

**Parent harmful and potentially harmful compound**	**Geometric mean (95% CI) of exposure levels to HPHC biomarkers**	
	**Smoking group**	**THS 2.1 group**
Carbon monoxide (%)	5.86 (5.25; 6.54)	1.37 (1.30; 1.45)
Acrolein (µg/g creat)	1227.45 (1023.62; 1471.86)	327.31 (288.40, 371.46)
1,3-butadiene (µg/g creat)	3.233 (2.31; 4.51)	0.352 (0.26; 0.47)
Benzene (µg/g creat)	4.49 (3.25; 6.21)	0.3 (0.21; 0.42)
Nicotine-derived nitrosamine ketone (NNK) (ng/g creat)	186.8 (138.51; 251.91)	55.9 (36.95; 84.56)
Pyrene (µg/g creat)	187.84 (155.69; 226.62)	85.81 (73.65; 99.96)
N-nitrosonornicotine (NNN) (ng/g creat)	6.45 (4.76; 8.73)	0.806 (0.61; 1.06)

4-Aminobiphenyl (ng/g creat)	24.67 (18.74; 32.48)	9.88 (8.01; 12.18)
2-aminonaphthalene (ng/g creat)	99.72 (81.94; 121.37)	10.40 (8.26; 13.09)
o-toluidine (ng/g creat)	284.16 (247.83; 325.80)	157.82 (129.47; 192.38)
Acrylonitrile (ng/g creat)	149.80 (121.83; 184.19)	18.15 (13.50; 24.38)
Nicotine equivalents (mg/g creat)	13.47 (11.50; 15.77)	11.12 (8.96; 13.80)
Nicotine (ng/ml)	17.07 (14.34; 20.30)	14.16 (10.27; 19.51)
Cotinine (ng/ml)	265.52 (231.16; 304.98)	236.15 (190.42; 292.86)

Note. creat: creatinine

Daily use of THS 2.1 vs cigarettes

Time	Mean (SD) THS 2.1	Mean (SD) Cigarettes	% THS 2.1:Cigarettes
Day 1	21.4 (7.4)	17.8 (3.0)	120.2%
Day 5	27.2 (9.1)	20.1 (3.2)	135.3%

Human puffing topography

- At day 1, THS 2.1 group changes compared with cigarette group:
  - Puff duration 19% longer
  - Inter-puff interval 39% shorter
  - Puff volume 14% higher
  - Total volume 21% higher
- At day 4, THS 2.1 group changes compared with cigarette group:
  - Puff duration 35% longer
  - Inter-puff interval 39% shorter
  - Puff volume 12% higher
  - Total volume 10% higher

Modified cigarette evaluation subscales' scores (THS 2.1 vs cigarette):

Subjective effects of smoking subscales	Day1		Day 5	
	THS 2.1 Mean (95% CI)	Cigarettes Mean (95% CI)	THS 2.1 Mean (95% CI)	Cigarettes Mean (95% CI)
Smoking satisfaction	**2.7 (2–3.3)**	**4.6 (4.1–5.2)**	**3.4 (2.8–3.9)**	**4.8 (4.1–5.5)**
Psychological rewards	**2.3 (1.9–2.8)**	**3.5 (2.8–4.1)**	**2.6 (2.0–3.2)**	**3.1 (2.4–3.8)**
Enjoyment of respiratory tract sensation	**2.1 (1.4–2.8)**	**3.6 (2.7–4.5)**	**2.3 (1.6–3.0)**	**3.9 (3.0–4.7)**
Craving reduction	**3.1 (2.3–3.9)**	**5.0 (4.3–5.6)**	**3.3 (2.5–4.1)**	**4.7 (3.9–5.4)**
Aversion	1.4 (1.0–1.7)	1.2 (0.9–1.5)	1.1 (0.9–1.2)	1.2 (0.8–1.5)

	**Bolded** are the statistically significant differences between two participants' groups
**Findings overview**	• THS 2.1 provides the similar amount of nicotine compared with smoking cigarettes<br>• Switching for five days from cigarette smoking to using THS 2.1 reduces exposure to HPHC<br>• Smokers that switched to THS 2.1 used more tobacco sticks than smokers who continued smoking cigarettes, THS 2.1 users showed prolonged puff duration, increased puffing volume and puffing frequency<br>• THS 2.1 was perceived as significantly less satisfying, less reducing cravings, less enjoyable in relation to respiratory tract sensation, and less psychologically rewarding than cigarettes
**Authors, study year**	**Picavet et al., 2016** [2]
**Funder/Affiliations**	**Affiliations:** Department of Research and Development, Philip Morris Products S.A., Neuchâtel, Switzerland
**Primary aim**	To compare nicotine delivery and effects on urge to smoke
**Products used**	• THS 2.1<br>• Cigarettes
**Methods**	**Design:** Randomised crossover experimental trial<br>**Recruitment:** via the clinical site's database and by advertisements<br>**Study date and setting:** May–June 2012; Celerion GB Ltd, Northern Ireland, United Kingdom<br>**Protocol registered:** 15 January, 2013 (NCT01780688 at clinicaltrials.gov)<br>**Inclusion criteria:** healthy Caucasian smokers; 23−65 years old; smokers of ≥10 non-menthol CPD (nicotine ≤1 mg/cig) for 4 weeks prior start of the study; cigarette smokers for ≥3 years before screening<br>**Exclusion criteria:** a body mass index of less than 18.5 or more than 30 kg/m^2; a urinary cotinine level less than 200 ng/mL at screening; smoker of hand-rolled cigarettes, cigars, pipes, bidis, or other non-eligible nicotine-containing products, including electronic cigarettes; unable to abstain from smoking for up to 2 consecutive days; having clinically relevant diseases or a medical condition requiring smoking cessation
**Participants**	Information about screened or excluded participants is not reported<br>N=28; 50% male; mean age 29.6; mean FTND=4.9<br>No dropouts
**Interventions/Exposure**	Randomised 1:1 to THS 2.1 use crossover to cigarettes (n=14) and cigarette use crossover to THS 2.1 (n=14) conditions for 7 days in confinement:<br>• Sequence 1: 24-hour nicotine wash-out period, a day of single THS 2.1 use, a day of *ad libitum* THS 2.1 use, 24-hour nicotine wash-out period, a day of single cigarette use, a day of *ad libitum* cigarette use<br>• Sequence 2: 24-hour nicotine wash-out period, a day of single cigarette use, a day of *ad libitum* cigarette use, 24-hour nicotine wash-out period, a day of single THS 2.1 use, a day of *ad libitum* THS 2.1 use
**Outcome/Key findings**	**Nicotine concentration pharmacokinetics of tobacco sticks in comparison with cigarettes**

**Single use**	**THS 2.1, mean (95% CI)**	**Cigarette, mean (95% CI)**	**THS 2.1 : Cigarette ratio (90% CI)**
$AUC_{0\text{–}last}$ (ng h/ml)	17.7 (15.0, 20.8)	22.8 (19.4, 26.8)	77.4% (70.5–85.0)

C_{max} (ng/ml)	8.4 (6.8, 10.3)	11.9 (9.5, 14.9)	70.3% (60.0–82.2)
t_{max} (min)	Median = 8	Median = 8	Median diff: <0.1 (-1.0–2.0)
$t_{1/2}$ (h)	2.6 (2.3, 3.0)	2.5 (2.2, 2.8)	110.9% (101.7–120.9)
***Ad libitum* use**			
C_{peak} (ng/ml)	14.9 (12.3, 18.1)	24.0 (21.7, 26.6)	62.0% (53.6–71.8%)
C_{trough} (ng/ml)	4.1 (2.4, 7.0)	12.3 (10.4, 14.6)	33.5% (21.9–51.2)
t_{peak} (h)	Median = 12.9	Median = 10.5	Median diff: 1.6 (0.0, 2.4)
Times used per day	10.9 (SD=3.6)	16.7 (SD=3.5)	65.3%

Note: $AUC_{0\text{-}last}$: area under plasma concentration-time curve from start of product use extrapolated to the last measurable concentration; C_{max}: maximum observed plasma concentration; t_{max}: time to maximum plasma concentration; $t_{1/2}$: terminal elimination half-life; C_{peak}: maximum observed plasma concentration; C_{trough}: lowest observed plasma concentration during the same sampling interval in which C_{peak} was observed; t_{peak}: time to the maximum observed concentration

Urges to smoke (QSU-brief) scores:

- After single use: similar transient reduction for both THS 2.1 and cigarette use (−19.4 ± 22.4 vs −19.5 ± 23.1, respectively)
- Following *ad libitum* use: for the THS 2.1 and cigarette the overall mean difference for the total score was 1.4 (95% CI: −1.0–3.7)

Cough assessment: no apparent differences for cough frequency, cough intensity, or sputum production between the study groups

Modified cigarette evaluation subscales' scores (THS 2.1 vs cigarette):

Subjective effects of smoking subscales	Single use		*Ad libitum* use	
	THS 2.1 Mean (SD)	Cigarettes Mean (SD)	THS 2.1 Mean (SD)	Cigarettes Mean (SD)
Smoking satisfaction	4.1 (1.5)	4.6 (1.9)	**3.3 (1.4)**	**5.2 (1.2)**
Psychological rewards	3.9 (1.2)	3.7 (1.4)	**3.3 (1.5)**	**4.1 (1.3)**
Enjoyment of respiratory tract sensation	**3.6 (1.4)**	**4.3 (1.8)**	**2.6 (1.5)**	**4.6 (1.6)**
Craving reduction	4.7 (1.6)	4.6 (1.7)	**3.9 (1.9)**	**5.4 (1.3)**
Aversion	**2.2 (1.5)**	**3.0 (1.8)**	1.8 (1.1)	1.7 (1.1)

Bolded are the statistically significant differences between two participants' groups

Findings overview

- THS 2.1 provided lower exposure to nicotine compared with cigarettes both after single use and following *ad libitum* use
- Following *ad libitum use* cigarette users had significantly higher peak and trough plasma nicotine levels than THS 2.1 users
- Both cigarettes and THS 2.1 reduced urges to smoke similarly
- THS 2.1 was perceived as less significantly satisfying, less reducing cravings, less enjoyable in relation to respiratory tract sensation, and less psychologically rewarding after *ad libitum* use than cigarettes

**Authors, study year**	**Ludicke et al., 2017 & Ludicke et al., 2017** [10,Ludicke, 2017 #89]}
**Funder/Affiliations**	**Affiliations:** Philip Morris Products S.A., PMI Research and Development, Neuchâtel, Switzerland
**Primary aim**	• Part 1: To compare exposure to HPHC during 5 days of use in confinement and further 85 days of use in an ambulatory setting<br>• Part 2: To compare effect on biologically and clinically relevant risk markers during 90 days of use
**Products used**	• IQOS with menthol tobacco sticks<br>• Cigarettes: participants' preferred brand of menthol cigarettes
**Methods**	**Design:** Randomised controlled trial<br>**Recruitment:** via the clinical site's database and by advertisements<br>**Study date and setting:** July 2013; Tokyo Heart Center Osaki Hospital, Japan<br>**Protocol registered:** 18 October, 2013 (NCT01970995 at clinicaltrials.org)<br>**Inclusion criteria:** healthy Japanese smokers; 23–65 years old; a body mass index of 18.5–32 kg/m^2; smokers of ≥10 menthol CPD (nicotine ≤1 mg/cig) for 4 weeks prior start of the study; menthol cigarette smokers for ≥3 years before screening; do not plan to quit smoking in the next 3 months; ready to stop smoking for up to 90 days; ready to use the menthol IQOS tobacco sticks<br>**Exclusion criteria:** any medical, psychiatric, and/or social reason; legally incompetent, physically or mentally incapable of giving consent; medical condition requiring smoking cessation; use of nicotine-containing products other than menthol cigarettes or electronic cigarettes/similar devices within 4 weeks prior to enrolment; administration of drugs likely to affect CYP1A2 or CYP2A6 activity within 14 days or five half-lives of the drug 2 days before randomisation; administration of drugs within 14 days of Day 2 that were likely to interfere with the study objectives or the participant's safety; concomitant use of non-steroidal anti-inflammatory drugs (NSAIDs) or acetylsalicylic acid; positive alcohol test and/or history of alcohol abuse; positive urine drug test; positive serology test for human immunodeficiency virus, hepatitis B, or hepatitis C virus; donation/receipt of whole blood/blood products within 3 months prior to admission; current or former employee of the tobacco industry, or of their first-degree relatives (parent, sibling, or child); employee of the investigational site or of their first-degree relatives; participation in a clinical study within 3 months before screening; participation in the same study at a different time; pregnant/breast feeding; women not using effective contraception
**Participants**	670 participants were screened; 454/670 (67.8%) were accepted before randomisation; 56/216 (25.9%) were not randomised<br>N=160; 57.5% male; mean age 37.2 ± 10.5; mean FTND=4.4 ± 1.9<br>Dropped-out: IQOS group=2; cigarette group=1; abstinence group=2
**Interventions/Exposure**	Randomised 2:1:1 to IQOS (n=78), menthol cigarette (n=42), and abstinence (n=40) conditions<br>• For first two baseline days in confinement participants smoked *ad libitum* their menthol cigarettes<br>• Then, for five days IQOS group *ad libitum* used menthol IQOS tobacco sticks, cigarette group continued to smoke their preferred menthol tobacco cigarettes, and abstinence group abstained from smoking. The abstinence group were provided with psychological support and the use of IQOS was strictly forbidden for the whole study duration (use of menthol or other cigarettes was not explicitly forbidden)<br>• During the 85-day ambulatory period, the participants returned to the study site and stayed overnight on the days 30, 60, and 90 visits. In the IQOS group during the ambulatory period dual use of IQOS and menthol cigarettes was possible. In the menthol cigarette and abstinence groups the use of IQOS was strictly forbidden. The use of nicotine replacement therapy was allowed during the ambulatory period in the abstinence group
**Outcome/Key findings**	• Part 1

**Levels of biomarkers of exposure to HPHC at day 5 and day 90 in menthol IQOS, menthol cigarette and abstinence groups**						
**Parent harmful and potentially harmful compound**	**Geometric mean (95% CI) of exposure levels to HPHC biomarkers**					
	**Day 5**			**Day 90**		
	**IQOS group**	**Smoking group**	**Abstinence group**	**IQOS group**	**Smoking group**	**Abstinence group**
Carbon monoxide (%)	2.48 (2.40, 2.57)	5.55 (5.06, 6.08)	2.50 (2.38, 2.64)	2.97 (2.88, 3.06)	5.73 (5.24, 6.25)	3.04 (2.84, 3.26)
Acrolein (ng/mg creat)	304.68 (284.63, 326.14)	591.33 (507.72, 688.69)	186.71 (163.39, 213.36)	386.37 (356.30, 418.97)	695.58 (602.43, 803.13)	276.13 (242.11, 314.93)
1,3-butadiene (pg/mg creat)	81.71 (75.52, 88.41)	622.58 (454.60, 852.64)	80.72 (70.92, 91.88)	141.74 (120.62, 166.57)	785.27 (576.82, 1069.04)	136.83 (114.40, 163.66)
Benzene (pg/mg creat)	118.36 (107.37, 130.48)	1096.47 (805.13, 1493.22)	102.51 (85.19, 123.34)	145.58 (121.67, 174.18)	1157.25 (848.59, 1578.17)	144.07 (109.87, 188.92)
Nicotine-derived nitrosamine ketone (NNK) (pg/mg creat)	37.90 (32.29, 44.48)	85.94 (70.93, 104.13)	29.58 (22.24, 39.35)	23.23 (19.34, 27.91)	95.03 (77.31, 116.82)	13.95 (9.00, 21.60)
Pyrene (pg/mg creat)	46.36 (41.68, 51.55)	122.90 (104.71, 144.26)	41.14 (35.42, 47.78)	85.47 (76.64, 95.33)	167.38 (146.23, 191.58)	88.21 (75.53, 103.01)
N-nitrosonornicotine (NNN) (pg/mg creat)	1.20 (0.97, 1.49)	4.10 (2.94, 5.73)	0.15 (0.12, 0.18)	1.40 (1.13, 1.73)	4.28 (3.03, 6.05)	0.26 (0.17, 0.40)
4-Aminobiphenyl (pg/mg creat)	1.97 (1.76, 2.21)	9.50 (8.15, 11.07)	2.16 (1.87, 2.50)	2.07 (1.82, 2.36)	9.62 (8.12, 11.39)	2.35 (1.90, 2.89)
1-aminonaphthalene (pg/mg creat)	3.14 (2.85, 3.46)	53.27 (45.86, 61.89)	2.85 (2.50, 3.26)	3.55 (2.96, 4.26)	55.34 (46.21, 66.26)	4.22 (3.20, 5.55)
2-aminonaphthalene (pg/mg creat)	1.97 (1.80, 2.15)	14.23 (12.18, 16.62)	2.04 (1.82, 2.28)	2.34 (2.11, 2.59)	14.84 (12.63, 17.44)	2.63 (2.20, 3.15)
o-toluidine (pg/mg creat)	51.64 (45.52, 58.59)	127.28 (103.27, 156.88)	48.82 (40.94, 58.21)	68.35 (53.91, 86.67)	125.64 (96.13, 164.20)	77.86 (56.72, 106.88)
Acrylonitrile (ng/mg creat)	12.43 (11.12, 13.90)	68.17 (56.39, 82.40)	11.78 (9.84, 14.10)	7.91 (6.74, 9.29)	83.98 (69.17, 101.95)	8.41 (5.99, 11.81)
Ethylene oxide (pg/mg creat)	1137.96 (995.5, 1300.81)	2235.37 (1742.88, 2867.03)	1113.73 (923.72, 1342.83)	1741.53 (1510.19, 2008.3)	3739.46 (2858.39, 4892.12)	1633.12 (1286.77, 2072.69)
Crotonaldehyde (ng/mg creat)	124.47 (115.36, 134.30)	286.80 (251.37, 327.21)	113.48 (99.38, 129.59)	154.30 (137.07, 173.70)	299.41 (260.62, 343.97)	158.57 (132.95, 189.14)
Benzo(a)pyrene	20.72 (18.61,	75.10 (62.60,	17.84 (15.45,	30.02 (25.29,	86.92 (71.78,	28.88 (22.56,

(fg/mg creat)	23.07)	90.08)	20.58)	35.65)	105.27)	36.98)
Nicotine equivalents (mg/g creat)	6.16 (5.55, 6.83)	5.22 (4.35, 6.27)	0.16 (0.12, 0.20)	6.85 (5.96, 7.88)	6.33 (5.11, 7.84)	0.37 (0.18, 0.78)

Note: creat: creatinine.

Daily use of IQOS menthol tobacco sticks and menthol cigarettes

Time	Mean (SD) menthol IQOS	Mean (SD) menthol cigarettes	% IQOS:Cigarettes
Day 1	11.4 (3.9)	11.0 (4.0)	103.6%
Day 5	13.9 (4.3)	13.6 (4.7)	102.2%
Days 6–30	11.7 (6.0)	13.8 (4.2)	84.8%
Days 30–60	12.7 (6.3)	14.9 (5.7)	85.2%
Days 60–90	12.7 (6.5)	15.2 (5.0)	83.6%

Human puffing topography (results in figures only, summary is based on authors' verbatim presentation of results)

- During confinement period:
    - Total smoking duration: decreased in IQOS and were stable in menthol cigarette group
    - Total number of puffs: at baseline IQOS group > menthol cigarette group, stable in both groups during confinement
    - Average puff interval: decreased in IQOS group and remained stable in menthol cigarette group
- During ambulatory period:
    - Total smoking duration: decreased in both groups
    - Total number of puffs: IQOS group > menthol cigarette group on day 90
    - Average puff interval: IQOS group < menthol cigarette group
    - Total puff volume: comparable in IQOS and menthol cigarette groups
    - Average puff volume: IQOS < menthol cigarette group

Subjective effects of smoking (results in figures only; summary is based on authors' verbatim presentation of results)

- The IQOS scores (modified cigarette evaluation questionnaire) for the Craving Reduction, Enjoyment of Respiratory Tract Sensations, Psychological Reward, and Smoking Satisfaction subscales were lower in the IQOS group than in the menthol cigarette group from days 1 until 30. There was a negligible difference in the aversion subscale.
- From day 30 onwards, the subscale scores were comparable between the IQOS and menthol cigarette groups.

Urges to smoke (QSU-brief) questionnaire

- The QSU-brief total scores remained fairly stable in the IQOS and menthol cigarette groups throughout the confinement and ambulatory periods, albeit the scores were slightly higher in the IQOS group.

Part 2

	**Changes in risk markers at day 90: least squares (LS) mean ratio (IQOS : menthol cigarette) in % (95% CI; *p*)**<br>• Endothelial dysfunction: soluble intercellular adhesion molecule-1 (sICAM-1; ng/ml) = 91.28% (85.06–97.95; p=.0116)<br>• Oxidative stress: 8-epi-prostaglandin F2α (8-epi-PGF2α; pg/mg creat) = 87.29% (78.19–97.45; p=.0159)<br>• Platelet activation 11-dehydro-thromboxane B2 (11-DTX-B2; pg/mg creat) = 91.02% (80.48–102.94; p=.1327)<br>• Cardiovascular risk factors:<br>• Fibrinogen (mg/dL) = 94.58% (87.87–101.8; p=.136)<br>• Homocysteine (µmol/L) = 100.66% (93.35–108.54; p=.8638)<br>• High-sensitivity C-reactive protein (hs-CRP) (mg/L) = 93.59% (62.23–140.75; p=.7487)<br>• Metabolic syndrome: Glucose (mg/dL) = 98.8% (96.42–101.6; p=.437)<br><br>**Changes in risk markers at day 90: least squares (LS) mean difference (IQOS : menthol cigarette) (95% CI; *p*); proportion (%) of IQOS and menthol cigarette groups' arithmetic means**<br>• Inflammation: white blood cell count (WBC) (GI/L) = -0.57 (-1.03, -0.1; p=0.0173); 91.7%<br>• Lipid metabolism:<br>• Low-density lipoprotein (LDL) cholesterol (mg/dL) = 0.9 (-6.6, 8.3; p=.8162); 99.4%<br>• High-density lipoprotein (HDL) cholesterol (mg/dL) = 4.5 (1.1, 7.9; p=.0084); 103.1%<br>• Triglycerides (mg/dL) = -6.3 (-21.2, 8.7; p=.4095); 100.9%<br>• Total cholesterol (mg/dL) = 2.0 (-6.7, 10.7; p=.6499); 99.5%<br>• Metabolic syndrome<br>• Hemoglobin A1c (%) = 0.02 (-0.06, 0.1; p=.5866); 99.4%<br>• Body weight (kg) = -0.09 (-0.75, 0.57; p=.7926); 100.4%<br>• Waist circumference (cm) = 1.6 (-2.4, 5.6; p=.4251); 101.0%<br>• Cardiovascular risk factors<br>• Systolic blood pressure (mmHg) = -0.59 (-3.8, 2.62; p=.7157); 98.8%<br>• Diastolic blood pressure (mmHg) = -0.68 (-3.04, 1.69; p=.5705); 98.3%<br>• Lung function: Forced expiratory volume in 1 second (% of those predicted) = 1.91 (-0.14, 3.97; p=.0669); 101.6%
**Findings overview**	• Switching from menthol cigarette use to menthol IQOS use reduced exposure to HPHC after five days in confinement and to a lesser extent after further 85 days throughout the ambulatory setting<br>• Use of IQOS provided similar level of nicotine as smoking menthol cigarettes<br>• Smaller and more frequent puffs with a shorter inter-puff interval and a lower average puff volume were taken with the IQOS than with menthol cigarettes<br>• IQOS group on average used similar number of tobacco sticks per day during confinement as menthol cigarette smokers but less tobacco sticks throughout ambulatory period compared with menthol cigarette smokers<br>• Participants rated menthol IQOS lower on four out of five subjective experience subscales than menthol cigarettes, these scores balanced after 25 days in ambulatory settings

	• Switching from smoking menthol cigarettes to using menthol IQOS was associated with improvement in risk markers linked to oxidative stress, endothelial dysfunction, lipid metabolism, inflammation, and lung function<br>• Authors did not ascertain what part of abstinence group was still abstinent at 90-days follow-up
**Authors, study year**	**Gee et al., 2017** [12]
**Funder/Affiliations**	**Affiliations:** British American Tobacco, Group Research and Development, Regents Park Road, Southampton, SO15 8TL, UK
**Primary aim**	To compare the puffing topography, mouth level exposure, and average daily consumption
**Products used**	• Glo HnB product: with Bright Tobacco Kent Neostiks and mentholated Intensely Fresh Kent Neostiks (Japan)<br>• Cigarettes: according to participants' preferred type, either Lucky Strike Regular (7 mg tar ISO) or Lucky Strike Menthol (7 mg tar ISO) (Japan)<br>• IQOS: with Essence tobacco sticks (Japan)
**Methods**	**Design:** Randomised crossover experimental trial<br>**Recruitment:** participants were recruited by a market research agency.<br>**Study time and setting:** 2016, Tokyo, Japan<br>**Protocol registered:** not reported<br>**Inclusion criteria:** Adult Japanese smokers naïve to heat-not-burn products, between 21 years and 7 months and 64 years of age, smokers of 5 or more menthol and non-menthol cigarettes per day (7–8 mg tar yield ISO) or users of IQOS for five or more sessions per day for a minimum of 3 months, including dual IQOS and cigarette users.<br>**Exclusion criteria:** possibility of pregnancy.<br>**Measures:** Natural puffing topography, mouth level exposure to tar and nicotine, and average daily consumption of test products
**Participants**	Numbers of screened and excluded participants not reported<br>N=208, 52% female, mostly from 30–44 years old age group (52%).
**Interventions/Exposure**	Randomised 1:1:1:1 to:<br>• Group 1: three non-mentholated products randomly provided for 4-days familiarisation periods with Lucky Strike Regular cigarettes, glo with Bright Tobacco Kent Neostiks, and IQOS with Essence tobacco sticks.<br>• Group 2: two mentholated products randomly provided for 4-days familiarisation periods with Lucky Strike Menthol and glo with mentholated Intensely Fresh Kent Neostiks<br>• Group 3: two heat-not-burn products randomly provided for 4-days familiarisation periods with glo with Bright Tobacco Kent Neostiks and IQOS with Essence tobacco sticks.<br>• Group 4: completed a glo use session with regular tobacco sticks to assess mouth insertion depth.<br><br>• Participants in groups 1–3 during the product familiarisation periods were asked to replace their regularly used cigarettes with provided test products and record their consumption in their diary<br>• On day 5 of each product placement period, the participants attended the central location where their puffing topography was measured with the SA7 puffing topography device. The puffing topography was measured and recorded in duplicate for each study product with a minimum of a 20 minute break in-between sessions.

Outcome/Key findings

Puffing topography and daily consumption of cigarettes, glo and IQOS products

	Group 1			Group 2		Group 3	
**Puffing topography measure**	**Regular cigarette Mean ±SD**	**Regular glo Mean ±SD**	**Regular IQOS Mean ±SD**	**Menthol cigarette Mean ± SD**	**Menthol glo Mean ± SD**	**Regular glo Mean ± SD**	**Regular IQOS Mean ± SD**
Total puff volume (ml)	**489.0 ± 177.7**	**736.4 ± 415.8**	668.1 ± 322.6	493.7 ± 192.4	618.2 ± 389.6	773.5 ± 545.7	588.0 ± 360.0
Mean puff volume (ml)	48.9 ± 14.8	**66.7 ± 23.7**	63.5 ± 20.3	**51.1 ± 16.0**	**62.2 ± 32.8**	**60.9 ± 24.8**	**55.1 ± 23.9**
Number of puffs (n/stick)	10.7 ± 5.0	10.9 ± 5.6	10.3. ± 3.6	10.0 ± 3.7	10.0 ± 4.5	12.3 ± 7.3	10.8 ± 5.1
Mean puff duration (s)	1.8 ± 0.6	1.8 ± 0.6	1.8 ± 0.6	2.0 ± 0.5	1.8 ± 0.5	1.8 ± 0.7	1.8 ± 0.7
Mean puff interval (s)	**9.7 ± 3.4**	7.4 ± 2.7	8.3 ± 3.0	**9.9 ± 3.4**	**8.1 ± 3.0**	7.7 ± 3.9	8.6 ± 3.1
Average daily consumption	**16.3 ± 7.9**	**12.1 ± 5.5**	**13.7 ± 5.6**	**15.6 ± 6.9**	**13.1 ± 6.0**	**11.2 ± 6.2**	**13.4 ± 7.8**

Bolded are the cells that differ statistically significantly from other groups

Authors' conclusions

- In general, total and mean puff volumes were larger for glo than for cigarettes
- Puff intervals were shortest for glo tobacco product use
- There was lack of difference in puffing behaviour between naïve glo users and regular IQOS users (except for mean puff volume) which suggests that a familiarisation of 4 days is sufficient
- Mean mouth insertion depth between users of glo was 7.7 mm, which suggests that the air inlet zone was not blocked by the users

**Authors, study year**	**Yuki et al., 2017** [4]
**Funder/Affiliations**	**Affiliations:** Scientific Product Assessment Centre, R&D Group, Japan Tobacco Inc., Japan
**Primary aim**	To compare the pharmacokinetics of nicotine delivery
**Products used**	• Prototype novel tobacco vapour (PNTV) product<br>• Cigarettes
**Methods**	**Design:** Randomised crossover experimental trial<br>**Recruitment:** the recruitment procedure is not described<br>**Study time and setting:** time is not reported, **s**ingle centre in Japan<br>**Protocol registered:** not reported<br>**Inclusion criteria:** Healthy Japanese adult male smokers aged 21–65 years, smoked an average of 11 or more cigarettes per day, and had smoked for at least 12 months before entering the trial.<br>**Exclusion criteria:** body mass index less than 18.5 or more than 25 kg/m^2, urinary cotinine level less than 200 ng/ml at screening, had used any

<table>
<tr><td></td><td>prescription smoking cessation treatment within last 4 weeks before entering the study
Measures: the mouth level exposure of nicotine in cigarette smoking, analysis of the amount of nicotine delivered in the aerosol of PNTV, pharmacokinetics of the tested products was measured by collecting blood samples for plasma nicotine analysis</td></tr>
<tr><td>**Participants**</td><td>Numbers of screened and excluded participants not reported
N=24 (all completed the study), mean age 39 years (range: 21–63), mean tar value of subjects' usual brand of cigarettes 8.8 mg (range: 1–18 mg), mean daily cigarette consumption 18.1 cigarettes (range: 12–30), mean smoking history 18.9 years (range: 1–43 years).</td></tr>
<tr><td>**Interventions/Exposure**</td><td>**Procedure:** on Day 1 subjects checked in to a clinic and abstained from tobacco use. On days 2–3 participants used a PNTV or smoked a single cigarette under controlled use (10 puffs for 3 minutes at approximately 20 seconds intervals). On day 4 participants were discharged.</td></tr>
<tr><td>**Outcome/Key findings**</td><td>**Nicotine delivery pharmacokinetics of PNTV single use in comparison with cigarettes**
<table>
<tr><th></th><th>PNTV, mean (95% CI)</th><th>Cigarette, mean (95% CI)</th><th>PNTV : Cigarette ratio (95% CI)</th></tr>
<tr><td>Mouth level of nicotine exposure (mg)</td><td>**Median=0.355 (range: 0.180–0.580)**</td><td>**Median=0.540 (range: 0.310–0.940)**</td><td>**65.7%**</td></tr>
<tr><td>$AUC_{0–last}$ (ng h/ml)</td><td>**4.12 (3.43, 4.95)**</td><td>**6.03 (5.02, 7.25)**</td><td>**68.3% (54.3%, 85.9%)**</td></tr>
<tr><td>C_{max} (ng/ml)</td><td>**5.39 (4.34, 6.69)**</td><td>**11.8 (9.49, 14.6)**</td><td>**45.7% (34.1%, 61.4%)**</td></tr>
<tr><td>t_{mac} (min)</td><td>Median=3.83 (range: 2.83–7.83)</td><td>Median=3.83 (range: 2.83–4.83)</td><td>100%</td></tr>
<tr><td>$t_{1/2}$ (h)</td><td>1.66 (1.41, 1.95)</td><td>1.86 (1.58, 2.19)</td><td>89.1% (78.2%, 102%)</td></tr>
</table>
Note: **Bolded** are statistically significant differences between tested products; AUC_{0-last}: area under plasma concentration-time curve from start of product use extrapolated to the last measurable concentration; C_{max}: maximum observed plasma concentration; t_{max}: time to maximum plasma concentration; $t_{1/2}$: terminal elimination half-life.</td></tr>
<tr><td>**Authors' conclusions**</td><td>• As there was no significant difference in time to maximum nicotine plasma concentration, PNTV product seems to deliver nicotine via similar absorption sites as cigarettes
• Mouth level exposure to nicotine, maximum observed nicotine plasma concentration and exposure to nicotine after single use of the tested products were significantly lower for PNTV product in comparison with use of a single cigarette. PNTV product provided less nicotine than a cigarette following controlled use</td></tr>
</table>

Table A4 Findings of epidemiology studies on heat not burn use

**Authors, study year**	**Tabuchi et al., 2016** [31]
**Funder/Affiliations**	**Affiliations:**<br>• Center for Cancer Control and Statistics, Osaka Medical Center for Cancer and Cardiovascular Diseases, Osaka, Japan<br>• Department of Public Health, Tokyo Women's Medical University, Tokyo, Japan<br>• Department of Economics, Keio University, Tokyo, Japan<br>• Department of Environmental Health, National Institute of Public Health, Saitama, Japan<br>**Funding:**<br>• Ministry of Health, Labour and Welfare: Comprehensive Research on Lifestyle Related Diseases including Cardiovascular Diseases and Diabetes Mellitus (H25-010 and H26-023)
**Primary aim**	To report awareness and use of HnB products in a nationally representative sample
**Products used**	• IQOS<br>• Ploom/Ploom TECH<br>• glo
**Methods**	**Design:** Epidemiological study<br>**Data collection:** Internet survey inviting participants from a large survey panel managed by a internet research agency Rakuten Research<br>**Study time:** 31st January–17th February 2015<br>**Sampling frame:** defined by the Census in Japan<br>**Measures:** awareness and use of e-cigarettes and heat not burn tobacco products, smoking status, other variables (residence area, marital status, education, housing tenure, occupation, self-rated health)
**Participants**	Participation rate: 8.5% (9055/106202)<br>N=8240 (after excluding participants with discrepancies in reported data)
**Key findings**	• 48.0% (95% CI: 46.9–49.1) were aware of e-cigarettes and HnB tobacco products<br>• Current smokers (66–68%) were more aware of e-cigarettes and HnB than never smokers (37–44%)<br>• 6.6% (95% CI: 6.06–7.13) had ever used e-cigarettes or HnB<br>• Among those who had ever used e-cigarettes or HnB, 7.8% (or 0.5148% in total) had ever used Ploom and 8.4% (or 0.5544% in total) had ever used IQOS
**Authors' conclusions**	• Approximately half of Japanese aged 15–69 were aware of e-cigarettes and HnB tobacco products, 6.6% had ever used, 1.3% used in the last 30 days and 1.3% had more than 50 sessions of ever use<br>• Among ever users of e-cigarettes and HnB, 7.8% and 8.4% used Ploom and iQOS, respectively
**Authors, study year**	**Tabuchi et al., 2017** [32]
**Funder/Affiliations**	**Affiliations:**<br>• Cancer Control Center, Osaka International Cancer Institute, Osaka, Japan<br>• Department of Epidemiology, Laboratory of Lifestyle Epidemiology, Istituto di Ricerche Farmacologiche 'Mario Negri', Milan, Italy

	• Department of Biostatistics, School of Public Health, The University of Tokyo, Bunkyo-ku, Tokyo, Japan<br>• Department of Geography, College of Letters, Ritsumeikan University, Kita-ku, Kyoto, Japan<br>• Department of Environmental Health, National Institute of Public Health, Wako City, Saitama, Japan<br>• School of Public Health, Texas A&M University, College Station, Texas, USA<br>**Funding:**<br>• Health Labour Sciences Research Grants (H26-junkankitou-ippan-023, H28-junkankitou-ippan-002, H28-junkankitou- ippan-008 and H29-tokubetsu-shitei-006)<br>• Japan Society for the Promotion of Science (JSPS) KAKENHI Grants (15H02964 and 15K19256).
**Primary aim**	To assess population interest, rate of use, predictors of use, and perceived effects of second-hand HnB aerosol
**Products used**	• IQOS<br>• Ploom/Ploom TECH<br>• glo
**Methods**	**Design:** Epidemiological study<br>**Data collection:** Longitudinal internet survey and Google Trends analysis<br>**Study time:** Internet survey baseline 31st January–17th February 2015, follow-ups: 29th January–15th February 2016 & 27th January–27th February 2017<br>**Sampling frame:** defined by the Census in Japan<br>**Measures:**<br>• Internet survey: awareness and use of e-cigarettes and heat not burn tobacco products, smoking status, exposure to tobacco-related media information (a question whether participants saw TV program which promoted IQOS products), symptoms from exposure to secondhand HnB tobacco aerosol and other variables (residence area, marital status, education, housing tenure, occupation, self-rated health)<br>• Google trends: to evaluate search activity related to HnB tobacco products, weekly aggregated search trends from Japan were analysed using search terms 'e-cigarettes', 'Ploom', 'IQOS' and 'glo' both in English and Japanese.
**Participants**	**Response rates of eligible participants:** 2015: 8240, 2016: 5366 (65.1%), 2017: 4217 (51.2%)<br>N=8240 (after excluding participants with discrepancies in reported data)
**Key findings**	• The highest relative search volume spike for IQOS in Google was observed in the week of 24–30 April 2016 when IQOS was introduced in the TV show. For Ploom and glo, small spikes were notices corresponding to release times of these products<br>• In 2017, the e-cigarette current user rate had increased to 1.9% (from 1.3% in 2015), while the IQOS current user rate had increased to 3.6% (from 0.3% in 2015). The Ploom Tech current user rate increased to 1.2% (from 0.3% in 2015), and the glo current user rate was 0.8% in 2017.<br>• Respondents who had seen the IQOS promotion on the TV program in April 2016 were significantly more likely to use it than those who had not (10.3% vs 2.7%)
**Authors' conclusions**	• The entertainment TV programme triggered IQOS diffusion in Japan<br>• 3.6% of people currently used IQOS, while 4.7% currently used any HnB or e-cigarette product and 3.4% were dual users

**Authors, study year**	**Brose et al., 2018** [33]
**Funder/Affiliations**	**Affiliations:**<br>• National Addiction Centre, Institute of Psychiatry, Psychology & Neuroscience (IoPPN), London, UK<br>• Action on Smoking and Health, London, UK<br>**Funding:**<br>• Cancer Research UK (CRUK)/BUPA Foundation Cancer Prevention Fellowship (grant number C52999/A19748)
**Primary aim**	To assess awareness and use of HnB products in a nationally representative sample
**Products used**	• IQOS<br>• Ploom/Ploom TECH
**Methods**	**Design:** Epidemiological study<br>**Data collection:** National internet survey<br>**Study time:** February–March 2017<br>**Sampling frame:** defined by 2011 UK Census; large scale probability surveys; results of the 2015 general election; and population estimates from the Office for National Statistics<br>**Measures:** socio-demographics, smoking status, e-cigarette and HnB tobacco products awareness and use
**Participants**	N=12696
**Key findings**	• 9.3% of respondents were aware of HnB tobacco products, 1.7% had or were using them<br>• Never e-cigarette users were more likely to be unaware of HnB products, current e-cigarette triers/ users were more likely to be experimenting with HnB
**Authors' conclusions**	• In 2017 in GB, awareness and use of HnB tobacco products was very low: about 9% were aware and less than 2% had tried or used these products

References

[1] US Food and Drug Administration. Reporting Harmful and Potentially Harmful Constituents in Tobacco Products and Tobacco Smoke Under Section 904(a)(3) of the Federal Food, Drug, and Cosmetic Act. 2012.

[2] Picavet P, Haziza C, Lama N*, et al.* Comparison of the Pharmacokinetics of Nicotine Following Single and Ad Libitum Use of a Tobacco Heating System or Combustible Cigarettes. *Nicotine Tob Res* 2016;**18**(5):557-563.

[3] Brossard P, Weitkunat R, Poux V*, et al.* Nicotine pharmacokinetic profiles of the Tobacco Heating System 2.2, cigarettes and nicotine gum in Japanese smokers. *Regul Toxicol Pharmacol* 2017;**89**(November 2013):193-199.

[4] Yuki D, Sakaguchi C, Kikuchi A*, et al.* Pharmacokinetics of nicotine following the controlled use of a prototype novel tobacco vapor product. *Regul Toxicol Pharmacol* 2017;**87**:30-35.

[5] Ludicke F, Baker G, Magnette J*, et al.* Reduced Exposure to Harmful and Potentially Harmful Smoke Constituents With the Tobacco Heating System 2.1. *Nicotine Tob Res* 2017;**19**(2):168-175.

[6] Ludicke F, Haziza C, Weitkunat R*, et al.* Evaluation of Biomarkers of Exposure in Smokers Switching to a Carbon-Heated Tobacco Product: A Controlled, Randomized, Open-Label 5-Day Exposure Study. *Nicotine Tob Res* 2016;**18**(7):1606-1613.

[7] Lopez AA, Hiler M, Maloney S*, et al.* Expanding clinical laboratory tobacco product evaluation methods to loose-leaf tobacco vaporizers. *Drug Alcohol Depend* 2016;**169**:33-40.

[8] Haziza C, de La Bourdonnaye G, Merlet S*, et al.* Assessment of the reduction in levels of exposure to harmful and potentially harmful constituents in Japanese subjects using a novel tobacco heating system compared with conventional cigarettes and smoking abstinence: A randomized controlled study in confinement. *Regul Toxicol Pharmacol* 2016;**81**:489-499.

[9] Haziza C, de La Bourdonnaye G, Skiada D*, et al.* Evaluation of the Tobacco Heating System 2.2. Part 8: 5-Day randomized reduced exposure clinical study in Poland. *Regul Toxicol Pharmacol* 2016;**81 Suppl 2**:S139-S150.

[10] Ludicke F, Picavet P, Baker G*, et al.* Effects of Switching to the Tobacco Heating System 2.2 Menthol, Smoking Abstinence, or Continued Cigarette Smoking on Biomarkers of Exposure: A Randomized, Controlled, Open-Label, Multicenter Study in Sequential Confinement and Ambulatory Settings (Part 1). *Nicotine Tob Res* 2017(February):1-10.

[11] Ludicke F, Picavet P, Baker G*, et al.* Effects of Switching to the Menthol Tobacco Heating System 2.2, Smoking Abstinence, or Continued Cigarette Smoking on Clinically Relevant Risk Markers: A Randomized, Controlled, Open-Label, Multicenter Study in Sequential Confinement and Ambulatory Settings (Part 2). *Nicotine Tob Res* 2017(February):1-10.

[12] Gee J, Prasad K, Slayford S*, et al.* Assessment of tobacco heating product THP1.0. Part 8: Study to determine puffing topography, mouth level exposure and consumption among Japanese users. *Regul Toxicol Pharmacol* 2017.

[13] Auer R, Concha-Lozano N, Jacot-Sadowski I*, et al.* Heat-Not-Burn Tobacco Cigarettes: Smoke by Any Other Name. *JAMA Intern Med* 2017;**177**(7):1050-1052.

[14] Vu AT, Taylor KM, Holman MR*, et al.* Polycyclic Aromatic Hydrocarbons in the Mainstream Smoke of Popular U.S. Cigarettes. *Chem Res Toxicol* 2015;**28**(8):1616-1626.

[15] Farsalinos KE, Yannovits N, Sarri T*, et al.* Nicotine delivery to the aerosol of a heat-not-burn tobacco product: comparison with a tobacco cigarette and e-cigarettes. *Nicotine Tob Res* 2017.

[16] Bekki K, Inaba Y, Uchiyama S*, et al.* Comparison of Chemicals in Mainstream Smoke in Heat-not-burn Tobacco and Combustion Cigarettes. *J UOEH* 2017;**39**(3):201-207.

[17] Schaller JP, Keller D, Poget L*, et al.* Evaluation of the Tobacco Heating System 2.2. Part 2: Chemical composition, genotoxicity, cytotoxicity, and physical properties of the aerosol. *Regul Toxicol Pharmacol* 2016;**81 Suppl 2**:S27-S47.

[18] Schaller JP, Pijnenburg JP, Ajithkumar A*, et al.* Evaluation of the Tobacco Heating System 2.2. Part 3: Influence of the tobacco blend on the formation of harmful and potentially harmful constituents of the Tobacco Heating System 2.2 aerosol. *Regul Toxicol Pharmacol* 2016;**81 Suppl 2**:S48-S58.

[19] Protano C, Manigrasso M, Avino P*, et al.* Second-hand smoke exposure generated by new electronic devices (IQOS(R) and e-cigs) and traditional cigarettes: submicron particle behaviour in human respiratory system. *Ann Ig* 2016;**28**(2):109-112.

[20] Ruprecht AA, De Marco C, Saffari A*, et al.* Environmental pollution and emission factors of electronic cigarettes, heat-not-burn tobacco products, and conventional cigarettes. *Aerosol Science and Technology* 2017;**51**(6):674-684.

[21] Mitova MI, Campelos PB, Goujon-Ginglinger CG*, et al.* Comparison of the impact of the Tobacco Heating System 2.2 and a cigarette on indoor air quality. *Regul Toxicol Pharmacol* 2016;**80**:91-101.

[22] O'Connell G, Wilkinson P, Burseg KMM*, et al.* Heated Tobacco Products Create Side-Stream Emissions: Implications for Regulation. *Journal of Environmental Analytical Chemistry* 2015;**02**(05):2-4.

[23] Eaton D, Jakaj B, Forster M*, et al.* Assessment of tobacco heating product THP1.0. Part 2: Product design, operation and thermophysical characterisation. *Regul Toxicol Pharmacol* 2017.

[24] Forster M, Fiebelkorn S, Yurteri C*, et al.* Assessment of novel tobacco heating product THP1.0. Part 3: Comprehensive chemical characterisation of harmful and potentially harmful aerosol emissions. *Regul Toxicol Pharmacol* 2017.

[25] Forster M, McAughey J, Prasad K*, et al.* Assessment of tobacco heating product THP1.0. Part 4: Characterisation of indoor air quality and odour. *Regul Toxicol Pharmacol* 2017.

[26] Protano C, Manigrasso M, Avino P*, et al.* Second-hand smoke generated by combustion and electronic smoking devices used in real scenarios: Ultrafine particle pollution and age-related dose assessment. *Environ Int* 2017;**107**:190-195.

[27] Jaccard G, Tafin Djoko D, Moennikes O*, et al.* Comparative assessment of HPHC yields in the Tobacco Heating System THS2.2 and commercial cigarettes. *Regul Toxicol Pharmacol* 2017;**90**:1-8.

[28] Poynton S, Sutton J, Goodall S*, et al.* A novel hybrid tobacco product that delivers a tobacco flavour note with vapour aerosol (Part 1): Product operation and preliminary aerosol chemistry assessment. *Food Chem Toxicol* 2017;**106**(Pt A):522-532.

[29] Pratte P, Cosandey S, Goujon Ginglinger C. Investigation of solid particles in the mainstream aerosol of the Tobacco Heating System THS2.2 and mainstream smoke of a 3R4F reference cigarette. *Hum Exp Toxicol* 2017;**36**(11):1115-1120.

[30] Haziza C, de La Bourdonnaye G, Skiada D*, et al.* Biomarker of exposure level data set in smokers switching from conventional cigarettes to Tobacco Heating System 2.2, continuing smoking or abstaining from smoking for 5 days. *Data Brief* 2017;**10**:283-293.

[31] Tabuchi T, Kiyohara K, Hoshino T*, et al.* Awareness and use of electronic cigarettes and heat-not-burn tobacco products in Japan. *Addiction* 2016;**111**(4):706-713.

[32] Tabuchi T, Gallus S, Shinozaki T*, et al.* Heat-not-burn tobacco product use in Japan: its prevalence, predictors, and perceived symptoms from exposure to secondhand heat-not-burn-tobacco aerosol. *Tobacco Control* 2017:1-9.

[33] Brose L, Simonavicius E, Cheeseman H. Awareness and Use of 'Heat-not-burn' Tobacco Products in Great Britain. *Tobacco Regulatory Science* 2018;**4**(2):44-50.
